# Correlation of Blood Biochemical Markers with Tardive Dyskinesia in Schizophrenic Patients

**DOI:** 10.1155/2022/1767989

**Published:** 2022-03-08

**Authors:** Qian Wu, Fengjuan Yuan, Shuo Zhang, Weiting Liu, Qing Miao, Xinhua Zheng, Suxiang Lu, Kaijian Hou

**Affiliations:** ^1^School of Medicine, Pingdingshan University, Pingdingshan City 467000, China; ^2^Department of Endocrine and Metabolic Diseases, Longhu Hospital, The First Affiliated Hospital of Shantou University Medical College, Shantou City 515041, China; ^3^School of Nursing, Anhui University of Chinese Medicine, Hefei City 230012, China; ^4^Department of Physical Education, Henan University of Urban Construction, Pingdingshan City 467000, China

## Abstract

**Objective:**

Oxidative stress factors and proinflammatory cytokines had been found to be involved in the pathogenesis of patients with tardive dyskinesia (TD). This study assumes that blood biochemical markers would have a link with TD in schizophrenia patients. To explore the correlation between blood biochemical markers and tardive dyskinesia in patients with schizophrenia (SCH).

**Methods:**

From January 2010 to August 2021, the inpatients who met the diagnostic criteria of schizophrenia in the Chinese Classification and Diagnosis Criteria of Mental Disorders (DSM-4) and the American Diagnostic and Statistical Manual of Mental Disorders (DSM-4) were followed up in the psychiatric outpatient department of Jinxia Street Community Health Service Center, Longhu District, Shantou City. The diagnostic criteria of Abnormal Involuntary Movement Scale (AIMS) used in the TD study of Schooler and Kane were used to screen the patients. Patients were divided into the schizophrenia (SCH group) and the schizophrenia with TD groups (TD group). Oxidative stress factors including Superoxide Dismutase1 (SOD1), Glutathione Peroxidase1 (GPX1), Malondialdehyde1 (MDA1), Catalase Activity1 (CAT1), and brain-derived neurotrophic factor 1 (BDNF1) and some inflammatory cytokines including interleukin-2 (IL-2), interleukin-6 (IL-6), interleukin-8 (IL-8), serum tumor necrosis factor (TNF-*α*), prolactin, estrogen, and cortisol were measured in 121 schizophrenic patients with tardive dyskinesia and 118 schizophrenic patients. The correlation analysis was conducted on the data.

**Results:**

Age and female were immutable risk factors for the development of TD, and there were significant differences in blood biochemical indices GPX1, MDA1, CAT1, and TNF-*α* in schizophrenic patients with and without TD.

**Conclusion:**

This study supports that oxidative stress and immune disorders are associated with TD patients. Blood biochemical markers GPX1, MDA1, CAT1, and TNF-*α* may play an important role in the pathogenesis of schizophrenia combined with TD patients, and they may be useful in the diagnosis of schizophrenia with tardive dyskinesia.

## 1. Introduction

Long-term use of antipsychotics can lead to tardive dyskinesia (TD), an abnormal and persistent rigid repetitive involuntary movement disorder in patients [1, 2]. TD is a serious drug side effect with persistent, long-lasting, and often permanent symptoms. The most prominent symptom is the involuntary movement of the mouth, lips, tongue, and face, known as the oral-tongue-buccal triad, manifested by licking the tongue and swelling the cheek, sometimes accompanied by dancing movements of the torso and limbs or finger-like movements [3, 4]. At present, the etiology of TD is not clear, and the pathogenesis is complex, involving multiple causes.

The pathogenesis of TD is mostly centered in the dopamine (DA) hypersensitivity theory and neuronal degeneration hypothesis [5]. Antipsychotic drugs can block the DA receptors in the brain, and the massive accumulation of DA will generate hydroxyl free radicals and superoxide anions with relatively strong oxidative capacity. The generation of strong oxide will increase the attack on the DA neurons in the extrapyramidal system of the brain and cause the apoptosis and degeneration of neurons [6]. According to PubMed, Web of Science, and other databases on the pathogenesis of TD literature review [7], in addition to the dopamine (DA) hypersensitivity theory and neuronal degeneration hypothesis described above, the pathogenesis of TD also involves oxidative stress, estrogen receptor activation [8], immune inflammatory response [9], and brain microelement iron [10]. Oxidative stress has a significant impact on TD, and more and more recent studies have found that some strong antioxidants, such as melatonin (MT) [11], vitamin E, VMAT2 inhibitor, and other antioxidants, have a significant easing effect on the clinical manifestations of TD [7]. Many people have studied the lipid peroxidation and indicated the involvement of oxidative stress in TD patients [12]. The oxidative stress factors superoxide dismutase (SOD), glutathione peroxidase (GPX), catalase activity (CAT), and malondialdehyde (MDA) are major targets for TD patients. Brain-derived neurotrophic factor (BDNF) plays an important role in the resistance to oxidation and neuronal survival, growth, and differentiation, and the level of BDNF is obviously concerned in the treatment of TD [13, 14]. Studies on immune inflammation mechanism in SCH and TD patients found chronic inflammation [15]. Proinflammatory cytokines such as interleukin-2 (IL-2), interleukin-6 (IL-6), interleukin-8 (IL-8), and serum tumor necrosis factor (TNF-*α*) were significantly changed in SCH and TD patients [16]. Prolactin, estrogen, and cortisol are also involved in the pathogenesis of TD, and their values have undergone significant changes in patients [8, 9]. Because the pathogenesis of TD is uncertain, most studies have focused on one possible pathogenesis and have not integrated all the many factors that may be involved in the pathogenesis. The majority of studies have focused on one possible pathogenesis, without integrating all the many factors that may be involved in the pathogenesis. This has led to the neglect of the role of oxidative stress factors when considering the dopamine hypothesis and the influence of inflammatory factors when considering oxidative stress mechanisms.

Therefore, it is of great significance to study whether blood biomarkers of schizophrenic patients with tardiness dyskinesia can be used as diagnostic markers of TD. In this study, the correlation between biomarkers and patients with schizophrenia with TD was investigated.

## 2. Materials and Methods

### 2.1. Study Subjects

Patients were selected from the psychiatric outpatient clinic of Jinxia Street Community Health Service Center, Longhu District, Shantou City from January 2020 to August 2021. All patients in the study met the diagnostic criteria of schizophrenia in the Chinese Classification and Diagnosis criteria of Mental Disorders (3RD edition) and the American Diagnostic and Statistical Manual of Mental Disorders (4TH edition). Patients were aged 18-70 years, and the duration of disease was at least 6 months. The type and dose of antipsychotic drugs did not change in the first 4 weeks. The diagnostic criteria for TD were by Schooler and Kane, namely, the Abnormal Involuntary Movement Scale (AIMS) score of at least one or at least two excluding hypomanic episodes and manic episodes. The following patients were excluded from the study: (1) those with substance dependence and other mental disorders; (2) those with serious physical diseases and endocrine diseases; (3) those in lactation or pregnancy; and (4) those with posttraumatic stress disorder. Patients were divided into the TD group and the SCH group according to whether TD was concomitant or not. There were 121 patients in the TD group and 118 patients in the SCH group. The mean age of onset was 24.68 ± 6.14 years. The total course of disease was 30.09 ± 8.27 years, and the total score (PANSS score) was 78.15 ± 14.68 points. Of all the 118 SCH patients, 71 were males and 47 were females. The mean onset age was 25.91 ± 8.76 years, and the total course of disease was 21.53 ± 9.73 years.

This study was approved by the Ethics Committee of the Hospital, and all patients and their families were informed and signed the informed consents (medical ethics no.: jxsk202101).

### 2.2. Research Methods

#### 2.2.1. Demographic and Clinical Data Collection

The general questionnaire, which included information of age, sex, BMI, years of education, age of onset, physical diseases, and total course of disease, was self-made to collect the demographic and clinical data of the subjects.

#### 2.2.2. Clinical Evaluation

Two senior clinicians evaluated the positive and negative symptoms of patients of the two included groups using the AIMS scale used in the TD study by Schooler and Kane. The total PANSS score was used as the index of disease severity in the TD group and the SCH group.

#### 2.2.3. Detection of Biological Markers

Fasting peripheral blood was collected in the morning of the second day after admission. The peripheral blood was placed in a coagulation tube for 30 minutes, followed by centrifugation at 3000 r/min for 15 minutes. Serum was extracted and stored in a refrigerator at minus 40 degrees Celsius. SOD1, GPX1, MDA1, CAT1, and BDNF1 were detected by electrochemical photoimmunoassay. The levels of prolactin, IL-2, IL-6, IL-8, TNF-*α*, estrogen, and cortisol were measured using the German Roche automatic electrochemiluminescence immunoanalyzer. All reagents were purchased from Roche (Germany). The experimental procedures were carried out according to kit instructions. All samples were tested by the same laboratory and the same analyst.

### 2.3. Statistical Analysis

IBM SPSS 20.0 statistical software was used for data analysis. Data records are expressed as $\text{mean }\left(\bar{\chi }\right)+\text{standard deviation}\left(\text{s}\right)$. Data of patients in the TD and the SCH groups were compared using *t*-tests for the continuous variables and chi-square tests for the categorical variables. Correlation analysis was performed by Pearson's test. *P* < 0.05 was considered statistically significant.

## 3. Results and Discussion

### 3.1. The Demographic and Clinical Data of Patients in the TD and the SCH Groups

There were statistically significant differences in age, gender, years of education, diabetes, total course of disease, P1 total score, N1 total score, G1 total score, and PANSS score between the TD and the SCH group (*P* < 0.05) (Table 1). Age, prevalence of diabetes, total course of disease, and P1, N1, G1, and PANSS scores in the TD group were significantly higher than those in the SCH group (*P* < 0.05) (Table 1). Compared with SCH, TD was more common in males than in females (*P* < 0.05) (Table 1). The TD population had fewer years of education than the SCH population (*P* < 0.05) (Table 1).

### 3.2. Comparison of Oxidative Stress Factors SOD1, GPX1, MDA1, CAT1, and BDNF1 between the TD and the SCH Population

**Figure 1:**
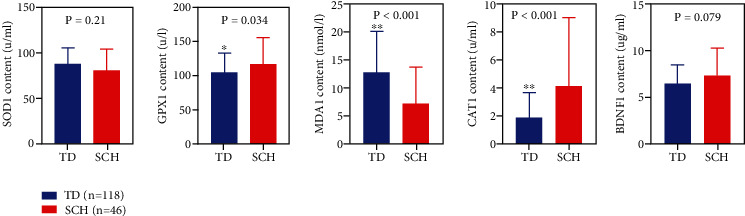
Comparison of oxidative stress factors (*P* < 0.05) between the TD and the SCH groups. Note: TD group—*n* = 118; SCH group—*n* = 46. ∗ represents *P* < 0.05 and ∗∗ represents *P* < 0.01, compared with TD groups.

GPX1, MDA1, and CAT1 were statistically different between the TD and the SCH groups (*P* < 0.05) (Figure 1). There was no statistical difference in the blood level of SOD1 and BDNF1 between the TD and the SCH groups (*P* > 0.05) (Figure 1). The MDA1 value in the TD group was significantly higher than that in the SCH group (*P* < 0.05) (Figure 1). The GPX1 and CAT1 values in the SCH population were significantly higher than those in the TD population.

### 3.3. Comparison of Inflammatory Factors between the TD and the SCH Population

**Figure 2:**
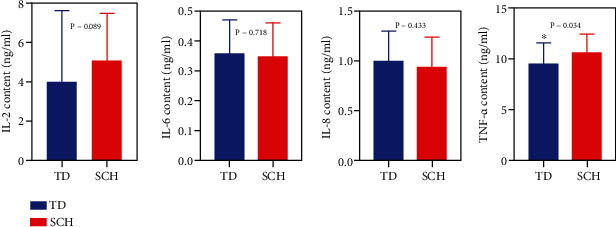
Comparison of inflammatory factors between the TD and the SCH groups (*P* < 0.05). Note: TD group—*n* = 46; SCH group—*n* = 46. ∗ represents *P* < 0.05 and ∗∗ represents *P* < 0.01, compared with TD groups.

We evaluated the blood levels of four proinflammatory factors, IL-2, IL-6, IL-8, and TNF-*α*. It was found that there were significant differences between the TD and the SCH patients (*P* < 0.05) (Figure 2). The TNF-*α* level in the TD group was significantly lower than that in the SCH group. There were no significant differences in the levels of IL-2, IL-6, and IL-8 between the two groups.

**Figure 3:**
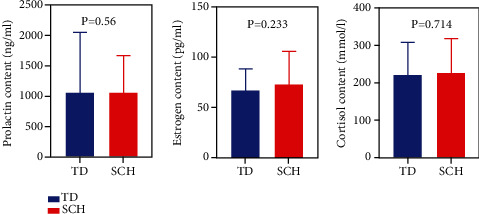
Comparison of hormone values between the TD and the SCH population (*P* < 0.05). Note: TD group—*n* = 46; SCH group—*n* = 46. ∗ represents *P* < 0.05 and ∗∗ represents *P* < 0.01, compared with TD groups.

The blood levels of prolactin, estrogen, and cortisol may be involved in the pathogenesis of TD and were analyzed and compared between the TD population and the SCH population. There was no statistical difference in these three indexes between the two groups (*P* > 0.05) (Figure 3).

### 3.4. Discussion

The results of this study showed that age, prevalence of diabetes, total course of disease, and P1, N1, G1, and PANSS scores in the TD group were significantly higher than those in the SCH group (*P* < 0.05). Compared with SCH, TD was more commonly occurred in males than in females (*P* < 0.05). The TD population had fewer years of education than SCH population (*P* < 0.05).

When evaluating the risk factors of TD, Vardar et al. [17] demonstrated that the risk factors of TD were higher in patients with mental illness at the age of 35 and older from the perspective of demography. Smoking, alcohol, and drug abuse are also risk factors that are consistent with the analysis results of TD-related risk factors conducted by Solmi et al. [18]. Solmi et al. also suggested that there were other risk factors associated with TD, including old age, female, and white race. Advanced age is a risk factor for TD, either because the body's antioxidant capacity decreases with aging or because elderly patients have slow metabolic rate [19]. The peroxidation effect caused by antipsychotic drugs may result in the deficiency of essential fatty acids and the damage of striatum neurons in brain tissues. Women are less likely to suffer from TD, most likely because there are higher levels of estrogen, which can adjust the stability of DA-R in the central nervous system, thereby reducing the occurrence of TD or alleviate symptoms of TD. Most women in this study are elderly and might have low ovarian function and low level of estrogen. Related studies have pointed out that estrogen has a very important role in regulating cognitive functions including learning, attention, and memory and can affect the structure and function of brain regions. Datta et al. [20, 21] investigated the effect of estrogen on several spatial learning memory abilities of cognitive functions in the central nervous system of TD rat model by methamphetamine intervention through animal experiments. The results showed that estrogen could improve the memory impairment and spatial learning impairment in TD rats to some extent. The significantly higher estrogen values in the TD and SCH populations than in the HC population in the present study may be related to the fact that liver damage caused by drug administration in the TD and SCH populations affected the ability of the liver to inactivate estrogen. Chatterjee and Sharan [22] pointed out that estrogen was able to improve the clinical symptoms of low-dose, short-term risperidone-induced TD in pregnant women or and estrogen-induced dopamine hypersensitivity response is related.

There is also a significant difference in the number of years of education between the patients in the TD group and those in the SCH group. The brain is active during learning, which can promote the development of thinking. The longer the education time, the greater the impact on the brain, thus promoting the activity of nerve cells. Samad and Haleem [23] verified the antioxidant effect of rice bran oil on reducing TD induced by haloperidol in male rats through animal experiments. The results showed that the therapeutic effect of rice bran oil on TD could be verified by changing the levels of malondialdehyde (MDA), hydrogen peroxide (H_2_O_2_), and antioxidant enzymes superoxide dismutase (SOD), catalase (CAT), and glutathione peroxidase (GPX). The results also showed that GPX1 value in TD population was significantly higher than that in SCH population (*P* < 0.05). The CAT1 value in SCH population was significantly higher than that in TD population. GPX1 is a selenium-dependent enzyme that plays an important role in free radical detoxification [24]. Shinkai et al. [13] studied the genetic association between glutathione peroxidase gene polymorphism (Pro197Leu) and tardive dyskinesia. GPX1 is clearly involved in the pathogenesis of TD, but GXP1 polymorphism does not increase TD susceptibility. Xiang et al. [25] showed that BDNF may be involved in the pathogenesis and pathophysiology of schizophrenia and the potential correlation between BDNF gene polymorphism in Chinese Han population and susceptibility to schizophrenia and the psychopathological symptoms of schizophrenia patients. BDNF gene variation may influence the severity of clinical symptoms in schizophrenia patients to a certain extent. Wu et al. [26] monitored TAS and malondialdehyde (MDA) levels in determining the oxidative stress state reflected by CuZnSOD in male TD patients and showed that MDA levels in TD patients were significantly higher than those in non-TD patients. It was further confirmed that TD patients had higher lipid peroxidation level due to more severe oxidative stress and thus showed significantly increased MDA level. The results of our study are inconsistent with this finding. Lv et al. [27] found that ginkgo biloba leaf extract has significant therapeutic effect on male patients with TD in their study on the therapeutic effect of ginkgo biloba leaf on tardive dyskinesia in men. BDNF can promote nerve cell differentiation, and the increase of BDNF value can alleviate the patient's condition. However, in this study, although BDNF decreased in TD patients, there was no significant difference between the TD and SCH patients.

Previous studies have found that inflammatory cytokines in the blood of patients with schizophrenia are elevated to varying degrees, which may be related to the presence of neuroinflammation in patients [28] Sun et al. [29] monitored the serum levels of IL-2, IL-4, IL-6, IL-10, and IL-17 in patients with schizophrenia at the time of initial treatment and found that serum IL-6 levels in patients with SCH were significantly increased and IL-6 level could be used as a marker for the diagnosis of schizophrenia. Ghazvini et al. [30] treated the haloperidol-induced TD rat model with onsolene, and the results showed that onsolene could significantly reduce nerve injury and improve movement disorders in TD rats. Biochemical parameters used to evaluate the interventive effect mainly included MDA, IL-6, and TNF-*α*. In this study, the TNF-*α* value of TD population was significantly lower than that of SCH population. The blood levels of prolactin, IL-2, IL-6, IL-8, and estrogen were not statistically different between the TD and SCH groups. In previous studies on TD patients, the level of IL-2 was increased and that of IL-6 was decreased compared with those in non-TD patients, and proinflammatory cytokines and anti-inflammatory cytokines were disturbed in TD patients [16, 30]. Bergman et al. [31] studied the treatment using antipsychotic drug benzodiazepine in TD patients. They found an imbalance between anti-inflammatory cytokines and proinflammatory cytokines in TD patients. The changed level of IL-2 and IL-6 in this study was inconsistent with previous studies, which might be related to the small sample size included in the study.

TNF-*α* is a polypeptide cytokine with various biological activities produced by activated mononuclear macrophages. It is closely associated with the acute phase of immune response and inflammatory response and has a wide range of biological activities. It is the main inflammatory mediator. Rodrigues-Amorim et al. [32] carried out a review study on cytokines related to schizophrenia patients; they also mentioned that the TNF-*α* level of psychiatric patients was significantly reduced and the immune function was in an unbalanced state. The study results of TD showed that the level of TNF-*α* is significantly below normal and significantly lower than that of SCH patients. This finding is consistent with the previous research and further shows that TNF-*α* may be involved in the pathogenesis of TD. However, the role of TNF-*α* in TD patients in the pathogenic process of and the relationship with other factors is uncertain and needs to be further discussed.

## 4. Conclusions

The main findings of this study were as follows: (1) Patients in the TD group had higher age, higher prevalence of diabetes, total course of disease, Positive and Negative Syndrome Scale (PANSS) scores, delusions (P1), blunted affect (N1), somatic concern (G1) than the SCH group; the difference was statistically significant (*P* < 0.05). TD is more common in males than in females (*P* < 0.05). TD population had lower education level than SCH population (*P* < 0.05). (2) MDA1 values in the TD group were significantly higher than those in the SCH group (*P* < 0.05). The value of GPX1 and CAT1 in the TD group was significantly lower than that in the SCH group (*P* < 0.05). (3) TNF-*α* in the TD group was significantly lower than that in the SCH group (*P* < 0.05).

Several limitations of this study should be noted here. First, because the patients involved in this study were patients on long-term antipsychotic medications and previous studies have found that typical and atypical antipsychotics have a significant effect on blood interleukins [33], we should consider the possible effects of antipsychotics on blood biochemical markers in our study. We paid more attention to the blood biochemical markers of patients in our study, but TD is with obvious behavioral abnormalities, and we can pay more attention to the correlation between blood biochemical markers and the severity of abnormal behavior in future studies, and the cognitive status of TD patients has correlation with blood biochemical markers, so the role of blood biochemical markers in the pathophysiological mechanism of TD needs further study.

## Figures and Tables

**Table 1:** The demographic and clinical data covered by the study ($\bar{\chi }\pm s$).

The crowd characteristics	TD (*n* = 121)	SCH (*n* = 118)	*P*
Age	54.66 ± 7.80	47.66 ± 8.54	<0.001
Gender (female/male)	89/32	71/47	0.028
BMI(kg/m2)	24.41 ± 3.65	24.62 ± 4.29	0.706
Years of education(year)	9.28 ± 2.52	10.42 ± 2.68	0.001
Diabetes (%)	21 (17.5)	9 (7.6)	0.022
Age of onset	24.68 ± 6.14	25.91 ± 8.76	0.212
The total course	30.09 ± 8.27	21.53 ± 9.73	<0.001
P1 score	16.49 ± 5.61	14.90 ± 6.78	0.049
N1 score	26.07 ± 6.01	22.85 ± 5.97	<0.001
G1 score	35.59 ± 8.04	30.81 ± 8.09	<0.001
PANSS score	78.15 ± 14.68	68.56 ± 16.54	<0.001

## Data Availability

The raw file has been submitted in the attachment.

## Supplementary Materials

Supplementary Materials
*Material Description.* MCCB date file is the data related to the MATRICS Consensus Cognitive Battery (MCCB) performed during the follow-up of inpatients who met the diagnostic criteria for schizophrenia in the Chinese Classification and Diagnosis of Mental Disorders (DSM-4) and the American Diagnostic and Statistical Manual of Mental Disorders (DSM-4) at the psychiatric outpatient department of Jinxia Street Community Health Center, Longhu District, Shantou City, from January 2010 to August 2021 and is the data underlying the analysis in this article.
