# Supplementary material for: Correlation of Blood Biochemical Markers with Tardive Dyskinesia in Schizophrenic Patients
Source: Dis Markers. 2022 Mar 8;2022:1767989. doi: 10.1155/2022/1767989 (PMC8923804; doi:10.1155/2022/1767989)
Supplement: Supplementary Materials — Material Description. MCCB date file is the data related to the MATRICS Consensus Cognitive Battery (MCCB) performed during the follow-up of inpatients who met the diagnostic criteria for schizophrenia in the Chinese Classification and Diagnosis of Mental Disorders (DSM-4) and the American Diagnostic and Statistical Manual of Mental Disorders (DSM-4) at the psychiatric outpatient department of Jinxia Street Community Health Center, Longhu District, Shantou City, from January 2010 to August 2021 and is the data underlying the analysis in this article. [file 1767989.f1.pdf]

**name**	
**group**	
**gender**	
**age**	
**edu**	
**marry**	
**heredity**	
**diagtype**	
**sympage**	
**hospage**	
**tcourse**	
**course**	
**smoking**	
**height**	
**waist**	
**hip**	
**waisthip**	
**weight**	
**bmi**	
**p**	
**n**	
**g**	
**panss**	
**cgi**	
**hamd**	
**glu**	
**glu2h**	
**IGT**	impaired glucose tolerance
**IFG**	impaired fasting glucose
**insuli**	
**IR**	
**cho**	
**tg**	
**hdl**	
**ldl**	
**agegroup**	
**glub**	
**glu2b**	
**IFGb**	impaired fasting glucose after treatment
**IGTb**	impaired glucose tolerance after treatment
**pb**	SumPAN7P positive subscale total score
**nb**	SumPAN7N negative subscale total score
**gb**	SumPAN7G General psychopathology subscale tota
**panssb**	SumPANS7 PANSS total score
**hamdb**	S7umHAMD HAMD total score
**cgib**	C7GI1c Disease severity
**chob**	CHO7 Total cholesterol(mmol/L)
**tgb**	TG7 Triglycerides(mmol/L)
**hdlb**	

**ldlb**	
**bmib**	
**waistb**	
**hipb**	
**weightb**	
**insulib**	
**IRb**	
**waisthipb**	
**tmt**	Connected fractions
**SC**	Symbolic coding scores
**hvlt**	Verbal memory score
**ss**	Spatial breadth score
**ds**	Number sequence score
**nab**	Maze score
**maze**	Maze score
**bvmt**	Visual Memory Score
**cf**	Verbal Fluency Score
**eit**	Emotional Management Score
**cptd**	Continuous Operations Test Score
**MCCB Total Sore**	
**stroop**	Stroop Score
**ep**	Emotional Recognition Score
**dspan**	Breadth of Numbers Score
**upsa score**	
**Linking T points**	TMT
**Symbolic coding T-score**	SC
**Verbal memory T-score**	hvlt
**Spatial breadth T-score**	SS
**Maze T-score**	maze (NAB)
**Visual Memory T Score**	bvmt
**Verbal Fluency T Score**	cf
**Emotion Management Ability T Sco**	eit
**Continuous operational ability T sc**	cptd
**Digit Sequence T Score**	ds
**UPSA China Standing Model T Score**	

al score

name	group	gender	age	edu	marry	eredity	agtype	mpage	ospage	course
季博文	1.00	1	16	9	1	1	21	16	16	2.00
李世强	1.00	1	16	9	1	2	21	15	16	10.00
张赛	1.00	1	16	8	1	2	20	15	16	5.00
班兆龙	1.00	1	17	8	1	2	21	16	16	1.00
代玲月	1.00	1	17	11	1	1	21	16	17	1.00
丁铄	1.00	1	17	10	1	1	21	17	17	3.30
贺建新	1.00	1	17	11	1	2	20	16	17	8.00
肖爽	1.00	1	17	11	1	1	20	17	17	.10
鲍东	1.00	1	18	12	1	1	21	18	18	3.00
刘俊龙	1.00	1	18	12	1	1	20	18	18	4.00
尚乐	1.00	1	18	7	1	2	21	14	18	4.00
田贺	1.00	1	18	11	1	2	21	15	17	31.00
赵建琛	1.00	1	18	12	1	1	21	16	17	24.00
陈广广	1.00	1	19	9	1	2	21	17	18	24.00
勾浩同	1.00	1	19	10	1	2	21	19	19	2.00
郝羽曾	1.00	1	19	9	1	2	21	19	19	24.00
黄超	1.00	1	19	9	1	2	21	19	19	.50
江苏明	1.00	1	19	9	1	2	20	18	19	12.00
蒋兆星	1.00	1	19	13	1		21	18	19	8.00
唐珂	1.00	1	19	10	1	2	21	17	17	27.00
王帅	1.00	1	19	12	1	2	20	18	19	21.00
许云凯	1.00	1	19	13	1	2	20	19	19	1.00
陈雨	1.00	1	20	13	1	2	20	17	20	36.00
刘南南	1.00	1	20	14	1	2	21	17	20	36.00
马崇旭	1.00	1	20	14	1	2	20	20	20	1.00
王叡	1.00	1	20	12	1	1	21	16	20	40.00
王尧尧	1.00	1	20	14	1	2	20	20	20	2.00
霍辰	1.00	1	21	15	1	2	21	17	20	4.00
姜波	1.00	1	21	11	1	1	21	16	18	60.00
李喜锋	1.00	1	21	12	1	2	21	18	19	36.00
唐正研	1.00	1	21	11	1	2	20	21	21	.30
吴朝阳	1.00	1	21	15	1	2	21	21	21	4.00
李文广	2.00	1	21	9						
刘宝珠	1.00	1	22	9	1	2	21	18	22	48.00
孙飞	1.00	1	22	14	1	2	20	20	22	21.00
杨杰	1.00	1	22	24	1	1	20	23	23	5.00
张科	1.00	1	22	18	1	2	21	22	22	.10
孙志杨	2.00	1	22	15						
廖清	1.00	1	23	16	1	2	21	20	23	37.00
刘焕振	1.00	1	23	11	1	2	21	16	16	72.00
王建波	1.00	1	23	12	1	2	21	23	23	26.00
王立斌	1.00	1	23	15	1	1	21	17	19	72.00
李友峰	1.00	1	24	9	1	2	20	21	24	36.00
姚金鑫	1.00	1	24	12	1	2	21	20	21	9.00
张亮	1.00	1	24	14	1	1	21	24	24	2.00

张栩文	1.00	1	24	15	1	1	20	24	24	6.00
谢景强	2.00	1	24	16						
段彦勇	1.00	1	26	14	1	2	21	24	26	13.00
葛家祥	1.00	1	26	16	1	1	20	23	26	36.00
李国玉	1.00	1	26	9	1	2	20	24	26	28.00
王一平	1.00	1	26	12	1	2	20	24	26	26.00
阴琦山	1.00	1	26	12	1	2	21	24	26	24.00
左楠	1.00	1	27	15	1	2	21	27	27	10.00
焦艳凛	2.00	1	27	15						
杨旭	2.00	1	27	16						
来原	1.00	1	28	18	1	1	20	27	28	19.00
王建武	1.00	1	28	22	2	2	20	27	27	3.00
王林	1.00	1	28	16	1	1	20	25	25	36.00
王亚辉	1.00	1	28	9	2	2	20	25	25	36.00
蔡壮	2.00	1	28	16						
韩瑞俊	2.00	1	28	16						
马传领	1.00	1	29	9	2	2	20	29	29	1.00
孙克	2.00	1	29	15						
赵冉	1.00	1	30	18	2	2	21	30	30	1.00
吕广有	2.00	1	30	16						
孙文亮	1.00	1	31	18	1	1	20	30	30	6.00
申海龙	1.00	1	32	12	3	2	20	32	32	.30
俞凯	1.00	1	32	16	1	2	20		32	
赵政普	1.00	1	32	11	2	1	20	30	33	33.00
朱海红	1.00	1	32	9	2	2	20	32	32	1.00
杨玉涛	1.00	1	33	12	1	1	20	28	33	60.00
张洪泉	1.00	1	33	12	2	2	21	31	33	24.00
赵继江	1.00	1	33	19	1	2	21	29	29	39.00
门振东	2.00	1	33	15						
周艳青	1.00	1	34	12	1	1	21	30	34	48.00
陈溯鹏	1.00	1	36	16	1	2	21	35	36	8.00
马勇	1.00	1	36	9	1	1	20	36	36	4.00
毛琰	1.00	1	36	17	2	2	33	34	34	24.00
吴长林	1.00	1	36	16	2	2	21	35	36	13.00
管楚明	1.00	1	37	3	1	2	20	33	37	41.00
张效禄	1.00	1	37	3	2	2	21	31	31	60.00
马玉山	1.00	1	38	12	1	2	20	38	38	1.00
王涛	1.00	1	38	16	2	2	20	35	36	30.00
褚志强	1.00	1	39	9	1	2	21	36	39	36.00
郝杰中	1.00	1	39	16	2	2	20	34	39	60.00
韩勇	2.00	1	39	15						
周立巍	1.00	1	40	16	2	1	21	40	40	5.00
朱红	1.00	1	40	16	2	2	20	40	40	3.00
曹文胜	2.00	1	40	19						
范林	1.00	1	43	12	3	2	20	41	43	30.00

谢杰	1.00	1	43	17	2	2	20	42	42	2.00
陈海平	1.00	1	47	15	1	2	21	42	40	24.00
刘洪云	1.00	1	47	12	2	1	20	24	47	288.00
孟连科	1.00	1	52	15	2	1	21	52	52	1.00
王立力	1.00	1	54	6	2	1	20	52	54	25.00
马莉	1.00	2	14	9	1	2	20	14	14	1.00
雷荔	1.00	2	15	9	1	1	20	12	15	40.00
李佳富	1.00	2	16	10	1	2	20	16	16	1.00
李响	1.00	2	16	9	1	2	21	15	16	12.00
赵自辉	1.00	2	16	10	1	2	21	16	16	6.00
卢君	1.00	2	18	12	1	1	21	18	18	.25
秦雪	2.00	2	18	9						
李正文	1.00	2	19	9	1	2	20	17	19	24.00
谢晓霞	1.00	2	19	12	1	2	20	18	19	6.00
张瑗	1.00	2	19	16	1	1	21	19	19	2.00
刘蒙蒙	1.00	2	20	6	1	1	21	16	17	36.00
杨卓越	1.00	2	20	13	1	1	20	18	20	24.00
张静	1.00	2	20	8	1	2	21	18	20	19.00
曹晓	2.00	2	20	9						
冯硕	1.00	2	21	15	1	1	21	21	21	3.00
李菲菲	1.00	2	21	9	1	2	21	19	19	29.00
龙霞	1.00	2	21	9	1	2	20	14	16	30.00
赵洁	1.00	2	21	9	1	2	21	17	21	48.00
赵云轩	1.00	2	21	15	1	1	20	18	21	24.00
王倩	2.00	2	21	15						
黄喆寅	1.00	2	22	12	1	2	20	21	22	15.00
刘海云	1.00	2	22	12	1	2	21	19	20	38.00
刘兴华	1.00	2	22	8	2	2	20	19	22	36.00
陶涛	2.00	2	22	12						
闫昊	2.00	2	22	12						
张宏	2.00	2	22	12						
张艳秋	2.00	2	22	12						
白雪	1.00	2	23	16	1	2	21	16	22	84.00
陈小丽	1.00	2	23	91	0	1	21	23	23	5.00
刘娜	1.00	2	23	9	1	1	20	23	23	1.00
吕琼	1.00	2	23	9	1	2	21	23	23	3.00
孟佳玉	1.00	2	23	16	1	2	20	21	21	21.00
李艳妃	2.00	2	23	9						
刘东方	2.00	2	23	16						
曹顺晰	1.00	2	24	18	1	2	21	24	24	3.00
黄欣娟	1.00	2	24	19	2	2	21	24	24	.83
李素红	1.00	2	24	15	1	2	20	24	24	3.00
刘海波	2.00	2	24	19						
林坤	1.00	2	25	9	1	2	20	24	25	3.00
王菲	1.00	2	25	16	1	1	20	23	25	21.00

吴冬梅	1.00	2	25	15	1	2	21	25	25	.50
张璇	1.00	2	25	16	1	2	21	25	25	1.00
肖艳霞	2.00	2	25	16						
刘琳	1.00	2	26	16	1	1	20	21	26	60.00
郭威	1.00	2	27	16	1	1	21	26	26	18.00
李静	1.00	2	27	16	1	2	21	27	27	1.00
李思宁	1.00	2	27	16	1	2	20	23	26	36.00
刘海霞	1.00	2	27	9	1	2	20	18	18	9.00
齐月娥	1.00	2	27	16	1	2	20	27	27	.10
任翠娥	1.00	2	27	8	1	2	21	25	25	31.00
宋媛	1.00	2	27	15	1	1	20	22	27	60.00
向春来	1.00	2	27	12	2	2	21	27	27	.17
伊雪	1.00	2	27	15	1	2	21	23	24	48.00
李红娟	2.00	2	27	19						
王璇	2.00	2	27	19						
韩敬瑞	1.00	2	28	15	1	1	20	26	28	23.00
于乐	1.00	2	28	16	1	1	20	19	22	42.00
曹珍艳	1.00	2	29	19	2	1	21	28	29	4.00
郭穆	1.00	2	29	15	1	2	21	28	28	17.00
刘畅	1.00	2	29	16	3	2	20	28	29	12.00
王梦娜	1.00	2	29	12	2	2	20	29	29	2.00
张茜	1.00	2	29	19	1	2	20	27	29	19.00
姜雯	1.00	2	30	12	1	1	21	26	30	48.00
相冰娜	1.00	2	30	12	3	2	21	28	30	24.00
易莉	1.00	2	30	9	1	2	20	29	30	8.00
袁华	1.00	2	30	9	2	2	20	29	30	17.00
吴秀京	1.00	2	31	9	2	2	21	31	34	42.00
杨辉	1.00	2	31	16	3	1	20	28	31	36.00
王雪征	1.00	2	32	16	1	2	20	31	32	9.00
曾利红	1.00	2	32	12	2	2	20	25	32	83.00
窦凌云	1.00	2	33	15	2	1	20	29	30	48.00
于东海	1.00	2	33	12	2	2	20	33	33	6.00
才宏	1.00	2	34	8	2	2	20	29	29	59.00
陈亚萍	1.00	2	34	8	2	2	21	32	32	25.00
韩璐	1.00	2	34	16	1	2	20	30	34	48.00
徐坦赛	1.00	2	34	9	2	1	20	29	30	60.00
杨亚荣	1.00	2	34	12	2	2	20	33	33	16.00
陈俊英	1.00	2	35	13	1	2	21	35	35	6.00
周启芝	1.00	2	36	9	2	2	20	36	36	2.00
雷红杰	1.00	2	37	12	2	2	20	34	37	32.00
付昕	1.00	2	38	15	4	2	20	34	34	48.00
蒋雪芹	1.00	2	39	12	1					
孙秀云	1.00	2	40	6	1	2	21	39	40	13.00
杨婉玲	1.00	2	40	15	2	2	21	38	39	30.00
范彩云	1.00	2	41	2	2	2	21	41	41	.40

李燕	1.00	2	41	12	2	2	20	41	41	1.00
盛蕾	1.00	2	41	15	2	2	21	41	41	.20
吴立新	1.00	2	41	15	2	1	21	39	48	24.00
国玉玲	1.00	2	42	9	2	2	20	42	42	30.00
王翠明	1.00	2	43	16	2	1	21	42	43	12.00
张丽云	1.00	2	43	15	3	1	21	42	43	14.00
张世芬	1.00	2	43	9	2	2	20	37	43	60.00
陈鸿娟	2.00	2	43	15						
张柏芹	2.00	2	43	16						
张春雨	2.00	2	43	15						
吴春宏	1.00	2	46	15	1	2	20	43	46	46.00
马丽芳	1.00	2	47	12	2	1	20	47	47	1.00
郑园园	1.00	2	47	12	4	2	20	42	47	60.00
穆丹	1.00	2	49	12	2	2	20	47	49	33.00
张桂华	1.00	2	51	9	2	2	21	51	51	4.00
黄庆明	1.00	2	53	15	2	2	20	52	52	19.00
王千	1.00	2	53	9	2	2	20	53	53	8.00
王俊英	1.00	2	55	9	2	2	20	49	54	72.00
王秀敏	1.00	2	55	5	1	2	20	11	11	.33
于德敏	1.00	2	60	9	2					

course	moking	height	waist	hip	aisthip	weight	bmi	p	n	g
2.00	1	1.75	77	88	.88	56	18.29	21	35	42
9.00	3	1.65	75	80	.94	55	20.20	18	22	38
5.00	1	1.66	76	96	.79	63	22.86	29	49	86
1.00	1	1.66					16.69	31	22	43
1.00	1	1.74	63	83	.76	49	16.18	32	29	61
.50	1	1.76					11.62	22	24	30
7.00	1	1.76					23.57	29	20	39
.10	1	1.64	63	86	.73	46	17.10	35	35	60
3.00	1	1.80					16.67	22	16	26
4.00	1	1.83					19.41	23	13	57
53.00	1	1.79	84	98	.86	76	23.72	35	29	53
31.00	1	1.62	78	87	.90	55	20.96	22	32	44
24.00	1	1.77	88	100	.88	70	22.34	13	37	50
24.00	1	1.70	76	89	.85	56	19.20	16	22	36
2.00	1	1.72					28.06	17	23	43
24.00	1	1.66	85	94	.90	73	26.49	9	28	35
.50	3	1.74	74	92	.80	51	16.85	25	32	61
12.00	3	1.73	70	86	.81	60	19.88	31	35	44
8.00	1	1.80	84	93	.90	81	25.00	11	26	29
27.00	1	1.66					19.96	20	21	33
21.00	3	1.74	67	86	.78	60	19.82	23	28	45
1.00	1	1.82	74	110	.67	74	22.34	24	18	49
36.00	1	1.78	75	91	.82	63	19.88	32	12	70
1.00	1	1.67	74	94	.79	56	20.08	26	18	34
1.00	1	1.64	73	83	.88	64	23.80	26	11	33
40.00	1	1.64	67	86	.78	48	17.85	30	42	55
2.00	1	1.78	102	114	.89	100	31.56	23	29	56
4.00	1	1.85	87	98	.89	73	21.33	12	19	33
60.00	1	1.75					22.86	30	14	33
36.00	1	1.61	69	83	.83	51	19.68	29	29	37
.30	1	1.67	77	85	.91	46	16.31	38	20	60
4.00	1	1.80	88	102	.86	65	20.06	15	11	39
48.00	1	1.76					20.34	33	42	68
21.00	1	1.78	78	97	.80	69	21.78	21	21	46
5.00	3	1.69	89	95	.94	64	22.41	25	17	41
.10	1	1.74	72	94	.77	68	22.46	32	7	39
37.00	1	1.75	89	105	.85	78	25.47	41	28	72
.50	1	1.71	73	89	.82	67	22.91	26	26	43
.20	1	1.64	90	93	.97	64	23.80	21	24	36
72.00	1	1.73	98	102	.96	68	22.72	16	21	36
36.00	3	1.85	86	94	.91	80	23.37	21	27	30
9.00	3	1.72	100	113	.88	87	29.41	19	26	32
2.00	1	1.76					22.92	20	14	33

6.00	3	1.70	76	96	.79	62	21.45	25	11	34
13.00	1	1.78	63	78	.81	52	16.41	42	29	48
1.00	1	1.67	80	88	.91	57	20.44	25	23	53
4.00	1	1.73	74	89	.83	56	18.71	38	9	60
26.00	1	1.75	86	103	.83	75	24.49	22	22	34
22.00	1	1.67					20.08	22	21	31
10.00	1	1.72					20.96	18	24	40
3.00	1	1.80	90	107	.84	88	27.16	26	28	43
3.00	1	1.70	87	93	.94	65	22.49	22	11	36
36.00	1	1.72	66	83	.80	42	14.20	19	21	42
1.00	1	1.74					23.12	26	11	60
1.00	3	1.71	75	88	.85	60	20.52	25	21	28
1.00	3	1.71	70	80	.88	54	18.30	26	20	42
6.00	1	1.74	84	103	.82	63	20.81	20	15	34
.30	1	1.73	100	103	.97	83	27.73	35	17	62
	1	1.77	90	98	.92	110	35.11	40	25	62
1.00	1	1.81					25.95	28	16	31
1.00	3	1.77	78	91	.86	67	21.23	15	16	71
60.00	1	1.76					23.24	28	22	33
24.00	1	1.68	80	91	.88	54	19.13	22	22	35
3.00	1	1.69					20.66	17	10	28
48.00	1	1.70					23.53	29	36	52
8.00	1	1.84					25.40	21	17	38
4.00	1	1.75	91	96	.95	84	27.49	20	12	32
24.00	1	1.73	77	90	.86	50	16.71	35	29	63
.50	1	1.69					20.66	30	11	33
41.00	1	1.68					22.68	42	7	46
60.00	1	1.81					21.37	29	29	55
1.00	1	1.64	88	98	.90	72	26.77	23	11	45
1.00	1	1.75					24.49	16	16	34
36.00	1	1.66	83	89	.93	60	21.77	39	42	61
60.00	1	1.71	86	93	.92	71	24.28	37	13	40
2.00	1	1.73					23.05	27	14	33
3.00	1	1.72					17.58	25	21	60
30.00	1	1.67	68	81	.84	47	16.85	23	13	37

2.00	1	1.74	73	90	.81	70	23.12	31	13	67
20.00	1	1.75					26.45	24	9	28
1.00	3	1.66	69	85	.81	50	18.14	29	15	64
1.00	1	1.77					23.94	26	13	41
25.00	3	1.75	80	100	.80	67	21.88	19	15	37
1.00	1	1.61	74	86	.86	51	19.68	30	24	54
40.00	1	1.57	67	77	.87	48	19.47	26	25	39
1.00	1	1.55	83	96	.86	57	23.73	18	16	31
12.00	1	1.55					17.48	19	19	28
6.00	1	1.55	62	87	.71	45	18.73	11	25	29
.25	1	1.50	58	78	.74	36	16.00	27	12	44
24.00	1	1.67	80	95	.84	57	20.44	34	23	43
6.00	1	1.57					21.10	26	11	32
.20	1	1.61	61	87	.70	46	17.75	32	17	47
12.00	1	1.57	71	87	.82	51	20.69	21	13	39
24.00	1	1.53					21.79	27	19	38
19.00	1	1.70					19.72	24	20	40
3.00	1	1.58	72	90	.80	50	20.03	14	31	34
29.00	1	1.65	67	83	.81	43	15.61	20	30	43
30.00	1	1.60	66	89	.74	55	21.48	22	32	40
48.00	1	1.62	72	89	.81	55	20.96	16	23	30
24.00	1	1.61					23.15	32	10	60
15.00	1	1.52	93	103	.90	70	30.30	27	19	39
38.00	1	1.62					35.82	21	22	47
36.00	1	1.51					26.31	22	12	33
2.00	1	1.62	62	83	.75	43	16.38	25	15	43
5.00	1	1.58	77	89	.87	48	19.23	22	13	35
1.00	1	1.60	74	96	.77	50	19.53	26	22	35
3.00	1	1.62					19.81	20	27	28
21.00	1	1.71	90	98	.92	62	21.20	26	19	36
2.00	1	1.63	83	103	.81	60	22.58	29	26	39
.67	1	1.57	84	100	.84	64	25.96	34	23	38
3.00	1	1.58	70	88	.80	52	20.83	22	13	42
3.00	1	1.68	80	92	.87	58	20.55	33	9	33
21.00	1	1.61	69	84	.82	48	18.52	25	9	38

.50	1	1.50	69	88	.78	48	21.33	23	10	42
1.00	1	1.54	79	89	.89	71	29.94	25	7	39
60.00	1	1.54	77	92	.84	56	23.61	26	14	39
18.00	1	1.62	95	112	.85	88	33.53	20	12	35
1.00	1	1.66	68	88	.77	48	17.42	30	17	30
36.00	1	1.65	67	87	.77	51	18.73	19	15	37
.50	1	1.52	53	74	.72	39	16.88	33	15	37
.10	1	1.71	110	103	1.07	98	33.51	32	7	38
1.00	1	1.60	92	94	.98	62	24.22	20	16	36
12.00	1	1.65					20.94	24	34	49
.17	1	1.57	76	94	.81	58	23.53	29	17	46
48.00	1	1.67					20.80	42	22	81
23.00	1	1.65	83	99	.84	61	22.41	15	21	29
42.00	1	1.73					22.05	15	12	37
4.00	1	1.61	70	90	.78	49	18.90	24	24	35
17.00	1	1.59					19.38	17	20	32
12.00	1	1.72	73	97	.75	59	19.94	22	20	34
2.00	1	1.62					21.15	40	21	39
19.00	1	1.60	65	98	.66	50	19.53	30	12	33
7.00	1	1.67	63	90	.70	47	16.85	26	24	36
24.00	1	1.58	70	92	.76	50	20.03	30	23	48
8.00	1	1.70	104	105	.99	92	31.83	30	20	46
17.00	1	1.68					22.32	35	29	52
42.00	1	1.68	80	93	.86	62	21.97	15	25	43
36.00	1	1.58	76	96	.79	60	24.03	17	19	28
9.00	1	1.67	62	84	.74	46	16.49	23	10	30
83.00	1	1.57					26.78	30	14	39
48.00	1	1.63	70	90	.78	49	18.44	22	11	30
6.00	1	1.72	64	86	.74	52	17.58	25	15	38
59.00	1	1.53					30.33	28	21	40
25.00	1	1.57					24.75	19	24	38
48.00	1	1.60	74	89	.83	48	18.75	22	15	30
60.00	1	1.50	70	86	.81	44	19.56	33	28	46
16.00	1	1.55	62	86	.72	45	18.73	26	21	44
6.00	1	1.66					29.03	31	17	42
2.00	1	1.60					21.88	24	19	36
32.00	1	1.60					21.88	30	25	44
48.00	1	1.66	86	105	.82	72	26.13	27	18	52
13.00	1	1.58	78	88	.89	59	23.63	33	34	50
30.00	1	1.60	70	90	.78	53	20.70	26	14	30
.40	1	1.65					21.67	36	10	31

1.00	1	1.61	86	98	.88	70	27.01	28	15	38
.20	1	1.60					28.52	28	7	24
24.00	1	1.51	65	86	.76	45	19.74	17	21	33
30.00	1	1.74					22.79	29	7	36
12.00	1	1.54					19.82	20	10	38
14.00	1	1.68	70	87	.80	51	18.07	15	16	35
60.00	1	1.61	73	87	.84	52	20.06	26	15	30

46.00	1	1.53					20.93	20	14	27
1.00	1	1.52					23.81	22	22	43
60.00	3	1.50	94	107	.88	68	30.22	30	25	49
33.00	1	1.72					22.31	21	14	31
4.00	1	1.52	85	96	.89	57	24.67	32	19	44
19.00	1	1.60	75	92	.82	53	20.70	19	20	41
8.00	1	1.63					23.34	24	7	55
72.00	1	1.62	78	92	.85	59	22.48	27	11	31
.33	1	1.62	86	100	.86	66	25.15	25	24	44

ss score	cgi	hamd	tmt	SC	hvlt	ss	ds	nab	maze	bvmt
98	5	15	.24	32.00	21.00	12.00	14.00	9.00	2.30	17.00
78	5	13	.26	34.00	18.00	20.00	19.00	3.00	1.39	14.00
164	7	24	.21	27.00	19.00	11.00	14.00	5.00	1.79	20.00
96	6	5								
122	6	27	.22	18.00	16.00	5.00	8.00	5.00	1.79	5.00
76	6	2								
88	6	13								
130	6	37	.26	29.00	24.00	14.00	17.00	11.00	2.48	29.00
64	6	2								
93	6	21								
117	7	17	.23	41.00	15.00	9.00	13.00	7.00	2.08	21.00
98	6	13	.27	52.00	29.00	11.00	22.00	9.00	2.30	20.00
100	5	31	.27	28.00	28.00	19.00	12.00	17.00	2.89	11.00
74	4	25	.26	34.00	25.00	16.00	21.00	18.00	2.94	27.00
83	6	29								
72	4	12	.23	45.00	28.00	14.00	14.00	5.00	1.79	26.00
118	7	10	.22	4.00	15.00	9.00	18.00	4.00	1.61	6.00
110	6	14	.25	56.00	20.00	19.00	23.00	6.00	1.95	18.00
66	4	6	.29	54.00	26.00	16.00	25.00	23.00	3.18	27.00
74	5	21								
96	5	16	.26	23.00	18.00	16.00	17.00	5.00	1.79	10.00
91	6	10	.24	41.00	25.00	18.00	20.00	15.00	2.77	20.00
114	6	66	.23	35.00	20.00	10.00	17.00	6.00	1.95	20.00
78	5	16	.27	54.00	28.00	23.00	26.00	20.00	3.04	32.00
70	4	23	.26	35.00	22.00	7.00	14.00	7.00	2.08	20.00
127	6	13								
108	6	14	.22	18.00	36.00	20.00	26.00	17.00	2.89	30.00
64	5	3								
77	5	3								
95	6	8	.23	25.00	6.00	8.00	11.00	3.00	1.39	8.00
118	7	26	.23	23.00	11.00	10.00	16.00	8.00	2.20	16.00
65	5	12	.28	50.00	23.00	15.00	16.00	21.00	3.09	17.00
			.29	60.00	20.00	18.00	15.00	17.00	2.89	25.00
143	7	15								
88	5	18	.22	23.00	22.00	19.00	19.00	8.00	2.20	28.00
83	5	10	.26	55.00	21.00	13.00	11.00	25.00	3.26	29.00
78	5	8	.23	33.00	30.00	8.00	20.00	13.00	2.64	25.00
			.30	52.00	18.00	15.00	18.00	13.00	2.64	22.00
141	6	6	.22	40.00	27.00	16.00	15.00	7.00	2.08	30.00
95	6	7	.24		14.00	8.00	5.00	2.00	1.10	6.00
81	5	5	.23	34.00	19.00	10.00	20.00	11.00	2.48	16.00
73	5	2								
78	5	9	.26	30.00	18.00	13.00	18.00	6.00	1.95	6.00
77	5	11	.27	45.00	18.00	17.00	25.00	23.00	3.18	12.00
67	5	6								

70	5	10								
			.30	85.00	27.00	23.00	24.00	19.00	3.00	31.00
119	6	24	.22	15.00	17.00	16.00	18.00	1.00	.69	10.00
101	6	21	.26	55.00	28.00	22.00	20.00	24.00	3.22	30.00
107	6	46	.24	51.00	30.00	5.00	18.00	9.00	2.30	27.00
78	5	12	.22	40.00	24.00	15.00	19.00	6.00	1.95	18.00
74	5	6								
82	6	12								
			.29	53.00	31.00	22.00	26.00	20.00	3.04	32.00
			.26	57.00	35.00	15.00	25.00	20.00	3.04	35.00
97	6	16	.27	51.00	26.00	17.00	22.00	20.00	3.04	32.00
69	5	18	.23	26.00	24.00	14.00	14.00	16.00	2.83	14.00
82	6	21	.24	37.00	27.00	19.00	26.00	19.00	3.00	30.00
97	5	37								
			.32	51.00	31.00	17.00	25.00	23.00	3.18	30.00
			.31	70.00	32.00	21.00	26.00	23.00	3.18	35.00
74	4	22	.27	38.00	18.00	14.00	16.00	7.00	2.08	29.00
			.25	39.00	31.00	18.00	24.00	11.00	2.48	27.00
88	5	15	.23	31.00	17.00	12.00	22.00	20.00	3.04	17.00
			.32	66.00	26.00	22.00	24.00	17.00	2.89	30.00
69	5	33	.28	53.00	27.00	18.00	20.00	10.00	2.40	22.00
114	6	26	.25	25.00	18.00	11.00	4.00	3.00	1.39	22.00
127	6	36	.25	50.00	20.00	11.00	24.00	7.00	2.08	32.00
75	5	13								
102	6	55								
83	6	6								
79		6	.22	23.00	18.00	5.00	12.00	5.00	1.79	13.00
55	3	15								
			.30	80.00	34.00	17.00	28.00	25.00	3.26	33.00
117	6	26								
76	5	11								
64	5	11	.24	37.00	28.00	19.00	25.00	7.00	2.08	28.00
127	6	21	.26	42.00	25.00	19.00	21.00	11.00	2.48	21.00
74	6	7								
95	6	13								
113	6	13								
79	6	13	.26	22.00	25.00	16.00	19.00	4.00	1.61	16.00
66	5	14								
142	6	26								
90	6	7	.24	37.00	18.00	10.00	24.00	5.00	1.79	17.00
			.32	61.00	31.00	21.00	24.00	25.00	3.26	33.00
74	6	3								
106	6	20								
			.31	68.00	35.00	20.00	26.00	24.00	3.22	28.00
73	4	9	.25	37.00	33.00	15.00	22.00	8.00	2.20	28.00

111	6	46	.23	35.00	18.00	5.00	13.00	4.00	1.61	11.00
61	4	9								
108	6	55								
80	6	12								
71	6	6	.24	45.00	22.00	8.00	26.00	10.00	2.40	26.00
108	7	51	.24	54.00	26.00	14.00	27.00	10.00	2.40	27.00
90	6	0	.23	39.00	20.00	11.00	8.00	3.00	1.39	29.00
65	6	5	.24	48.00	29.00	18.00	21.00	4.00	1.61	29.00
66	5	5								
65	4	15								
83	5	8	.26	9.00	25.00	8.00	13.00	10.00	2.40	22.00
			.22	62.00	27.00	16.00	27.00	4.00	1.61	24.00
100	6	20	.24	28.00	24.00	17.00	18.00	17.00	2.89	13.00
69	5	15								
96	6	18	.28	53.00	26.00	13.00	15.00	9.00	2.30	30.00
73	4	1	.24	39.00	27.00	13.00	19.00	4.00	1.61	10.00
84	5	10								
84	6	3								
			.25	53.00	23.00	15.00	22.00	8.00	2.20	28.00
79	5	8	.23	38.00	18.00	9.00	27.00	1.00	.69	12.00
93	6	16	.24	28.00	16.00	2.00	12.00	7.00	2.08	9.00
94	6	9	.25	31.00	16.00	13.00	11.00	14.00	2.71	16.00
69	5	27	.24	34.00	20.00	12.00	21.00	3.00	1.39	17.00
102	6	26								
			.25	67.00	28.00	22.00	24.00	14.00	2.71	27.00
85	6	10	.24	36.00	20.00	10.00	16.00	6.00	1.95	26.00
90	6	11								
67	5	17								
			.25	66.00	29.00	18.00	27.00	18.00	2.94	27.00
			.27	52.00	20.00	21.00	21.00	17.00	2.89	26.00
			.25	44.00	23.00	17.00	26.00	5.00	1.79	22.00
			.28	66.00	26.00	17.00	22.00	21.00	3.09	26.00
83	5	25	.26	52.00	33.00	18.00	28.00	9.00	2.30	20.00
70	4	7	.23	35.00	27.00	13.00	14.00	7.00	2.08	14.00
83	6	12	.24	35.00	31.00	7.00	26.00	2.00	1.10	12.00
75	5	1								
81	6	3	.26	67.00	24.00	16.00	19.00	24.00	3.22	30.00
			.23	62.00	20.00	19.00	26.00	11.00	2.48	27.00
			.26	65.00	29.00	15.00	26.00	10.00	2.40	25.00
94	6	9	.25	62.00	24.00	14.00	24.00	8.00	2.20	10.00
95	6	6	.27	59.00	19.00	19.00	17.00	17.00	2.89	24.00
77	5	14	.22	50.00	24.00	19.00	25.00	9.00	2.30	22.00
			.29	67.00	33.00	21.00	28.00	26.00	3.30	32.00
75	6	3	.25	35.00	27.00	7.00	17.00	6.00	1.95	25.00
72	6	7								

75	5	18	.23	33.00	20.00	13.00	18.00	1.00	.69	13.00
71	5	11	.29	69.00	29.00	19.00	25.00	20.00	3.04	19.00
			.28	70.00	29.00	22.00	26.00	16.00	2.83	34.00
79	5	28	.27	51.00	27.00	19.00	25.00	9.00	2.30	28.00
67	6	6	.30	60.00	32.00	17.00	23.00	6.00	1.95	29.00
77	6	7	.28	41.00	29.00	17.00	22.00	18.00	2.94	21.00
71	5	27	.24	34.00	28.00	15.00	23.00	16.00	2.83	26.00
85	6	13	.27	62.00	24.00	19.00	21.00	8.00	2.20	19.00
77	3	10	.23	39.00	21.00	12.00	21.00	5.00	1.79	21.00
72	6	10	.25	35.00	12.00	8.00	15.00	6.00	1.95	7.00
107	7	11								
92	6	14	.27	47.00	28.00	16.00	24.00	4.00	1.61	24.00
145	7	47								
			.26	46.00	30.00	11.00	24.00	14.00	2.71	29.00
			.27	68.00	35.00	21.00	26.00	21.00	3.09	34.00
65	4	2								
64	4	5								
83	6	4	.25	51.00	27.00	17.00	16.00	9.00	2.30	18.00
69	5	7								
76	5	5	.28	52.00	30.00	18.00	20.00	10.00	2.40	29.00
100	7	26								
75	5	8	.27	49.00	26.00	20.00	22.00	19.00	3.00	22.00
86	6	6	.24	27.00	24.00	4.00	17.00	3.00	1.39	17.00
101	6	18	.26	36.00	19.00	13.00	22.00	8.00	2.20	24.00
96	6	14	.25	44.00	15.00	13.00	12.00	4.00	1.61	10.00
116	6	25								
83	6	20								
64	5	5	.29	44.00	33.00	14.00	19.00	10.00	2.40	32.00
63	5	8	.24	48.00	28.00	17.00	22.00	4.00	1.61	31.00
83	6	8								
63	5	14	.24	65.00	29.00	20.00	25.00	10.00	2.40	29.00
78	5	6	.27	50.00	26.00	13.00	15.00	13.00	2.64	19.00
89	6	25								
81	6	6								
67	4	15	.28	52.00	31.00	14.00	24.00	6.00	1.95	29.00
107	6	13	.23	19.00	17.00	7.00	14.00	2.00	1.10	24.00
91	6	12	.24	15.00	14.00	4.00	8.00	2.00	1.10	13.00
90	6	9								
79	6	7								
99	6	10								
97	6	13	.22	42.00	24.00	16.00	14.00	7.00	2.08	28.00
117	7	10	.23	16.00	9.00	7.00	7.00	4.00	1.61	4.00
70	5	13	.24	35.00	27.00	15.00	19.00	7.00	2.08	22.00
77	6	6								

81	6	30	.23	51.00	34.00	17.00	22.00	3.00	1.39	24.00
59	5	7								
71	5	4	.27	47.00	29.00	21.00	19.00	6.00	1.95	28.00
72	6	12								
68	5	32								
66	5	4								
71	6	9	.26	24.00	25.00	27.00	16.00	4.00	1.61	11.00
			.27	50.00	29.00	16.00	25.00	11.00	2.48	25.00
			.27	62.00	24.00	13.00	18.00	11.00	2.48	26.00
			.25	63.00	26.00	12.00	27.00	22.00	3.14	30.00
61	4	11								
87	6	17								
104	7	19	.20	17.00	15.00	11.00	14.00	1.00	.69	8.00
66	5	18								
95	5	13								
80	6	19								
86	6	29								
69	5	12								
93	6	8	.23	8.00	16.00	12.00	12.00	5.00	1.79	17.00
			.26	39.00	26.00	8.00	19.00	2.00	1.10	20.00

cf	eit	cptd3	score	stroop	ep	dspana	score	tmt	sc	hvlt
19.00	4.17	.45	31.00	28.00	3.10	18.0	31.00	43	32	42
16.00	7.70	1.06	35.00	30.00	7.92	15.0	34.00	48	33	35
23.00	5.97	1.02	31.00	27.00	2.10	13.0	29.00	32	27	37
19.00	4.88			12.00	13.13	14.0	25.00	36	18	30
26.00	9.46	1.08	47.00	31.00	11.71	17.0	36.00	50	29	49
20.00	8.62	.20	33.00	20.00	11.80	14.0	31.00	39	40	28
20.00	7.91	2.55	49.00	37.00	9.81	24.0	31.00	54	51	60
28.00	9.31	1.94	48.00	12.00	4.41	14.0	34.00	54	28	58
12.00	8.46	1.22	47.00	15.00	8.93	19.0	34.00	51	33	51
22.00	8.54	1.52	44.00	24.00	12.91	14.0	27.00	38	44	58
11.00	8.84	.29	24.00	16.00	8.08	12.0	21.00	35	4	28
19.00	6.43	1.59	42.00	39.00	10.68	19.0	34.00	45	55	40
19.00	10.71	3.13	60.00	54.00	10.19	25.0	34.00	61	53	53
20.00	3.87	2.16	32.00	45.00	2.87	17.0	27.00	48	23	35
23.00	9.99	1.65	50.00	41.00	2.80	19.0	36.00	41	40	51
24.00	9.30	.79	38.00	34.00	10.99	16.0	32.00	40	34	40
21.00	8.51	2.62	61.00	47.00	11.66	19.0	36.00	52	53	58
16.00	8.35	1.07	37.00	40.00	2.51	15.0	31.00	51	34	44
19.00	6.23	1.48	52.00	41.00	1.53	28.0	40.00	37	18	76
.00	10.35			16.00	12.85	10.0	2.00	39	25	8
17.00	10.74	.57	33.00	20.00	6.87	9.0	27.00	40	23	19
20.00	11.32	1.76	51.00	39.00	11.07	25.0	30.00	56	49	46
23.00	7.03	1.31	47.00	39.00	11.40	13.0	34.00	58	59	40
21.00	8.86	2.84	48.00	39.00	3.67	24.0	34.00	37	23	44
21.00	7.94	1.09	46.00	41.00	8.43	10.0	38.00	51	54	42
31.00	9.72	2.66	53.00	36.00	2.70		8.00	39	36	60
20.00	8.92	2.23	48.00	40.00	8.01	15.0	31.00	63	51	35
35.00	7.74	1.23	46.00	28.00	5.12	17.0	34.00	35	39	56
13.00	8.96	.39		3.00	8.41	15.0	14.00	43		26
16.00	8.91	1.67	39.00	34.00	7.84	22.0	27.00	39	33	37
10.00	6.87	1.73	32.00	49.00	8.35	11.0	25.00	50	30	35
17.00	5.72	1.78	43.00	35.00	9.86	20.0	36.00	53	44	35

35.00	11.15	3.05	69.00	58.00	7.66	26.0		63	83	56
12.00	4.88			10.00	13.30	14.0	27.00	35	15	33
26.00	11.47	2.11	62.00	68.00	6.30	22.0	38.00	50	54	58
26.00	6.99	1.19	44.00	58.00	2.00	20.0	34.00	43	50	62
22.00	9.91			27.00	1.93	22.0	29.00	36	39	49
22.00	9.04	2.79	63.00	41.00	1.85	25.0		58	52	65
28.00	10.63	4.05	68.00	60.00	9.56	22.0		49	56	74
33.00	9.84	1.79	57.00	42.00	10.10	18.0	38.00	51	50	53
19.00	11.22	1.45	44.00	17.00	7.64	17.0	29.00	39	26	49
31.00	11.65	2.86	61.00	47.00	11.18	21.0	38.00	42	36	56
24.00	11.48	3.35	66.00	72.00	10.63	27.0		69	50	65
27.00	10.78	3.39	71.00	69.00	4.74	20.0	34.00	68	68	67
15.00	6.87	.94	38.00	35.00	9.57	17.0	29.00	51	37	35
19.00	10.74	2.40	56.00	48.00	12.52	22.0		47	38	65
25.00	9.12	2.25	45.00	28.00	7.07	20.0	36.00	39	31	33
19.00	11.54	3.63	66.00	54.00	9.93	20.0		69	65	53
22.00	7.75	2.00	50.00	55.00	5.08	17.0	40.00	55	52	56
22.00	6.74	-.17	27.00	17.00	12.65	9.0	15.00	46	25	35
19.00	10.84	2.60	50.00	52.00	8.31	26.0	38.00	45	49	40
17.00	5.80	1.58	27.00	22.00	3.79	17.0	32.00	36	23	35
35.00	9.37	3.58	72.00	67.00	8.00	28.0		64	78	72
23.00	11.08	1.62	55.00	46.00	13.01	20.0	31.00	44	39	57
22.00	6.82	1.44	46.00	24.00	4.85	14.0	36.00	51	41	51
24.00	9.93	1.82	43.00	34.00	12.89	13.0	32.00	51	22	51
26.00	4.88	1.42	34.00	30.00	3.58	21.0	27.00	43	36	35
25.00	8.67	3.52	67.00	51.00	4.08	19.0		69	60	65
31.00	5.90	2.71	63.00	70.00	7.89	24.0		68	67	74
10.00	5.00	1.80	55.00	23.00	11.31	21.0	36.00	50	49	81

25.00	7.67	.71	38.00	29.00	7.85	14.0	34.00	44	47	44
26.00	9.24	2.61	59.00	54.00	8.55	20.0	38.00	48	59	54
19.00	7.10	2.71	53.00	39.00	7.71	26.0	32.00	43	51	54
8.00	6.91	.97	37.00	15.00	2.39	11.0	23.00	41	40	45
27.00	10.00	2.22	54.00	39.00	6.46	24.0	34.00	42	47	59
15.00	9.91	1.72	45.00	22.00	5.48		25.00	49	20	53
12.00	8.85	2.18	50.00	53.00	8.05	19.0	27.00	37	56	56
13.00	4.36	1.25	41.00	20.00	11.73	19.0	29.00	42	33	51
25.00	8.65	1.38	51.00	30.00	8.70	16.0	34.00	57	50	54
36.00	10.12	1.99	47.00	60.00	10.46	15.0	34.00	43	40	56
16.00	8.52	2.73	52.00	64.00	3.88	20.0	34.00	48	50	49
10.00	6.90	2.37	37.00	45.00	12.80	20.0	29.00	41	40	41
22.00	7.14	.13	31.00	30.00	9.15	13.0	15.00	43	33	38
17.00	6.78	.43	38.00	10.00	8.51	11.0	21.00	46	35	38
11.00	8.40	1.56	41.00	40.00	8.19	16.0	25.00	44	37	45
27.00	12.05	3.31	64.00	62.00	9.71	17.0		45	60	57
17.00	6.94	2.12	43.00	30.00	5.56	14.0	27.00	45	38	45
28.00	12.13	3.94	66.00	51.00	10.86	26.0		47	59	59
20.00	9.00	3.01	56.00	41.00	9.18	19.0	34.00	54	49	45
15.00	12.58	2.51	54.00	49.00	10.12	21.0		46	44	49
23.00	9.38	1.63	56.00	34.00	4.27	18.0		58	59	54
24.00	8.06	2.98	57.00	64.00	12.38	21.0	36.00	51	49	65
23.00	7.58	.84	41.00	47.00	2.48	10.0	29.00	40	38	56
7.00	6.10	2.07	39.00	54.00	13.30	22.0	36.00	42	38	62
16.00	8.17	2.33	55.00	51.00	6.42	18.0	34.00	51	60	51
20.00	8.88	1.48	52.00	50.00	11.27	19.0	29.00	41	56	45
24.00	9.95	3.58	60.00	47.00	12.88	27.0		49	58	59
23.00	10.49	2.36	51.00	46.00	11.29	23.0	36.00	47	56	51
25.00	8.02	1.69	51.00	34.00	9.13	13.0	34.00	53	54	43
25.00	8.57	1.92	51.00	50.00	11.48	20.0	32.00	36	48	51
28.00	8.81	3.94	68.00	52.00	12.29	24.0		60	60	65
19.00	7.20	2.30	46.00	42.00	10.16	12.0	25.00	48	38	56

13.00	10.23	1.23	38.00	20.00	6.67	16.0	29.00	40	36	45
27.00	9.54	2.87	60.00	61.00	6.72	25.0	36.00	59	61	59
28.00	10.05	2.48	64.00	46.00	10.82	27.0		57	62	59
32.00	11.46	1.38	58.00	54.00	8.69	20.0	36.00	52	49	56
20.00	7.56	2.14	55.00	51.00	3.63	20.0	38.00	62	55	64
20.00	8.45	2.13	54.00	27.00	8.94	18.0	32.00	56	42	59
23.00	6.68	2.44	52.00	43.00	7.13	20.0	36.00	43	37	57
17.00	10.31	1.80	52.00	38.00	7.28	14.0	32.00	53	56	51
22.00	12.47	1.69	49.00	25.00	10.64	23.0	34.00	41	40	46
18.00	8.42	1.10	35.00	40.00	3.80	16.0	32.00	47	38	32
22.00	10.03	2.00	52.00	41.00	1.36	23.0	34.00	53	46	57
20.00	11.12	2.77	58.00	56.00	10.98	27.0		51	45	60
26.00	12.62	3.19	70.00	57.00	10.96	25.0		54	60	68
26.00	10.48	2.26	52.00	56.00	8.28	15.0	36.00	45	49	56
24.00	6.30			56.00	3.16		.00	58	49	60
24.00	6.80	1.43	52.00	64.00	8.56	19.0	36.00	53	47	54
13.00	5.62	.67	33.00	11.00	4.77	15.0	23.00	42	32	51
15.00	8.65	2.34	48.00	48.00	10.35	22.0	34.00	51	38	43
21.00	8.27	1.19	37.00	38.00	5.18	15.0	30.00	48	44	37
29.00	11.97	2.75	61.00	47.00	9.12	22.0	38.00	60	44	65
26.00	10.48	2.64	56.00	57.00	5.58	22.0	38.00	42	47	57
22.00	9.31	2.96	59.00	50.00	3.05	22.0	40.00	45	58	59
21.00	10.70	.79	49.00	43.00	5.04	12.0	34.00	55	48	54
25.00	12.01	1.69	57.00	45.00	9.89	21.0	40.00	57	49	62
25.00	6.48	.46	34.00	30.00	5.18	14.0	20.00	40	26	40
11.00	4.74	.03	24.00	21.00	11.72	12.0	21.00	44	24	35
21.00	6.87	.84	44.00	44.00	3.65	21.0	34.00	38	42	51
14.00		.20		21.00	10.39	10.0	4.00	45	31	32
28.00	10.02	.80	58.00	33.00	8.90	15.0	38.00	46	47	63

22.00	10.49	3.10	66.00	60.00	7.27	20.0	36.00	42	61	75
24.00	9.59	1.88	66.00	41.00	12.15	21.0	40.00	62	58	67
13.00	8.78	.71	41.00	20.00	12.37	17.0	21.00	59	38	60
18.00	12.12	2.88	71.00	45.00	11.64	25.0		62	60	67
21.00	6.29	2.07	58.00	56.00	12.59	15.0		63	71	58
28.00	12.35	3.48	77.00	47.00	8.07	23.0	38.00	55	72	62
17.00	7.42	.57	33.00	26.00	10.97	13.0	21.00	29	32	43
16.00	5.67			35.00	11.48		13.00	48	30	43
14.00	7.40	1.89	51.00	25.00	4.37	12.0	36.00	61	55	63

ss	e(NAB)	bvmt	cf	eit	cptd	ds
38	48	43	49	23	26	36
59	30	39	44	44	33	46
35	38	47	55	34	33	36
19	38	28	49	27		24
43	51	59	60	54	34	42
30	43	48	51	49	23	34
35	48	47	51	45	52	52
57	59	36	63	53	44	32
48	60	56	38	48	35	50
43	38	55	54	49	39	36
30	34	29	36	51	24	44
57	41	45	49	36	40	54
48	64	56	49	62	59	58
48	38	34	51	21	47	42
54	57	47	55	57	41	48
32	41	47	57	53	30	42
67	62	63	52	49	53	60
24	43	47	44	48	34	36
59	59	60	49	35	39	60
27	30	32	18	59		30
32	46	42	46	62	27	40
46	63	43	51	65	42	40
54	59	54	55	40	37	38
57	46	57	52	51	56	46
40	66	59	52	45	34	30
34	56	51	66	57	55	52
46	54	50	51	51	48	44
48	43	60	75	44	36	38
27	24	29	39	51	25	18
32	51	42	44	51	41	48
40	41	29	34	39	42	44
51	64	37	46	32	42	58

67	61	61	75	64	58	56
48	17	34	38	27		44
65	65	60	60	66	46	48
19	48	56	60	40	35	44
46	41	45	54	57		46
65	62	63	54	52	55	60
46	62	66	63	61	71	58
51	62	63	72	56	42	52
43	58	39	49	65	38	36
57	61	60	68	67	56	60
51	64	60	57	66	62	58
62	64	66	62	62	63	60
43	43	59	42	39	32	40
54	51	56	49	62	50	56
38	62	43	59	52	48	52
65	59	60	49	66	66	56
54	49	50	54	44	45	48
35	30	50	54	38	18	16
35	43	63	49	62	53	56
19	38	38	46	33	40	32
51	66	64	75	54	65	64
62	47	55	55	65	43	62
57	51	48	54	39	38	50
48	34	42	57	57	43	46
32	38	43	60	27	38	56
62	66	64	59	50	64	56
59	65	57	68	33	54	60
52	49	63	34	33	49	60

30	38	41	67	48	34	44
38	56	58	62	59	60	65
49	52	54	49	42	56	66
42	37	57	33	41	35	28
60	40	57	60	59	50	54
34	52	47	43	58	44	38
54	40	50	39	52	49	66
57	60	35	40	27	39	48
47	51	58	57	51	40	42
47	40	31	73	60	47	50
52	49	55	45	50	56	56
37	26	33	36	41	52	66
19	47	29	53	43	26	36
47	57	39	46	41	29	34
44	37	40	37	50	42	54
70	57	54	60	71	63	60
39	45	53	46	41	49	44
60	61	54	62	71	70	66
67	60	53	50	53	59	54
57	43	47	43	74	53	64
57	63	53	55	55	43	56
60	51	44	56	48	59	68
47	47	36	55	45	34	40
31	32	33	32	37	48	64
54	65	58	45	48	51	50
62	53	54	50	53	41	64
52	52	51	56	59	66	64
49	49	31	55	62	52	60
62	60	50	57	48	44	46
62	51	47	57	51	46	62
67	66	61	62	52	70	68
31	45	51	49	43	51	46

47	26	35	40	60	38	48
62	62	43	60	56	57	62
70	59	64	62	59	53	64
62	51	55	67	67	40	62
57	45	57	50	45	49	58
57	61	46	50	50	49	56
52	59	53	55	40	52	58
62	49	43	46	61	45	54
44	43	46	53	73	44	54
34	45	26	47	50	37	42
54	40	50	53	59	47	60
42	57	57	50	65	56	60
67	63	64	59	74	61	64
57	51	42	59	62	50	44
60	52	57	56	38		52
65	61	47	56	41	41	56
24	37	40	40	34	32	46
47	49	50	43	51	51	56
47	40	31	52	49	38	36
49	52	61	63	70	56	50
57	40	59	59	62	55	56
65	52	57	53	55	58	62
47	56	43	52	63	33	42
49	45	57	57	70	44	60
31	32	50	57	39	29	40
24	32	35	37	29	24	28
54	47	55	52	41	34	40
31	45	29	43		31	31
55	53	55	75	64	38	56

61	41	57	61	67	68	62
73	51	63	66	61	52	56
10	45	39	41	55	37	50
58	60	59	52	78	65	68
49	60	60	59	38	55	54
46	71	66	75	80	73	72
43	29	35	50	45	35	46
51	49	50	45	33		42
41	39	54	40	44	53	58

Mode T Sub
90
96
86
78
100
90
90
96
96
82
70
96
96
82
100
92
100
90
108
32
82
88
96
96
104
44
90
96
56
82
78
100

82
104
96
86

104
86
104

96
86

100

108
58
104

92

90
100

92

82

100

96
104
92
74
96
78
82
86
96
96
96
86
58
70
78
82
96
100
86
100
96
86
100
96
92
78

86
100

100
104
92
100
92
96
92

96

100

100
74
96
88

104
104

108
96

108
68
70

96

36
104

100

108

70

104

70

54
100

number	eduyear	gender	marry	date of birth	age	educational st	speak man	nation	gender
1	12	male	remarried	30-Sep-1950	57	high school	Yes	Han	1.00
2	12	male	unmarried	20-Apr-1957	50	high school	Yes	Han	1.00
3	16	female	married	11-Oct-1960	47	Undergraduate	Yes	Han	4.00
4	16	male	divorced	14-Aug-1970	37	Undergraduate	Yes	Han	1.00
5	15	male	divorced	01-Sep-1960	47	junior college	Yes	Han	1.00
6	15	male	unmarried	21-Dec-1972	35	junior college	Yes	Han	1.00
7	12	male	divorced	05-Feb-1958	49	high school	Yes	Han	1.00
8	9	male	unmarried	27-Aug-1966	41	middle school	Yes	Han	1.00
9	9	male	unmarried	06-Sep-1959	48	middle school	Yes	Han	1.00
10	16	female	unmarried	24-Nov-1956	51	Undergraduate	Yes	Han	4.00
11	12	male	unmarried	29-Aug-1968	39	high school	Yes	Han	1.00
12	9	male	married	25-Feb-1947	60	middle school	Yes	Han	1.00
13	9	male	married	04-Feb-1950	57	middle school	Yes	Han	1.00
14	9	male	married	24-Apr-1952	55	middle school	Yes	Han	1.00
15	12	female	unmarried	08-Oct-1987	20	high school	Yes	Han	4.00
16	12	female	divorced	27-Nov-1954	53	high school	Yes	Han	4.00
17	15	female	unmarried	07-Jan-1963	44	junior college	Yes	Han	4.00
18	12	female	unmarried	18-Jan-1961	46	high school	Yes	Han	4.00
19	9	female	married	03-Jun-1951	56	middle school	Yes	Han	4.00
20	16	female	married	25-Jan-1962	45	Undergraduate	Yes	Han	4.00
21	15	female	unmarried	29-Mar-1969	38	junior college	Yes	Han	4.00
22	9	female	married	07-Aug-1952	55	middle school	Yes	Han	4.00
23	15	female	divorced	14-Oct-1961	46	junior college	Yes	Han	4.00
24	12	female	married	23-May-1962	45	high school	Yes	Han	4.00
25	12	female	unmarried	09-May-1951	56	high school	Yes	Han	4.00
26	12	male	unmarried	07-Dec-1958	49	high school	Yes	Han	1.00
27	12	female	unmarried	15-Aug-1971	36	high school	Yes	Han	4.00
28	12	male	unmarried	24-Dec-1958	49	high school	Yes	Han	1.00
29	12	male	married	09-Apr-1960	47	high school	Yes	Han	1.00
30	12	male	unmarried	16-Dec-1970	37	high school	Yes	Han	1.00
31	12	male	unmarried	10-Jul-1965	42	high school	Yes	Han	1.00
32	12	male	unmarried	01-Apr-1965	42	high school	Yes	Han	1.00
33	12	male	unmarried	23-Nov-1960	47	high school	Yes	Han	1.00
34	15	male	married	13-Feb-1958	49	junior college	Yes	Han	1.00
35	12	male	unmarried	17-Apr-1946	61	high school	Yes	Han	1.00
36	12	male	unmarried	21-Aug-1951	56	high school	Yes	Han	1.00
37	9	male	unmarried	16-Dec-1961	46	middle school	Yes	Han	1.00
38	9	male	unmarried	19-Jul-1949	58	middle school	Yes	Han	1.00
39	6	male	divorced	01-Apr-1954	53	primary schoo	Yes	Han	1.00
40	12	male	unmarried	30-May-1947	60	high school	Yes	Han	1.00
41	6	male	unmarried	17-Aug-1960	47	primary schoo	Yes	Han	1.00
42	12	male	unmarried	05-Aug-1961	46	high school	Yes	Han	1.00
43	9	male	divorced	03-Apr-1953	54	middle school	Yes	Han	1.00
44	9	male	unmarried	20-Mar-1955	52	middle school	Yes	Han	1.00
45	12	male	unmarried	26-Jun-1957	50	high school	Yes	Han	1.00
46	9	male	unmarried	24-Oct-1954	53	middle school	Yes	Han	1.00
47	9	male	unmarried	14-Oct-1955	52	middle school	Yes	Han	1.00
48	12	male	divorced	21-Oct-1957	50	high school	Yes	Han	1.00
49	12	male	married	06-Jul-1959	48	high school	Yes	Han	1.00
50	6	female	unmarried	01-Jan-1950	57	primary schoo	Yes	Han	4.00
51	12	female	married	13-Nov-1957	50	high school	Yes	Han	4.00
52	12	male	unmarried	12-Apr-1967	40	high school	Yes	Han	1.00
53	15	male	unmarried	19-Jan-1985	22	junior college	Yes	Han	1.00
54	12	female	unmarried	12-Mar-1959	48	high school	Yes	Han	4.00
55	12	male	divorced	16-May-1964	43	high school	Yes	Han	1.00
56	9	male	married	24-Jul-1964	43	middle school	Yes	Han	1.00
57	9	male	unmarried	05-Apr-1976	31	middle school	Yes	Han	1.00
58	16	female	unmarried	27-Apr-1973	34	Undergraduate	Yes	Han	4.00
59	12	female	unmarried	01-Jul-1952	55	high school	Yes	Han	4.00
60	12	female	married	01-Jul-1959	48	high school	否	Han	4.00
61	12	male	unmarried	25-Apr-1958	49	high school	Yes	Han	1.00

62	12	male	unmarried	13-Jan-1961	46	high school	Yes	Han	1.00
63	16	male	married	01-Feb-1964	43	Undergraduate	Yes	Han	1.00
64	16	male	divorced	20-Jan-1963	44	Undergraduate	Yes	Han	1.00
65	12	male	unmarried	23-Mar-1986	21	high school	Yes	Han	1.00
66	9	male	unmarried	27-Nov-1950	57	middle school	Yes	Han	1.00
67	9	male	divorced	23-Jun-1966	41	middle school	Yes	Han	1.00
68	9	female	unmarried	18-Jan-1950	57	middle school	Yes	Han	4.00
69	9	female	married	05-Aug-1955	52	middle school	Yes	Han	4.00
70	12	female	unmarried	01-Oct-1954	53	high school	Yes	Han	4.00
71	12	female	unmarried	26-Aug-1970	37	high school	Yes	Han	4.00
72	9	female	divorced	27-Feb-1957	50	middle school	Yes	Han	4.00
73	12	female	unmarried	04-Mar-1962	45	high school	Yes	Han	4.00
74	16	female	married	15-Sep-1962	45	Undergraduate	Yes	Han	4.00
75	6	female	unmarried	19-Oct-1966	41	primary schoo	Yes	Han	4.00
76	16	female	unmarried	31-Jan-1963	44	Undergraduate	Yes	Han	4.00
77	12	female	divorced	11-Nov-1955	52	high school	Yes	Han	4.00
78	9	male	unmarried	30-Sep-1952	55	middle school	Yes	Han	1.00
79	12	male	married	31-Jan-1970	37	high school	Yes	Han	1.00
80	12	male	unmarried	23-Mar-1964	43	high school	Yes	Han	1.00
81	9	male	unmarried	07-Aug-1956	51	middle school	Yes	Han	1.00
82	9	male	unmarried	12-May-1961	46	middle school	Yes	Han	1.00
83	9	male	unmarried	28-Apr-1963	44	middle school	Yes	Han	1.00
84	12	male	unmarried	29-May-1961	46	high school	Yes	Han	1.00
85	12	male	divorced	10-Oct-1947	60	high school	Yes	Han	1.00
86	9	female	unmarried	05-Nov-1963	44	middle school	Yes	Han	4.00
87	12	female	unmarried	06-Aug-1963	44	high school	Yes	Han	4.00
88	#NULL!	female	unmarried	09-Jul-1978	29	硕士	Yes	Han	4.00
89	15	female	unmarried	06-Jul-1971	36	junior college	Yes	Han	4.00
90	15	female	divorced	03-Apr-1961	46	junior college	Yes	Han	4.00
91	9	male	married	18-Jul-1950	57	middle school			1.00
92	12	male	unmarried	04-Feb-1949	58	high school			1.00
93	12	male	unmarried	04-May-1981	26	high school			1.00
94	12	male	married	19-Jan-1969	38	high school			1.00
95	12	male	unmarried	25-May-1958	49	high school			1.00
96	#NULL!	male	unmarried	01-Mar-1964	43	硕士			1.00
97	12	male	divorced	23-Mar-1947	60	high school			1.00
98	16	female	unmarried	20-Nov-1985	22	Undergraduate			4.00
99	16	female	unmarried	01-Aug-1982	25	Undergraduate			4.00
100	16	female	unmarried	01-Jun-1948	59	Undergraduate			4.00
101	9	female	unmarried	12-Dec-1963	44	middle school			4.00
102	16	female	unmarried	27-Oct-1964	43	Undergraduate			4.00
103	12	female	unmarried	20-Sep-1960	47	high school			4.00
104	12	female	unmarried	21-Mar-1965	42	high school			4.00
105	9	female	married	07-Jan-1960	47	middle school			4.00
106	9	female	married	15-Nov-1948	59	middle school			4.00
107	12	female	married	20-Jul-1964	43	high school			4.00
108	9	male	unmarried	04-Nov-1955	52	middle school			1.00
109	12	female	unmarried	13-Nov-1970	37	high school			4.00
110	16	female	unmarried	26-Nov-1953	54	Undergraduate			4.00
111	15	female	unmarried	11-May-1971	36	junior college			4.00
112	6	male	unmarried	20-Sep-1954	53	primary schoo			1.00
113	9	male	unmarried	07-Aug-1946	61	middle school			1.00
114	6	male	unmarried	01-Jul-1956	51	primary schoo			1.00
115	9	male	unmarried	19-Nov-1948	59	middle school			1.00
116	9	male	unmarried	23-Aug-1954	53	middle school			1.00
117	9	male	unmarried	21-Jan-1951	56	middle school			1.00
118	9	male	unmarried	14-Mar-1956	51	middle school			1.00
119	12	male	divorced	04-Jun-1954	53	high school			1.00
120	9	male	unmarried	23-Oct-1968	39	middle school			1.00
121	9	female	unmarried	27-Oct-1949	58	middle school			4.00
122	9	female	married	07-Aug-1954	53	middle school			4.00
123	9	female	divorced	01-May-1955	52	middle school			4.00

124	9 female	unmarried	21–Jul–1950	57 middle school	4.00
125	12 female	unmarried	08–Jun–1960	47 high school	4.00
126	12 female	unmarried	09–Dec–1961	46 high school	4.00
127	6 female	married	13–Sep–1953	54 primary schoo	4.00
128	16 female	unmarried	16–Aug–1964	43 Undergraduate	4.00
129	12 female	unmarried	09–Dec–1958	49 high school	4.00
130	9 female	divorced	19–Dec–1963	44 middle school	4.00
131	12 female	unmarried	13–Mar–1973	34 high school	4.00
132	9 female	divorced	03–Dec–1963	44 middle school	4.00
133	16 female	unmarried	31–Jul–1961	46 Undergraduate	4.00
134	12 male	divorced	06–Jun–1957	50 high school	1.00
135	12 male	unmarried	15–Jan–1958	49 high school	1.00
136	12 male	unmarried	01–Jun–1957	50 high school	1.00
137	9 male	unmarried	23–Aug–1961	46 middle school	1.00
138	12 male	unmarried	16–Apr–1959	48 high school	1.00
139	9 male	married	08–Aug–1949	58 middle school	1.00
140	12 male	unmarried	06–Jan–1968	39 high school	1.00
141	9 male	unmarried	23–Jul–1953	54 middle school	1.00
142	9 male	married	16–Mar–1948	59 middle school	1.00
143	9 male	unmarried	01–Aug–1969	38 middle school	1.00
144	9 male	unmarried	10–Jan–1950	57 middle school	1.00
145	12 male	unmarried	01–Jan–1952	55 high school	1.00
146	9 male	divorced	15–Oct–1955	52 middle school	1.00
147	12 male	married	19–Sep–1963	44 high school	1.00
148	9 male	unmarried	25–Dec–1952	55 middle school	1.00
149	12 female	divorced	01–Apr–1963	44 high school	4.00
150	12 female	unmarried	23–Feb–1963	44 high school	4.00
151	12 female	divorced	13–Oct–1952	55 high school	4.00
152	12 female	unmarried	27–Jan–1958	49 high school	4.00
153	12 female	divorced	01–Jan–1957	50 high school	4.00
154	9 female	married	24–Nov–1954	53 middle school	4.00
155	12 female	married	01–Dec–1959	48 high school	4.00
156	15 female	unmarried	21–Nov–1955	52 junior college	4.00
157	12 female	divorced	19–Jan–1958	49 high school	4.00
158	12 female	unmarried	31–Jul–1961	46 high school	4.00
159	12 female	married	05–Dec–1958	49 high school	4.00
160	9 female	unmarried	03–May–1952	55 middle school	4.00
161	12 female	unmarried	31–May–1957	50 high school	4.00
162	15 male	unmarried	01–Sep–1962	45 junior college	1.00
163	12 male	unmarried	22–May–1987	20 high school	1.00
164	12 male	unmarried	28–Feb–1949	58 high school	1.00
165	#NULL! male	married	24–Mar–1967	40 硕士	1.00
166	9 male	unmarried	03–May–1951	56 middle school	1.00
167	16 female	divorced	25–Jun–1961	46 Undergraduate	4.00
168	12 female	unmarried	23–Mar–1959	48 high school	4.00
169	16 female	unmarried	01–Oct–1963	44 Undergraduate	4.00
170	9 female	married	12–Nov–1968	39 middle school	4.00
171	12 female	unmarried	17–Jul–1962	45 high school	4.00
172	9 female	unmarried	15–Jun–1952	55 middle school	4.00
173	16 female	married	25–May–1971	36 Undergraduate	4.00
174	9 male	unmarried	18–Jul–1950	57 middle school	1.00
175	9 male	married	05–May–1967	40 middle school	1.00
176	12 male	unmarried	31–Oct–1962	45 high school	1.00
177	9 male	unmarried	17–Aug–1956	51 middle school	1.00
178	12 male	unmarried	15–Sep–1967	40 high school	1.00
179	9 male	married	12–Sep–1954	53 middle school	1.00
180	12 male	unmarried	23–Jun–1985	22 high school	1.00
181	12 male	unmarried	26–Oct–1979	28 high school	1.00
182	12 male	unmarried	01–Jun–1950	57 high school	1.00
183	9 male	unmarried	01–Jun–1950	57 middle school	1.00
184	9 male	unmarried	20–Mar–1950	57 middle school	1.00
185	12 male	married	08–Feb–1958	49 high school	1.00

186	12	male	unmarried	14-Nov-1968	39	high school	1.00
187	9	male	unmarried	13-Nov-1967	40	middle school	1.00
188	6	male	married	03-Jul-1959	48	primary schoo	1.00
189	15	male	unmarried	07-Jan-1972	35	junior college	1.00
190	12	male	divorced	21-Dec-1957	50	high school	1.00
191	12	male	unmarried	09-Mar-1973	34	high school	1.00
192	12	male	unmarried	01-Jan-1970	37	high school	1.00
193	16	male	unmarried	23-Oct-1965	42	Undergraduate	1.00
194	9	male	divorced	03-Jun-1961	46	middle school	1.00
195	12	male	unmarried	02-Nov-1962	45	high school	1.00
196	6	male	unmarried	03-Jul-1955	52	primary schoo	1.00
197	9	male	unmarried	26-Dec-1947	60	middle school	1.00
198	9	male	unmarried	17-Dec-1951	56	middle school	1.00
199	15	male	unmarried	28-Mar-1971	36	junior college	1.00
200	12	female	divorced	11-Jan-1969	38	high school	4.00
201	12	female	unmarried	28-Nov-1969	38	high school	4.00
202	12	female	married	18-Jul-1957	50	high school	4.00
203	16	female	divorced	26-Jun-1972	35	Undergraduate	4.00
204	15	female	unmarried	22-Oct-1968	39	junior college	4.00
205	9	female	divorced	19-Jan-1954	53	middle school	4.00
206	12	female	divorced	29-Dec-1956	51	high school	4.00
207	12	female	divorced	12-Nov-1960	47	high school	4.00
208	12	female	unmarried	13-May-1973	34	high school	4.00
209	9	female	married	28-Oct-1951	56	middle school	4.00
210	12	female	divorced	12-Oct-1960	47	high school	4.00
211	12	female	married	05-Nov-1962	45	high school	4.00
212	16	female	married	11-Dec-1971	36	Undergraduate	4.00
213	12	female	divorced	11-Aug-1964	43	high school	4.00
214	9	female	divorced	26-Mar-1949	58	middle school	4.00
215	12	female	married	08-Mar-1970	37	high school	4.00
216	9	male	unmarried	26-Jun-1966	41	middle school	1.00
217	9	male	unmarried	05-Oct-1959	48	middle school	1.00
218	9	male	unmarried	04-Jun-1958	49	middle school	1.00
219	9	male	unmarried	04-Jan-1955	52	middle school	1.00
220	9	male	unmarried	02-Jan-1952	55	middle school	1.00
221	12	male	divorced	27-Sep-1960	47	high school	1.00
222	12	male	unmarried	07-Mar-1980	27	high school	1.00
223	9	male	divorced	11-Feb-1952	55	middle school	1.00
224	12	male	unmarried	13-May-1951	56	high school	1.00
225	9	male	divorced	21-Nov-1948	59	middle school	1.00
226	9	male	married	17-Sep-1955	52	middle school	1.00
227	9	male	unmarried	20-Aug-1964	43	middle school	1.00
228	9	male	unmarried	01-Apr-1953	54	middle school	1.00
229	9	male	unmarried	01-Jan-1959	48	middle school	1.00
230	12	male	unmarried	05-Jul-1960	47	high school	1.00
231	9	male	unmarried	01-Feb-1964	43	middle school	1.00
232	12	male	married	12-Dec-1951	56	high school	1.00
233	16	female	married	10-Oct-1968	39	Undergraduate	4.00
234	12	male	unmarried	02-Jul-1969	38	high school	1.00
235	9	male	unmarried	23-Dec-1951	56	middle school	1.00
236	12	male	married	26-Feb-1958	49	high school	1.00
237	9	male	married	28-Nov-1963	44	middle school	1.00
238	9	male	unmarried	29-Nov-1954	53	middle school	1.00
239	9	male	unmarried	20-Jun-1956	51	middle school	1.00
240	9	male	unmarried	25-Feb-1958	49	middle school	1.00
241	16	male	unmarried	26-Nov-1965	42	Undergraduate	1.00
242	16	male	married	07-Mar-1951	56	Undergraduate	1.00
243	12	male	unmarried	05-Jan-1948	59	high school	1.00
244	16	male	unmarried	02-Oct-1952	55	Undergraduate	1.00
245	12	male	unmarried	16-Apr-1959	48	high school	1.00
246	12	male	divorced	10-Feb-1961	46	high school	1.00
247	9	male	divorced	23-Dec-1952	55	middle school	1.00

248	9	male	divorced	26-Jun-1953	54	middle school	1.00
249	9	male	unmarried	06-Jan-1954	53	middle school	1.00
250	12	male	unmarried	11-Nov-1963	44	high school	1.00
251	12	male	unmarried	29-Apr-1987	20	high school	1.00
252	9	male	unmarried	07-Mar-1960	47	middle school	1.00
253	12	male	married	24-Sep-1958	49	high school	1.00
254	9	male	unmarried	26-Jun-1953	54	middle school	1.00
255	12	male	unmarried	16-Apr-1965	42	high school	1.00
256	12	male	unmarried	05-Dec-1950	57	high school	1.00
257	12	male	unmarried	27-Sep-1987	20	high school	1.00
258	12	male	unmarried	28-Feb-1962	45	high school	1.00
259	12	male	divorced	16-Apr-1967	40	high school	1.00
260	12	male	married	07-Jun-1971	36	high school	1.00
261	12	male	divorced	13-Nov-1961	46	high school	1.00
262	12	male	unmarried	26-Oct-1965	42	high school	1.00
263	16	male	unmarried	13-Nov-1967	40	Undergraduate	1.00
264	12	male	divorced	24-Apr-1962	45	high school	1.00
265	12	male	unmarried	07-May-1971	36	high school	1.00
266	9	male	unmarried	29-Apr-1954	53	middle school	1.00
267	12	male	unmarried	26-Dec-1964	43	high school	1.00
268	12	male	unmarried	25-Sep-1962	45	high school	1.00
269	9	male	divorced	30-Apr-1968	39	middle school	1.00
270	9	male	married	27-Oct-1951	56	middle school	1.00

wcstb1	wcsti1	ep1	upsa1	TMT1	SC1	HVLT1	SS1	DS1	NAB1	maze1
1	31	#NULL!	#NULL!	.25	40.00	18.00	7.00	20.00	7.00	2.08
#NULL!	#NULL!	#NULL!	#NULL!	.23	30.00	13.00	12.00	18.00	10.00	2.40
1	39	#NULL!	#NULL!	.25	40.00	21.00	10.00	13.00	3.00	1.39
0	60	#NULL!	#NULL!	.22	40.00	16.00	19.00	25.00	7.00	2.08
2	30	#NULL!	#NULL!	.25	46.00	22.00	12.00	22.00	11.00	2.48
2	25	#NULL!	#NULL!	.23	34.00	20.00	19.00	22.00	5.00	1.79
2	48	#NULL!	#NULL!	.23	17.00	13.00	16.00	8.00	2.00	1.10
1	74	#NULL!	#NULL!	.22	26.00	17.00	10.00	17.00	7.00	2.08
0	22	#NULL!	#NULL!	.23	21.00	17.00	.00	16.00	2.00	1.10
0	56	#NULL!	#NULL!	.26	41.00	26.00	15.00	19.00	6.00	1.95
6	4	#NULL!	#NULL!	.25	37.00	24.00	6.00	22.00	5.00	1.79
1	41	#NULL!	#NULL!	.22	19.00	7.00	10.00	15.00	1.00	.69
1	38	#NULL!	#NULL!	.25	32.00	16.00	12.00	16.00	6.00	1.95
0	10	#NULL!	#NULL!	.21	29.00	10.00	11.00	21.00	5.00	1.79
6	20	#NULL!	#NULL!	.24	44.00	19.00	11.00	16.00	5.00	1.79
0	44	#NULL!	#NULL!	.24	34.00	19.00	7.00	5.00	4.00	1.61
0	58	#NULL!	#NULL!	.25	57.00	24.00	13.00	14.00	5.00	1.79
6	25	#NULL!	#NULL!	.22	33.00	21.00	16.00	14.00	4.00	1.61
0	16	#NULL!	#NULL!	.23	26.00	18.00	9.00	17.00	3.00	1.39
2	26	#NULL!	#NULL!	.26	45.00	29.00	13.00	17.00	20.00	3.04
0	21	#NULL!	#NULL!	.22	45.00	18.00	12.00	15.00	3.00	1.39
1	12	#NULL!	#NULL!	.25	35.00	6.00	13.00	13.00	3.00	1.39
4	25	#NULL!	#NULL!	.25	54.00	22.00	11.00	17.00	2.00	1.10
1	36	#NULL!	#NULL!	.26	36.00	21.00	9.00	14.00	3.00	1.39
3	43	#NULL!	#NULL!	.20	24.00	21.00	11.00	15.00	.00	.00
0	28	#NULL!	#NULL!	.20	15.00	17.00	8.00	6.00	4.00	1.61
#NULL!	#NULL!	#NULL!	#NULL!	.23	37.00	20.00	11.00	18.00	1.00	.69
1	28	#NULL!	#NULL!	.23	28.00	21.00	18.00	16.00	2.00	1.10
1	39	#NULL!	#NULL!	.24	25.00	22.00	13.00	11.00	8.00	2.20
6	5	#NULL!	#NULL!	.30	71.00	31.00	22.00	28.00	18.00	2.94
3	26	#NULL!	#NULL!	.22	31.00	18.00	9.00	17.00	1.00	.69
6	17	#NULL!	#NULL!	.24	35.00	25.00	24.00	17.00	5.00	1.79
0	17	#NULL!	#NULL!	.23	30.00	20.00	10.00	8.00	2.00	1.10
2	43	#NULL!	#NULL!	.28	38.00	16.00	13.00	20.00	4.00	1.61
6	9	#NULL!	#NULL!	.23	39.00	20.00	12.00	19.00	8.00	2.20
0	39	#NULL!	#NULL!	.22	30.00	20.00	16.00	16.00	4.00	1.61
#NULL!	#NULL!	#NULL!	#NULL!	.22	24.00	17.00	10.00	23.00	6.00	1.95
1	34	#NULL!	#NULL!	.20	21.00	15.00	12.00	4.00	2.00	1.10
1	39	#NULL!	#NULL!	.23	15.00	17.00	10.00	16.00	9.00	2.30
0	28	#NULL!	#NULL!	.21	27.00	27.00	10.00	16.00	2.00	1.10
1	58	#NULL!	#NULL!	.21	20.00	2.00	3.00	7.00	2.00	1.10
#NULL!	#NULL!	#NULL!	#NULL!	.20	17.00	15.00	4.00	6.00	3.00	1.39
0	24	#NULL!	#NULL!	.22	12.00	12.00	7.00	15.00	1.00	.69
2	21	#NULL!	#NULL!	.22	26.00	19.00	16.00	3.00	6.00	1.95
0	38	#NULL!	#NULL!	.24	21.00	6.00	12.00	14.00	.00	.00
6	10	#NULL!	#NULL!	.23	20.00	16.00	21.00	25.00	6.00	1.95
0	43	#NULL!	#NULL!	.23	15.00	8.00	8.00	8.00	.00	.00
2	25	#NULL!	#NULL!	.23	22.00	18.00	17.00	20.00	6.00	1.95
6	5	#NULL!	#NULL!	.19	15.00	19.00	12.00	19.00	1.00	.69
#NULL!	#NULL!	#NULL!	#NULL!	.21	12.00	21.00	6.00	3.00	1.00	.69
0	31	#NULL!	#NULL!	.21	28.00	6.00	7.00	12.00	2.00	1.10
1	21	#NULL!	#NULL!	.25	32.00	22.00	13.00	18.00	4.00	1.61
1	48	#NULL!	#NULL!	.26	43.00	23.00	17.00	17.00	16.00	2.83
1	30	#NULL!	#NULL!	.20	22.00	1.00	10.00	10.00	.00	.00
3	51	#NULL!	#NULL!	.25	38.00	22.00	16.00	15.00	11.00	2.48
0	6	#NULL!	#NULL!	.22	48.00	20.00	4.00	16.00	5.00	1.79
2	33	#NULL!	#NULL!	.23	37.00	20.00	8.00	24.00	5.00	1.79
5	26	#NULL!	#NULL!	.29	60.00	22.00	20.00	25.00	7.00	2.08
1	38	#NULL!	#NULL!	.24	26.00	19.00	15.00	16.00	7.00	2.08
#NULL!	#NULL!	#NULL!	#NULL!	.21	30.00	15.00	7.00	15.00	1.00	.69
6	8	#NULL!	#NULL!	.28	41.00	21.00	20.00	26.00	5.00	1.79

6	7	#NULL!	#NULL!	.28	44.00	24.00	14.00	23.00	15.00	2.77
5	15	#NULL!	#NULL!	.27	49.00	21.00	17.00	23.00	3.00	1.39
3	31	#NULL!	#NULL!	.24	37.00	20.00	.00	23.00	3.00	1.39
6	8	#NULL!	#NULL!	.25	46.00	24.00	17.00	20.00	15.00	2.77
1	37	#NULL!	#NULL!	.23	20.00	14.00	6.00	4.00	1.00	.69
#NULL!	#NULL!	#NULL!	#NULL!	.22	19.00	13.00	2.00	12.00	1.00	.69
2	29	#NULL!	#NULL!	.22	15.00	18.00	5.00	16.00	15.00	2.77
4	35	#NULL!	#NULL!	.23	28.00	18.00	11.00	15.00	1.00	.69
0	60	#NULL!	#NULL!	.21	17.00	15.00	6.00	11.00	2.00	1.10
5	25	#NULL!	#NULL!	.25	52.00	28.00	7.00	25.00	5.00	1.79
#NULL!	#NULL!	#NULL!	#NULL!	.24	28.00	20.00	12.00	18.00	1.00	.69
0	82	#NULL!	#NULL!	.23	37.00	14.00	5.00	18.00	2.00	1.10
#NULL!	#NULL!	#NULL!	#NULL!	.26	50.00	23.00	2.00	16.00	1.00	.69
#NULL!	#NULL!	#NULL!	#NULL!	.28	35.00	17.00	13.00	21.00	5.00	1.79
2	20	#NULL!	#NULL!	.29	49.00	18.00	5.00	20.00	8.00	2.20
4	37	#NULL!	#NULL!	.26	42.00	23.00	11.00	16.00	10.00	2.40
0	20	#NULL!	#NULL!	.22	16.00	16.00	10.00	11.00	5.00	1.79
1	49	#NULL!	#NULL!	.27	32.00	14.00	10.00	4.00	4.00	1.61
4	19	#NULL!	#NULL!	.29	39.00	17.00	16.00	18.00	12.00	2.56
1	41	#NULL!	#NULL!	.23	16.00	15.00	5.00	14.00	1.00	.69
#NULL!	#NULL!	#NULL!	#NULL!	.24	20.00	19.00	8.00	6.00	5.00	1.79
#NULL!	#NULL!	#NULL!	#NULL!	.22	11.00	9.00	6.00	4.00	5.00	1.79
#NULL!	#NULL!	#NULL!	#NULL!	.23	24.00	10.00	6.00	3.00	1.00	.69
0	95	#NULL!	#NULL!	.24	23.00	12.00	8.00	8.00	2.00	1.10
0	31	#NULL!	#NULL!	.23	33.00	14.00	7.00	15.00	6.00	1.95
0	92	#NULL!	#NULL!	.23	37.00	18.00	18.00	20.00	3.00	1.39
6	4	#NULL!	#NULL!	.27	40.00	29.00	18.00	27.00	7.00	2.08
1	44	#NULL!	#NULL!	.29	44.00	17.00	16.00	18.00	5.00	1.79
2	28	#NULL!	#NULL!	.23	34.00	25.00	18.00	25.00	7.00	2.08
1	29	10.002	14	.23	23.00	12.00	11.00	14.00	5.00	1.79
2	50	11.192	15	.22	27.00	14.00	13.00	18.00	2.00	1.10
4	24	10.317	23	.26	34.00	17.00	11.00	13.00	7.00	2.08
0	19	12.047	25	.24	38.00	27.00	18.00	18.00	4.00	1.61
0	36	13.300	21	.20	20.00	21.00	7.00	14.00	2.00	1.10
1	45	8.894	36	.22	24.00	24.00	12.00	16.00	5.00	1.79
1	86	12.549	13	.19	19.00	11.00	2.00	15.00	1.00	.69
1	34	6.441	31	.30	59.00	13.00	18.00	22.00	17.00	2.89
0	82	11.942	36	.25	46.00	20.00	10.00	19.00	2.00	1.10
1	32	12.632	25	.20	34.00	14.00	13.00	13.00	2.00	1.10
6	15	12.889	25	.42	27.00	23.00	13.00	22.00	5.00	1.79
6	15	10.560	38	.25	50.00	27.00	17.00	24.00	17.00	2.89
4	27	11.289	30	.25	40.00	16.00	14.00	14.00	13.00	2.64
5	15	11.901	25	.22	32.00	26.00	18.00	20.00	3.00	1.39
1	38	4.217	23	.22	28.00	17.00	7.00	14.00	5.00	1.79
1	47	13.055	27	.19	19.00	16.00	8.00	12.00	1.00	.69
3	24	5.519	36	.22	35.00	22.00	18.00	19.00	8.00	2.20
2	55	4.627	14	.24	29.00	16.00	12.00	16.00	6.00	1.95
6	9	8.822	36	.26	41.00	17.00	14.00	17.00	7.00	2.08
0	30	12.852	6	.22	23.00	5.00	4.00	7.00	2.00	1.10
0	91	5.132	23	.26	37.00	14.00	15.00	12.00	3.00	1.39
0	85	10.615	19	.23	17.00	16.00	5.00	17.00	2.00	1.10
0	72	11.179	16	.21	16.00	15.00	16.00	8.00	1.00	.69
1	41	12.450	12	.23	36.00	14.00	9.00	10.00	4.00	1.61
1	15	12.852	14	.62	21.00	6.00	6.00	8.00	3.00	1.39
1	49	13.043	32	.23	20.00	16.00	11.00	16.00	2.00	1.10
1	34	10.553	20	.23	33.00	17.00	14.00	15.00	2.00	1.10
4	26	13.300	18	.25	38.00	18.00	15.00	18.00	5.00	1.79
1	18	6.918	12	.20	13.00	11.00	7.00	10.00	2.00	1.10
1	34	13.300	28	.25	36.00	17.00	13.00	10.00	4.00	1.61
2	33	9.089	15	.24	27.00	12.00	14.00	12.00	1.00	.69
2	31	12.558	32	.22	32.00	21.00	14.00	19.00	7.00	2.08
1	49	13.300	23	.21	46.00	17.00	11.00	15.00	3.00	1.39

2	39	12.910	25	.25	50.00	20.00	14.00	12.00	4.00	1.61
1	51	13.300	18	.21	42.00	17.00	16.00	14.00	2.00	1.10
0	8	10.134	29	.21	35.00	22.00	16.00	15.00	3.00	1.39
2	43	12.483	17	.23	13.00	16.00	7.00	11.00	3.00	1.39
3	19	4.879	21	.25	32.00	16.00	8.00	13.00	2.00	1.10
0	39	12.421	27	.24	50.00	19.00	19.00	17.00	3.00	1.39
0	23	12.069	29	.21	29.00	26.00	11.00	25.00	5.00	1.79
1	27	12.860	21	.20	29.00	11.00	14.00	22.00	3.00	1.39
1	28	13.300	29	.26	46.00	19.00	10.00	21.00	4.00	1.61
5	21	13.105	25	.24	35.00	14.00	10.00	17.00	1.00	.69
2	28	9.530	27	.23	12.00	16.00	10.00	16.00	2.00	1.10
0	90	13.134	8	.19	14.00	8.00	8.00	7.00	1.00	.69
0	75	8.715	2	.19	23.00	17.00	9.00	15.00	3.00	1.39
1	30	10.173	12	.22	39.00	20.00	10.00	16.00	1.00	.69
1	20	11.755	23	.25	41.00	14.00	4.00	13.00	2.00	1.10
0	17	10.313	12	.20	17.00	15.00	3.00	10.00	1.00	.69
0	17	11.715	36	.24	35.00	16.00	14.00	14.00	2.00	1.10
1	70	12.993	27	.18	21.00	14.00	15.00	13.00	1.00	.69
1	20	11.388	23	.22	24.00	15.00	11.00	13.00	2.00	1.10
1	51	9.246	24	.20	20.00	26.00	15.00	16.00	1.00	.69
0	21	12.457	6	.22	19.00	12.00	2.00	14.00	10.00	2.40
0	17	11.178	6	.21	15.00	13.00	1.00	11.00	6.00	1.95
0	27	7.594	0	.18	4.00	10.00	2.00	3.00	.00	.00
3	40	1.401	14	.25	29.00	17.00	10.00	19.00	11.00	2.48
1	33	10.021	23	.22	33.00	13.00	10.00	17.00	6.00	1.95
4	35	9.063	6	.24	32.00	18.00	12.00	15.00	3.00	1.39
1	39	13.250	27	.21	33.00	19.00	9.00	10.00	2.00	1.10
1	19	12.860	38	.24	37.00	16.00	12.00	14.00	9.00	2.30
3	38	13.300	33	.22	20.00	23.00	15.00	18.00	4.00	1.61
1	39	10.724	25	.20	28.00	15.00	6.00	11.00	1.00	.69
0	37	9.147	15	.21	31.00	19.00	11.00	15.00	1.00	.69
3	18	11.047	25	.22	39.00	25.00	16.00	25.00	3.00	1.39
0	92	12.782	25	.25	42.00	22.00	11.00	15.00	3.00	1.39
0	51	10.814	8	.23	19.00	11.00	1.00	13.00	2.00	1.10
0	95	12.852	10	.21	23.00	11.00	6.00	3.00	1.00	.69
1	33	8.914	6	.19	13.00	12.00	8.00	11.00	1.00	.69
1	33	11.710	17	.27	40.00	11.00	14.00	8.00	4.00	1.61
0	44	5.465	29	.22	27.00	12.00	12.00	15.00	1.00	.69
5	31	10.615	38	.23	48.00	24.00	19.00	26.00	4.00	1.61
1	42	11.009	27	.25	42.00	21.00	18.00	19.00	9.00	2.30
1	51	12.125	19	.23	32.00	14.00	14.00	14.00	4.00	1.61
6	5	5.542	34	.31	62.00	27.00	12.00	25.00	21.00	3.09
1	64	11.494	27	.20	22.00	4.00	5.00	9.00	2.00	1.10
1	24	11.175	36	.23	46.00	27.00	16.00	17.00	3.00	1.39
0	67	12.523	19	.23	29.00	11.00	10.00	5.00	2.00	1.10
0	94	11.924	27	.23	35.00	20.00	15.00	15.00	2.00	1.10
0	14	11.566	32	.22	28.00	10.00	12.00	16.00	2.00	1.10
1	21	11.561	25	.24	35.00	15.00	13.00	17.00	8.00	2.20
1	86	8.462	17	.24	25.00	12.00	10.00	7.00	5.00	1.79
0	63	11.765	31	.24	55.00	23.00	13.00	13.00	3.00	1.39
0	18	4.600	23	.22	18.00	22.00	16.00	11.00	5.00	1.79
0	27	4.395	15	.25	29.00	13.00	6.00	13.00	17.00	2.89
0	18	4.023	26	.23	31.00	27.00	15.00	16.00	4.00	1.61
1	43	10.440	23	.23	37.00	16.00	10.00	11.00	6.00	1.95
0	61	5.830	36	.30	51.00	25.00	18.00	17.00	7.00	2.08
0	89	11.955	25	.20	16.00	22.00	17.00	15.00	3.00	1.39
1	40	6.027	12	.24	47.00	21.00	17.00	23.00	14.00	2.71
1	36	12.939	20	.23	26.00	21.00	14.00	14.00	18.00	2.94
4	41	13.043	15	.25	29.00	12.00	12.00	18.00	4.00	1.61
1	24	11.940	2	.22	25.00	13.00	13.00	8.00	7.00	2.08
1	31	10.740	31	.26	32.00	28.00	12.00	18.00	15.00	2.77
0	21	12.599	19	.22	20.00	11.00	13.00	10.00	8.00	2.20

6	9	6.062	36	.22	38.00	31.00	19.00	25.00	9.00	2.30
6	23	13.300	17	.20	28.00	14.00	12.00	5.00	2.00	1.10
0	17	9.035	11	.19	18.00	12.00	12.00	8.00	1.00	.69
6	18	10.491	36	.22	34.00	32.00	14.00	26.00	14.00	2.71
6	30	3.374	23	.22	19.00	17.00	12.00	19.00	8.00	2.20
1	54	8.716	25	.27	54.00	31.00	18.00	23.00	11.00	2.48
0	91	8.677	25	.21	38.00	21.00	7.00	19.00	3.00	1.39
1	38	12.602	31	.28	50.00	25.00	17.00	18.00	14.00	2.71
1	49	10.466	25	.23	35.00	15.00	11.00	17.00	6.00	1.95
0	18	12.384	23	.26	20.00	12.00	10.00	11.00	5.00	1.79
6	14	6.233	25	.26	32.00	27.00	17.00	24.00	19.00	3.00
1	35	5.520	23	.28	30.00	22.00	12.00	13.00	16.00	2.83
2	62	6.781	14	.21	27.00	17.00	8.00	17.00	2.00	1.10
6	16	10.789	29	.26	65.00	30.00	16.00	23.00	20.00	3.04
4	23	4.896	36	.25	53.00	25.00	9.00	24.00	11.00	2.48
2	42	13.043	38	.25	45.00	20.00	17.00	18.00	2.00	1.10
2	29	10.781	33	.23	24.00	17.00	7.00	18.00	2.00	1.10
1	27	10.818	34	.24	43.00	27.00	16.00	22.00	3.00	1.39
4	21	12.457	40	.24	56.00	30.00	16.00	25.00	5.00	1.79
1	56	11.652	8	.26	19.00	18.00	2.00	9.00	4.00	1.61
0	29	6.408	36	.25	35.00	22.00	16.00	18.00	13.00	2.64
1	45	8.710	21	.26	28.00	21.00	8.00	11.00	1.00	.69
0	15	8.178	19	.20	24.00	14.00	6.00	9.00	3.00	1.39
0	80	8.013	2	.21	35.00	9.00	5.00	11.00	4.00	1.61
0	73	7.067	10	.21	31.00	16.00	5.00	11.00	5.00	1.79
2	39	11.504	34	.23	50.00	23.00	15.00	22.00	14.00	2.71
5	12	8.388	38	.24	50.00	22.00	16.00	25.00	11.00	2.48
1	20	10.699	21	.23	44.00	17.00	6.00	11.00	2.00	1.10
3	25	11.998	38	.24	22.00	21.00	13.00	15.00	5.00	1.79
6	3	12.798	36	.31	53.00	31.00	20.00	25.00	9.00	2.30
6	14	13.134	14	.23	35.00	17.00	8.00	14.00	2.00	1.10
1	36	11.125	30	.23	24.00	20.00	19.00	16.00	19.00	3.00
0	32	8.902	25	.23	27.00	17.00	9.00	14.00	1.00	.69
4	24	12.416	18	.22	14.00	10.00	8.00	13.00	2.00	1.10
0	20	5.676	14	.20	22.00	15.00	11.00	14.00	6.00	1.95
1	66	12.852	18	.24	31.00	15.00	4.00	11.00	11.00	2.48
6	18	7.886	27	.28	56.00	19.00	12.00	25.00	14.00	2.71
4	41	2.072	27	.25	50.00	16.00	19.00	24.00	10.00	2.40
0	22	12.026	27	.21	49.00	18.00	11.00	23.00	4.00	1.61
1	53	13.043	19	.23	24.00	11.00	14.00	8.00	3.00	1.39
0	22	9.549	19	.20	18.00	11.00	11.00	4.00	1.00	.69
1	44	7.300	17	.24	28.00	31.00	15.00	17.00	9.00	2.30
2	33	12.104	25	.23	35.00	19.00	17.00	21.00	11.00	2.48
2	54	13.250	15	.20	19.00	11.00	8.00	9.00	7.00	2.08
0	15	10.935	16	.19	5.00	10.00	.00	4.00	2.00	1.10
1	65	13.134	23	.22	23.00	25.00	18.00	19.00	7.00	2.08
1	64	12.889	25	.22	31.00	14.00	12.00	17.00	4.00	1.61
4	20	10.970	36	.23	41.00	18.00	13.00	15.00	3.00	1.39
0	33	13.300	29	.23	33.00	28.00	15.00	20.00	11.00	2.48
1	32	10.511	29	.26	38.00	12.00	1.00	13.00	9.00	2.30
2	37	13.300	25	.26	31.00	16.00	12.00	16.00	10.00	2.40
4	30	9.927	25	.25	23.00	18.00	11.00	11.00	4.00	1.61
1	38	10.431	34	.25	38.00	20.00	15.00	18.00	11.00	2.48
2	36	11.972	23	.20	29.00	30.00	15.00	18.00	5.00	1.79
1	23	8.730	21	.22	9.00	11.00	11.00	10.00	4.00	1.61
6	30	10.922	34	.27	45.00	26.00	18.00	22.00	7.00	2.08
4	32	12.615	31	.25	37.00	20.00	14.00	24.00	6.00	1.95
0	71	8.035	17	.25	18.00	10.00	13.00	9.00	4.00	1.61
3	28	11.304	25	.24	23.00	19.00	16.00	15.00	14.00	2.71
0	35	12.051	14	.24	24.00	20.00	10.00	15.00	1.00	.69
0	88	12.860	19	.23	20.00	8.00	11.00	10.00	7.00	2.08
1	44	10.637	8	.21	16.00	16.00	5.00	11.00	4.00	1.61

1	66	11.467	23	.24	34.00	18.00	11.00	11.00	9.00	2.30
0	73	11.378	8	.24	20.00	13.00	11.00	16.00	2.00	1.10
1	34	12.910	23	.21	30.00	15.00	10.00	10.00	4.00	1.61
2	42	11.379	21	.27	48.00	19.00	12.00	12.00	10.00	2.40
6	14	7.511	29	.25	47.00	26.00	13.00	16.00	6.00	1.95
2	31	11.354	18	.26	37.00	19.00	11.00	14.00	2.00	1.10
1	19	13.010	25	.22	28.00	16.00	16.00	17.00	13.00	2.64
0	29	5.764	19	.23	27.00	15.00	9.00	14.00	3.00	1.39
2	39	10.644	25	.25	27.00	22.00	13.00	19.00	2.00	1.10
6	4	10.983	25	.27	44.00	26.00	15.00	25.00	11.00	2.48
1	26	10.046	23	.28	36.00	22.00	13.00	16.00	5.00	1.79
5	30	7.806	34	.22	23.00	20.00	12.00	20.00	5.00	1.79
6	8	11.863	34	.25	35.00	16.00	16.00	21.00	13.00	2.64
1	29	12.491	25	.25	36.00	22.00	13.00	14.00	19.00	3.00
4	40	11.786	27	.22	31.00	20.00	13.00	18.00	6.00	1.95
1	40	8.750	27	.27	59.00	26.00	21.00	23.00	9.00	2.30
0	18	10.049	19	.23	18.00	12.00	4.00	16.00	4.00	1.61
2	46	3.881	33	.30	52.00	26.00	16.00	25.00	23.00	3.18
1	28	13.084	30	.23	29.00	20.00	8.00	14.00	1.00	.69
0	53	13.043	27	.27	43.00	26.00	17.00	21.00	15.00	2.77
2	31	10.627	29	.26	36.00	26.00	17.00	13.00	9.00	2.30
5	17	12.939	31	.24	34.00	22.00	16.00	18.00	19.00	3.00
0	10	9.992	4	.19	12.00	12.00	3.00	9.00	1.00	.69

BVMT1	CF1	EIT1	cptd1	stroop1	phantom hearing	Grouping	smoking	oking time	of smoking	of disease
16.00	30.00	7.36	#NULL!	39.00	#NULL!	2	2.00	36.00	20.00	35.00
19.00	18.00	7.42	#NULL!	40.00	#NULL!	2	2.00	15.00	10.00	26.00
28.00	15.00	7.50	#NULL!	66.00	4	1	1.00	#NULL!	#NULL!	27.00
22.00	20.00	11.32	#NULL!	60.00	#NULL!	2	2.00	15.00	40.00	14.00
22.00	20.00	7.63	#NULL!	40.00	#NULL!	2	2.00	20.00	20.00	22.00
15.00	12.00	8.09	#NULL!	11.00	#NULL!	2	1.00	#NULL!	#NULL!	17.00
8.00	12.00	5.34	#NULL!	20.00	#NULL!	2	2.00	28.00	20.00	28.00
10.00	3.00	8.47	#NULL!	18.00	#NULL!	2	1.00	#NULL!	#NULL!	22.00
10.00	21.00	7.75	#NULL!	28.00	#NULL!	2	2.00	25.00	20.00	27.00
7.00	19.00	6.77	#NULL!	35.00	#NULL!	2	1.00	#NULL!	#NULL!	25.00
16.00	23.00	7.58	#NULL!	34.00	#NULL!	2	1.00	#NULL!	#NULL!	9.00
14.00	20.00	8.87	#NULL!	34.00	#NULL!	2	1.00	#NULL!	#NULL!	36.00
7.00	24.00	8.49	#NULL!	32.00	#NULL!	2	#NULL!	#NULL!	#NULL!	29.00
17.00	18.00	8.98	#NULL!	41.00	#NULL!	2	2.00	20.00	10.00	29.00
11.00	17.00	10.74	#NULL!	38.00	#NULL!	2	2.00	4.00	10.00	3.00
16.00	15.00	8.29	#NULL!	41.00	#NULL!	2	1.00	#NULL!	#NULL!	36.00
15.00	17.00	8.36	#NULL!	49.00	#NULL!	2	1.00	#NULL!	#NULL!	14.00
20.00	14.00	6.06	#NULL!	27.00	#NULL!	2	1.00	#NULL!	#NULL!	16.00
13.00	16.00	8.01	#NULL!	25.00	5	1	1.00	#NULL!	#NULL!	31.00
29.00	16.00	8.11	#NULL!	28.00	#NULL!	2	1.00	#NULL!	#NULL!	16.00
21.00	26.00	9.64	#NULL!	28.00	6	1	#NULL!	#NULL!	#NULL!	10.00
19.00	19.00	7.46	#NULL!	50.00	#NULL!	2	1.00	#NULL!	#NULL!	27.00
31.00	16.00	7.26	#NULL!	50.00	#NULL!	2	1.00	#NULL!	#NULL!	4.00
11.00	22.00	7.79	#NULL!	62.00	#NULL!	2	1.00	#NULL!	#NULL!	19.00
6.00	15.00	7.45	#NULL!	35.00	#NULL!	2	2.00	26.00	7.00	41.00
6.00	17.00	9.17	#NULL!	23.00	#NULL!	2	2.00	30.00	30.00	31.00
19.00	12.00	9.92	#NULL!	39.00	#NULL!	2	1.00	#NULL!	#NULL!	16.00
29.00	22.00	6.72	#NULL!	32.00	#NULL!	2	1.00	#NULL!	#NULL!	21.00
16.00	33.00	7.09	#NULL!	35.00	#NULL!	2	2.00	20.00	20.00	20.00
28.00	32.00	11.90	#NULL!	70.00	#NULL!	2	1.00	#NULL!	#NULL!	13.00
12.00	19.00	7.12	#NULL!	38.00	#NULL!	2	1.00	#NULL!	#NULL!	24.00
6.00	19.00	9.90	#NULL!	38.00	#NULL!	2	2.00	20.00	5.00	21.00
7.00	11.00	5.94	#NULL!	25.00	5	1	2.00	#NULL!	#NULL!	25.00
25.00	25.00	11.05	#NULL!	46.00	#NULL!	2	2.00	20.00	20.00	5.00
19.00	21.00	10.43	#NULL!	47.00	#NULL!	2	2.00	42.00	6.00	44.00
6.00	26.00	8.71	#NULL!	42.00	#NULL!	2	2.00	34.00	20.00	36.00
11.00	25.00	7.95	#NULL!	60.00	#NULL!	2	2.00	#NULL!	5.00	24.00
6.00	14.00	7.01	#NULL!	26.00	5	1	1.00	#NULL!	#NULL!	36.00
17.00	16.00	7.87	#NULL!	39.00	#NULL!	2	2.00	34.00	20.00	28.00
20.00	19.00	6.90	#NULL!	44.00	#NULL!	2	2.00	41.00	20.00	35.00
3.00	8.00	6.15	#NULL!	28.00	#NULL!	2	2.00	27.00	20.00	28.00
8.00	19.00	9.57	#NULL!	37.00	#NULL!	2	2.00	20.00	15.00	20.00
7.00	9.00	6.04	#NULL!	20.00	#NULL!	2	2.00	20.00	10.00	34.00
14.00	9.00	5.47	#NULL!	37.00	#NULL!	2	2.00	30.00	15.00	31.00
7.00	29.00	11.58	#NULL!	38.00	#NULL!	2	2.00	25.00	4.00	31.00
27.00	16.00	7.75	#NULL!	49.00	#NULL!	2	#NULL!	#NULL!	#NULL!	23.00
5.00	10.00	8.86	#NULL!	39.00	#NULL!	2	2.00	17.00	5.00	33.00
12.00	32.00	7.10	#NULL!	32.00	5	1	2.00	30.00	5.00	22.00
20.00	15.00	8.13	#NULL!	43.00	#NULL!	2	2.00	30.00	5.00	27.00
4.00	15.00	6.59	#NULL!	27.00	3	1	1.00	#NULL!	#NULL!	30.00
4.00	9.00	6.81	#NULL!	14.00	5	1	1.00	#NULL!	#NULL!	30.00
15.00	31.00	9.44	#NULL!	34.00	#NULL!	2	1.00	#NULL!	#NULL!	22.00
27.00	21.00	6.74	#NULL!	39.00	#NULL!	2	1.00	#NULL!	#NULL!	6.00
2.00	16.00	8.69	#NULL!	23.00	#NULL!	2	1.00	#NULL!	#NULL!	22.00
25.00	15.00	10.43	#NULL!	40.00	#NULL!	2	2.00	20.00	5.00	23.00
16.00	15.00	6.76	#NULL!	39.00	#NULL!	2	2.00	20.00	5.00	29.00
27.00	23.00	8.23	#NULL!	39.00	#NULL!	2	1.00	#NULL!	#NULL!	2.00
25.00	19.00	8.84	#NULL!	50.00	#NULL!	2	#NULL!	#NULL!	#NULL!	13.00
16.00	14.00	8.52	#NULL!	31.00	#NULL!	2	1.00	#NULL!	#NULL!	55.00
13.00	7.00	7.56	#NULL!	30.00	#NULL!	2	1.00	#NULL!	#NULL!	17.00
30.00	24.00	7.18	#NULL!	59.00	#NULL!	2	1.00	#NULL!	#NULL!	17.00

29.00	32.00	10.65	#NULL!	47.00	#NULL!	2	1.00	#NULL!	#NULL!	25.00
26.00	28.00	5.71	#NULL!	43.00	#NULL!	2	1.00	#NULL!	#NULL!	21.00
7.00	20.00	8.17	#NULL!	39.00	#NULL!	2	1.00	#NULL!	#NULL!	10.00
26.00	9.00	7.15	#NULL!	53.00	#NULL!	2	1.00	#NULL!	#NULL!	7.00
8.00	12.00	6.94	#NULL!	28.00	#NULL!	2	#NULL!	#NULL!	#NULL!	23.00
10.00	7.00	3.82	#NULL!	27.00	#NULL!	2	2.00	20.00	15.00	3.00
7.00	11.00	6.72	#NULL!	18.00	#NULL!	2	1.00	#NULL!	#NULL!	31.00
24.00	10.00	7.56	#NULL!	44.00	#NULL!	2	2.00	23.00	20.00	21.00
24.00	7.00	6.93	#NULL!	30.00	#NULL!	2	1.00	#NULL!	#NULL!	20.00
29.00	21.00	8.20	#NULL!	54.00	#NULL!	2	1.00	#NULL!	#NULL!	18.00
14.00	5.00	5.94	#NULL!	48.00	#NULL!	2	1.00	#NULL!	#NULL!	29.00
16.00	8.00	6.59	#NULL!	18.00	#NULL!	2	1.00	#NULL!	#NULL!	26.00
13.00	23.00	5.26	#NULL!	25.00	#NULL!	2	1.00	#NULL!	#NULL!	10.00
26.00	14.00	6.74	#NULL!	32.00	#NULL!	2	1.00	#NULL!	#NULL!	13.00
19.00	14.00	8.41	#NULL!	46.00	#NULL!	2	1.00	#NULL!	#NULL!	18.00
20.00	42.00	8.11	#NULL!	40.00	#NULL!	2	1.00	#NULL!	#NULL!	28.00
4.00	4.00	6.60	#NULL!	20.00	#NULL!	2	1.00	#NULL!	#NULL!	37.00
8.00	26.00	8.51	#NULL!	10.00	#NULL!	2	2.00	4.00	5.00	13.00
12.00	21.00	8.49	#NULL!	43.00	#NULL!	2	1.00	#NULL!	#NULL!	24.00
12.00	17.00	7.00	#NULL!	33.00	#NULL!	2	2.00	28.00	20.00	26.00
3.00	15.00	6.67	#NULL!	10.00	#NULL!	2	2.00	12.00	12.00	27.00
11.00	7.00	6.23	#NULL!	21.00	#NULL!	2	2.00	10.00	3.00	30.00
8.00	18.00	5.50	#NULL!	19.00	#NULL!	2	2.00	20.00	20.00	27.00
9.00	3.00	5.63	#NULL!	12.00	#NULL!	2	2.00	24.00	4.00	38.00
8.00	11.00	5.83	#NULL!	35.00	#NULL!	2	1.00	#NULL!	#NULL!	21.00
34.00	17.00	7.35	#NULL!	50.00	#NULL!	2	1.00	#NULL!	#NULL!	14.00
31.00	36.00	10.91	#NULL!	52.00	#NULL!	2	1.00	#NULL!	#NULL!	4.00
27.00	19.00	9.09	#NULL!	43.00	#NULL!	2	1.00	#NULL!	#NULL!	4.00
21.00	27.00	10.14	#NULL!	42.00	#NULL!	2	1.00	#NULL!	#NULL!	17.00
21.00	29.00	5.87	2.07	24.00	#NULL!	2	2.00	37.00	10.00	37.00
1.00	15.00	6.85	1.65	30.00	#NULL!	2	2.00	40.00	10.00	39.00
10.00	18.00	6.35	2.19	25.00	#NULL!	2	1.00	#NULL!	#NULL!	15.00
31.00	21.00	9.30	2.43	35.00	#NULL!	2	2.00	10.00	10.00	13.00
16.00	23.00	7.83	.90	28.00	#NULL!	2	2.00	20.00	6.00	30.00
21.00	12.00	4.84	1.28	40.00	#NULL!	2	1.00	#NULL!	#NULL!	17.00
2.00	20.00	7.53	1.16	13.00	#NULL!	2	2.00	35.00	5.00	35.00
27.00	17.00	9.44	2.27	42.00	#NULL!	2	#NULL!	#NULL!	#NULL!	#NULL!
11.00	7.00	9.86	1.95	33.00	#NULL!	2	1.00	#NULL!	#NULL!	1.00
12.00	15.00	7.46	−.19	46.00	#NULL!	2	1.00	#NULL!	#NULL!	27.00
16.00	15.00	10.46	1.91	45.00	#NULL!	2	#NULL!	#NULL!	#NULL!	#NULL!
7.00	18.00	10.20	2.29	38.00	#NULL!	2	1.00	#NULL!	#NULL!	3.00
18.00	15.00	10.39	1.39	40.00	#NULL!	2	#NULL!	#NULL!	#NULL!	#NULL!
23.00	21.00	8.49	1.25	45.00	#NULL!	2	#NULL!	#NULL!	#NULL!	#NULL!
9.00	11.00	6.53	1.29	40.00	#NULL!	2	1.00	#NULL!	#NULL!	24.00
11.00	18.00	6.20	1.77	36.00	#NULL!	2	1.00	#NULL!	#NULL!	19.00
18.00	18.00	8.87	1.37	35.00	#NULL!	2	1.00	#NULL!	#NULL!	22.00
19.00	23.00	7.18	1.42	35.00	5	1	1.00	#NULL!	#NULL!	36.00
15.00	15.00	7.81	2.13	12.00	#NULL!	2	1.00	#NULL!	#NULL!	16.00
6.00	3.00	6.91	.47	40.00	#NULL!	2	1.00	#NULL!	#NULL!	29.00
19.00	14.00	7.70	1.53	11.00	4	1	1.00	#NULL!	#NULL!	12.00
8.00	9.00	8.01	.84	20.00	#NULL!	2	2.00	45.00	#NULL!	29.00
10.00	10.00	8.38	.30	20.00	#NULL!	2	1.00	#NULL!	#NULL!	29.00
20.00	18.00	5.72	1.18	41.00	#NULL!	2	2.00	30.00	6.00	31.00
9.00	6.00	4.17	.03	37.00	#NULL!	2	2.00	21.00	10.00	40.00
9.00	17.00	7.59	1.63	41.00	#NULL!	2	#NULL!	#NULL!	#NULL!	#NULL!
25.00	19.00	5.23	1.72	41.00	#NULL!	2	2.00	39.00	3.00	21.00
30.00	28.00	7.13	1.25	60.00	#NULL!	2	1.00	#NULL!	#NULL!	28.00
8.00	6.00	4.77	1.04	13.00	#NULL!	2	2.00	30.00	10.00	31.00
15.00	22.00	6.94	2.01	26.00	#NULL!	2	2.00	20.00	10.00	20.00
7.00	12.00	9.74	1.53	43.00	#NULL!	2	1.00	#NULL!	#NULL!	29.00
26.00	23.00	8.08	1.37	45.00	#NULL!	2	1.00	#NULL!	#NULL!	24.00
8.00	22.00	6.64	1.43	45.00	#NULL!	2	1.00	#NULL!	#NULL!	26.00

16.00	13.00	9.70	1.80	47.00	#NULL!	2	1.00	#NULL!	#NULL!	36.00
16.00	22.00	7.56	1.72	45.00	6	1	1.00	#NULL!	#NULL!	21.00
17.00	12.00	7.91	1.13	27.00	4	1	1.00	#NULL!	#NULL!	21.00
7.00	4.00	7.31	1.34	30.00	#NULL!	2	1.00	#NULL!	#NULL!	13.00
20.00	14.00	6.74	.28	10.00	#NULL!	2	1.00	#NULL!	#NULL!	21.00
23.00	21.00	6.25	2.00	40.00	3	1	1.00	#NULL!	#NULL!	15.00
15.00	16.00	9.93	2.20	30.00	#NULL!	2	1.00	#NULL!	#NULL!	25.00
11.00	18.00	8.61	2.53	13.00	#NULL!	2	#NULL!	#NULL!	#NULL!	#NULL!
23.00	22.00	9.12	2.15	57.00	#NULL!	2	1.00	#NULL!	#NULL!	14.00
4.00	9.00	10.03	1.04	34.00	#NULL!	2	1.00	#NULL!	#NULL!	15.00
14.00	15.00	7.95	.31	25.00	#NULL!	2	2.00	#NULL!	20.00	11.00
4.00	7.00	4.16	.63	21.00	#NULL!	2	2.00	28.00	9.00	29.00
4.00	16.00	6.94	1.21	27.00	#NULL!	2	1.00	#NULL!	#NULL!	32.00
19.00	22.00	4.67	2.02	30.00	#NULL!	2	2.00	#NULL!	#NULL!	23.00
13.00	21.00	8.15	1.68	43.00	#NULL!	2	2.00	20.00	10.00	24.00
.00	10.00	4.57	1.31	27.00	#NULL!	2	2.00	36.00	4.00	36.00
31.00	23.00	6.33	2.09	47.00	#NULL!	2	2.00	20.00	3.00	19.00
12.00	24.00	7.22	1.93	21.00	#NULL!	2	2.00	12.00	#NULL!	35.00
7.00	15.00	8.09	1.48	30.00	#NULL!	2	2.00	41.00	5.00	42.00
23.00	23.00	8.23	1.31	33.00	#NULL!	2	1.00	#NULL!	#NULL!	3.00
9.00	15.00	5.94	–.07	26.00	4	1	2.00	42.00	7.00	37.00
8.00	19.00	8.15	.86	29.00	#NULL!	2	2.00	2.00	5.00	33.00
2.00	18.00	5.44	.40	6.00	#NULL!	2	2.00	34.00	3.00	32.00
14.00	17.00	7.19	.55	15.00	3	1	2.00	26.00	8.00	25.00
17.00	8.00	8.25	1.71	13.00	#NULL!	2	2.00	36.00	10.00	30.00
7.00	24.00	8.05	1.80	40.00	#NULL!	2	1.00	#NULL!	#NULL!	24.00
8.00	7.00	5.05	1.60	29.00	#NULL!	2	1.00	#NULL!	#NULL!	14.00
16.00	35.00	8.92	1.56	50.00	#NULL!	2	1.00	#NULL!	#NULL!	38.00
27.00	12.00	9.60	1.05	38.00	#NULL!	2	1.00	#NULL!	#NULL!	17.00
6.00	15.00	9.66	.45	23.00	#NULL!	2	1.00	#NULL!	#NULL!	15.00
14.00	4.00	6.65	1.78	49.00	#NULL!	2	2.00	20.00	2.00	33.00
26.00	18.00	4.59	1.50	40.00	#NULL!	2	1.00	#NULL!	#NULL!	31.00
14.00	21.00	10.62	1.72	30.00	#NULL!	2	#NULL!	#NULL!	#NULL!	#NULL!
2.00	10.00	8.16	.93	17.00	#NULL!	2	1.00	#NULL!	#NULL!	27.00
9.00	10.00	6.72	.98	37.00	5	1	1.00	#NULL!	#NULL!	23.00
15.00	11.00	4.66	.09	14.00	#NULL!	2	1.00	#NULL!	#NULL!	23.00
22.00	12.00	7.10	1.49	45.00	#NULL!	2	1.00	#NULL!	#NULL!	10.00
31.00	14.00	6.11	.90	30.00	#NULL!	2	1.00	#NULL!	#NULL!	16.00
28.00	20.00	8.32	2.88	42.00	#NULL!	2	2.00	20.00	10.00	13.00
11.00	19.00	8.15	1.93	29.00	#NULL!	2	2.00	4.00	10.00	1.00
11.00	21.00	7.52	1.72	29.00	#NULL!	2	1.00	#NULL!	#NULL!	38.00
29.00	25.00	7.88	3.36	36.00	#NULL!	2	2.00	10.00	20.00	22.00
7.00	11.00	6.16	1.15	33.00	#NULL!	2	1.00	#NULL!	#NULL!	41.00
26.00	14.00	7.08	2.56	39.00	#NULL!	2	#NULL!	#NULL!	#NULL!	#NULL!
12.00	14.00	8.34	.55	31.00	4	1	1.00	#NULL!	#NULL!	7.00
30.00	6.00	6.64	.72	41.00	#NULL!	2	#NULL!	#NULL!	#NULL!	#NULL!
11.00	14.00	7.87	1.29	39.00	6	1	1.00	#NULL!	#NULL!	6.00
20.00	15.00	7.14	1.72	45.00	#NULL!	2	1.00	#NULL!	#NULL!	29.00
14.00	13.00	4.77	1.18	35.00	#NULL!	2	1.00	#NULL!	#NULL!	22.00
26.00	16.00	9.33	1.46	24.00	#NULL!	2	1.00	#NULL!	#NULL!	8.00
18.00	15.00	7.31	.93	14.00	#NULL!	2	1.00	#NULL!	#NULL!	32.00
18.00	23.00	6.60	–.06	20.00	#NULL!	2	#NULL!	#NULL!	#NULL!	#NULL!
17.00	19.00	5.77	1.45	25.00	#NULL!	2	1.00	#NULL!	#NULL!	24.00
14.00	15.00	8.91	.88	10.00	#NULL!	2	#NULL!	#NULL!	#NULL!	#NULL!
28.00	16.00	7.45	2.67	45.00	#NULL!	2	2.00	20.00	5.00	12.00
13.00	24.00	8.66	.91	37.00	5	1	2.00	33.00	8.00	23.00
19.00	17.00	6.29	2.47	56.00	#NULL!	2	1.00	#NULL!	#NULL!	9.00
22.00	27.00	7.69	1.70	21.00	#NULL!	2	1.00	#NULL!	#NULL!	7.00
18.00	20.00	4.31	1.51	36.00	#NULL!	2	1.00	#NULL!	#NULL!	39.00
10.00	9.00	5.79	.98	25.00	#NULL!	2	1.00	#NULL!	#NULL!	19.00
11.00	37.00	9.78	2.03	44.00	#NULL!	2	1.00	#NULL!	#NULL!	33.00
13.00	16.00	7.22	.76	12.00	#NULL!	2	2.00	25.00	10.00	23.00

28.00	17.00	6.65	2.99	46.00	#NULL!	2	1.00	#NULL!	#NULL!	22.00
11.00	17.00	4.31	-.40	27.00	#NULL!	2	2.00	20.00	13.00	22.00
10.00	22.00	4.04	.50	20.00	#NULL!	2	1.00	#NULL!	#NULL!	20.00
30.00	23.00	5.93	2.01	27.00	#NULL!	2	1.00	#NULL!	#NULL!	14.00
11.00	12.00	5.82	1.04	39.00	#NULL!	2	2.00	10.00	20.00	28.00
25.00	29.00	10.46	2.60	68.00	#NULL!	2	2.00	8.00	5.00	14.00
8.00	13.00	4.54	.08	40.00	#NULL!	2	2.00	18.00	24.00	12.00
20.00	14.00	10.23	3.27	41.00	#NULL!	2	2.00	18.00	25.00	20.00
8.00	16.00	6.36	1.15	36.00	#NULL!	2	2.00	20.00	10.00	13.00
2.00	23.00	9.11	1.11	18.00	4	1	2.00	20.00	25.00	23.00
23.00	27.00	4.24	2.80	38.00	#NULL!	2	1.00	#NULL!	#NULL!	29.00
8.00	33.00	7.00	2.22	38.00	#NULL!	2	2.00	38.00	10.00	35.00
6.00	30.00	6.56	1.60	38.00	#NULL!	2	2.00	28.00	30.00	35.00
28.00	20.00	9.65	3.02	50.00	#NULL!	2	2.00	3.00	15.00	10.00
21.00	17.00	7.20	.92	44.00	#NULL!	2	#NULL!	#NULL!	#NULL!	16.00
29.00	28.00	8.10	1.99	36.00	#NULL!	2	#NULL!	#NULL!	#NULL!	7.00
24.00	20.00	8.62	2.54	18.00	3	1	#NULL!	#NULL!	#NULL!	14.00
19.00	24.00	12.06	1.07	35.00	#NULL!	2	#NULL!	#NULL!	#NULL!	#NULL!
27.00	18.00	8.02	2.93	65.00	#NULL!	2	#NULL!	#NULL!	#NULL!	#NULL!
11.00	7.00	4.92	.49	26.00	#NULL!	2	1.00	#NULL!	#NULL!	36.00
18.00	21.00	6.57	1.23	37.00	#NULL!	2	1.00	#NULL!	#NULL!	7.00
9.00	18.00	8.43	1.10	48.00	#NULL!	2	1.00	#NULL!	#NULL!	33.00
7.00	10.00	5.80	.06	10.00	#NULL!	2	1.00	#NULL!	#NULL!	18.00
6.00	21.00	9.24	1.09	28.00	#NULL!	2	1.00	#NULL!	#NULL!	37.00
10.00	10.00	8.46	1.47	39.00	#NULL!	2	1.00	#NULL!	#NULL!	26.00
33.00	19.00	10.40	2.31	48.00	#NULL!	2	1.00	#NULL!	#NULL!	1.00
29.00	29.00	8.67	3.46	53.00	#NULL!	2	#NULL!	#NULL!	#NULL!	#NULL!
7.00	15.00	7.92	.83	29.00	#NULL!	2	#NULL!	#NULL!	#NULL!	#NULL!
10.00	18.00	7.98	1.13	29.00	#NULL!	2	1.00	#NULL!	#NULL!	37.00
33.00	30.00	10.65	2.86	61.00	#NULL!	2	#NULL!	#NULL!	#NULL!	#NULL!
14.00	10.00	3.36	.98	40.00	#NULL!	2	2.00	10.00	10.00	14.00
12.00	14.00	5.86	1.29	40.00	#NULL!	2	2.00	10.00	10.00	22.00
16.00	23.00	7.91	1.54	20.00	#NULL!	2	2.00	10.00	10.00	29.00
12.00	25.00	11.05	1.29	22.00	#NULL!	2	2.00	10.00	10.00	32.00
8.00	16.00	5.96	.68	22.00	#NULL!	2	2.00	10.00	10.00	24.00
13.00	9.00	6.20	.59	35.00	#NULL!	2	2.00	10.00	10.00	21.00
27.00	21.00	10.55	2.84	50.00	#NULL!	2	2.00	10.00	10.00	1.00
15.00	27.00	9.55	2.35	52.00	#NULL!	2	2.00	10.00	10.00	36.00
4.00	13.00	8.92	1.71	13.00	4	1	2.00	30.00	20.00	22.00
10.00	12.00	10.25	.63	20.00	#NULL!	2	2.00	40.00	20.00	36.00
4.00	18.00	6.22	.29	18.00	#NULL!	2	2.00	10.00	10.00	40.00
31.00	19.00	6.99	1.86	18.00	#NULL!	2	2.00	10.00	10.00	21.00
9.00	13.00	7.89	2.41	39.00	#NULL!	2	2.00	30.00	20.00	31.00
9.00	14.00	6.48	1.24	39.00	#NULL!	2	2.00	10.00	10.00	30.00
6.00	10.00	6.34	.80	4.00	#NULL!	2	2.00	10.00	10.00	23.00
27.00	20.00	9.37	1.19	31.00	#NULL!	2	2.00	10.00	10.00	22.00
10.00	13.00	9.36	1.96	42.00	#NULL!	2	1.00	#NULL!	#NULL!	31.00
16.00	19.00	11.59	2.46	50.00	#NULL!	2	#NULL!	#NULL!	#NULL!	#NULL!
29.00	28.00	6.18	3.41	35.00	#NULL!	2	2.00	10.00	15.00	22.00
18.00	27.00	6.21	2.47	20.00	#NULL!	2	1.00	#NULL!	#NULL!	30.00
26.00	19.00	8.15	1.36	42.00	4	1	2.00	25.00	10.00	28.00
11.00	17.00	8.81	1.39	34.00	#NULL!	2	#NULL!	#NULL!	#NULL!	#NULL!
17.00	13.00	11.30	1.71	36.00	#NULL!	2	1.00	#NULL!	#NULL!	36.00
14.00	17.00	8.07	1.75	39.00	#NULL!	2	1.00	#NULL!	#NULL!	34.00
16.00	5.00	8.45	1.19	31.00	#NULL!	2	2.00	29.00	2.00	31.00
35.00	22.00	11.50	2.55	23.00	#NULL!	2	#NULL!	#NULL!	#NULL!	#NULL!
17.00	15.00	8.64	2.20	44.00	#NULL!	2	2.00	15.00	12.00	20.00
10.00	19.00	7.33	1.15	8.00	4	1	1.00	#NULL!	#NULL!	37.00
12.00	15.00	7.53	1.41	21.00	#NULL!	2	1.00	#NULL!	#NULL!	24.00
14.00	9.00	7.55	1.06	44.00	3	1	1.00	#NULL!	#NULL!	27.00
20.00	16.00	5.35	1.05	10.00	#NULL!	2	2.00	17.00	10.00	17.00
12.00	26.00	6.47	1.39	26.00	#NULL!	2	2.00	30.00	10.00	37.00

17.00	13.00	9.41	1.23	20.00	#NULL!	2	2.00	30.00	15.00	31.00
5.00	15.00	5.79	.49	15.00	4	1	1.00	#NULL!	#NULL!	25.00
28.00	25.00	7.56	1.66	29.00	#NULL!	2	2.00	15.00	8.00	15.00
12.00	19.00	7.07	2.14	39.00	#NULL!	2	2.00	5.00	5.00	1.00
21.00	23.00	8.69	2.99	35.00	#NULL!	2	2.00	10.00	10.00	15.00
16.00	10.00	7.17	1.46	39.00	#NULL!	2	2.00	10.00	10.00	22.00
7.00	15.00	7.18	.98	28.00	#NULL!	2	2.00	10.00	10.00	24.00
13.00	13.00	8.02	1.94	31.00	#NULL!	2	2.00	22.00	8.00	24.00
8.00	25.00	5.37	.99	46.00	#NULL!	2	2.00	10.00	10.00	37.00
34.00	17.00	7.09	3.10	48.00	#NULL!	2	1.00	#NULL!	#NULL!	8.00
14.00	12.00	9.54	1.49	44.00	#NULL!	2	1.00	#NULL!	#NULL!	18.00
5.00	18.00	6.44	2.23	42.00	#NULL!	2	2.00	30.00	15.00	26.00
18.00	27.00	11.98	1.48	40.00	#NULL!	2	1.00	#NULL!	#NULL!	16.00
10.00	24.00	8.65	2.07	43.00	3	1	2.00	18.00	20.00	20.00
13.00	43.00	8.48	2.85	31.00	#NULL!	2	2.00	18.00	10.00	27.00
23.00	28.00	10.03	2.38	39.00	#NULL!	2	2.00	10.00	12.00	18.00
7.00	21.00	7.28	1.63	24.00	#NULL!	2	1.00	#NULL!	#NULL!	19.00
13.00	30.00	10.70	3.05	40.00	#NULL!	2	1.00	#NULL!	#NULL!	16.00
7.00	17.00	7.55	2.99	41.00	#NULL!	2	2.00	37.00	15.00	35.00
24.00	23.00	8.80	1.88	39.00	5	1	1.00	#NULL!	#NULL!	14.00
21.00	18.00	8.73	1.60	43.00	5	1	2.00	26.00	1.00	22.00
24.00	16.00	8.24	1.12	34.00	#NULL!	2	2.00	20.00	6.00	15.00
2.00	8.00	6.29	1.22	.00	#NULL!	2	2.00	35.00	10.00	33.00

ge of onset	f first treatment	t hospitalization	italizations	ANSS score	positive score	negative score	opathology of PANSS
22.00	22.00	22.00	3.00	70.00	12.00	20.00	32.00
22.00	29.00	29.00	3.00	63.00	8.00	22.00	28.00
21.00	21.00	21.00	5.00	89.00	23.00	26.00	35.00
23.00	23.00	23.00	7.00	51.00	7.00	19.00	22.00
25.00	30.00	30.00	9.00	68.00	9.00	24.00	28.00
18.00	31.00	31.00	2.00	79.00	7.00	25.00	38.00
19.00	19.00	19.00	4.00	90.00	9.00	31.00	43.00
19.00	22.00	22.00	5.00	74.00	11.00	25.00	33.00
23.00	23.00	23.00	7.00	66.00	9.00	29.00	25.00
25.00	25.00	25.00	6.00	62.00	11.00	25.00	23.00
#NULL!	33.00	33.00	4.00	68.00	13.00	26.00	25.00
22.00	22.00	22.00	6.00	66.00	11.00	21.00	30.00
28.00	28.00	28.00	4.00	89.00	17.00	23.00	41.00
25.00	28.00	28.00	4.00	59.00	9.00	20.00	26.00
17.00	17.00	20.00	1.00	76.00	21.00	20.00	29.00
18.00	18.00	18.00	4.00	51.00	8.00	13.00	27.00
20.00	20.00	38.00	5.00	61.00	10.00	24.00	24.00
30.00	30.00	30.00	3.00	82.00	18.00	28.00	33.00
25.00	26.00	26.00	6.00	93.00	22.00	25.00	41.00
29.00	29.00	37.00	8.00	80.00	9.00	29.00	36.00
28.00	33.00	33.00	1.00	86.00	18.00	25.00	39.00
28.00	36.00	36.00	3.00	68.00	17.00	21.00	27.00
40.00	40.00	40.00	1.00	48.00	9.00	12.00	24.00
26.00	26.00	#NULL!	4.00	69.00	26.00	12.00	28.00
17.00	17.00	17.00	2.00	76.00	23.00	11.00	35.00
18.00	35.00	35.00	4.00	59.00	15.00	18.00	23.00
19.00	19.00	19.00	3.00	96.00	19.00	33.00	41.00
27.00	27.00	27.00	2.00	77.00	21.00	15.00	35.00
27.00	27.00	27.00	2.00	84.00	25.00	13.00	40.00
22.00	24.00	24.00	4.00	53.00	17.00	7.00	26.00
17.00	17.00	17.00	5.00	44.00	7.00	10.00	24.00
21.00	21.00	21.00	4.00	43.00	7.00	10.00	23.00
21.00	21.00	21.00	3.00	96.00	27.00	21.00	36.00
44.00	44.00	44.00	3.00	68.00	19.00	8.00	38.00
16.00	16.00	21.00	2.00	38.00	11.00	7.00	17.00
19.00	19.00	20.00	13.00	48.00	13.00	14.00	18.00
22.00	31.00	31.00	4.00	62.00	17.00	13.00	29.00
22.00	22.00	25.00	4.00	67.00	21.00	14.00	29.00
22.00	22.00	31.00	4.00	46.00	10.00	12.00	21.00
25.00	25.00	25.00	3.00	40.00	9.00	10.00	17.00
19.00	23.00	23.00	4.00	54.00	13.00	14.00	24.00
25.00	25.00	25.00	3.00	52.00	13.00	15.00	21.00
20.00	20.00	20.00	4.00	69.00	15.00	14.00	32.00
20.00	20.00	20.00	5.00	63.00	13.00	17.00	30.00
20.00	20.00	20.00	5.00	65.00	16.00	18.00	28.00
29.00	41.00	44.00	2.00	46.00	8.00	16.00	19.00
19.00	25.00	25.00	8.00	62.00	15.00	19.00	25.00
29.00	#NULL!	#NULL!	#NULL!	55.00	17.00	13.00	22.00
20.00	20.00	40.00	1.00	59.00	15.00	15.00	26.00
27.00	27.00	27.00	3.00	56.00	16.00	12.00	25.00
20.00	21.00	21.00	3.00	62.00	12.00	19.00	28.00
17.00	18.00	21.00	1.00	50.00	10.00	17.00	20.00
16.00	16.00	20.00	1.00	76.00	16.00	22.00	29.00
25.00	26.00	26.00	2.00	43.00	8.00	14.00	18.00
21.00	21.00	21.00	4.00	55.00	13.00	16.00	23.00
13.00	13.00	41.00	1.00	46.00	9.00	13.00	21.00
28.00	29.00	29.00	2.00	55.00	9.00	17.00	26.00
20.00	20.00	20.00	2.00	58.00	15.00	16.00	24.00
26.00	30.00	30.00	5.00	55.00	16.00	13.00	23.00
31.00	35.00	35.00	4.00	50.00	8.00	18.00	21.00
32.00	32.00	32.00	5.00	36.00	7.00	9.00	17.00

20.00	#NULL!	#NULL!	#NULL!	57.00	13.00	10.00	29.00
22.00	22.00	22.00	4.00	49.00	17.00	8.00	21.00
33.00	36.00	36.00	8.00	44.00	7.00	14.00	20.00
13.00	13.00	13.00	4.00	41.00	7.00	14.00	17.00
35.00	35.00	35.00	3.00	58.00	13.00	18.00	24.00
37.00	37.00	37.00	4.00	44.00	10.00	12.00	19.00
25.00	26.00	26.00	5.00	77.00	20.00	21.00	33.00
30.00	30.00	30.00	7.00	59.00	20.00	11.00	25.00
34.00	35.00	35.00	5.00	51.00	8.00	14.00	26.00
19.00	19.00	19.00	6.00	62.00	10.00	22.00	27.00
27.00	27.00	27.00	7.00	51.00	7.00	17.00	24.00
19.00	19.00	19.00	4.00	48.00	7.00	19.00	19.00
35.00	35.00	35.00	6.00	63.00	15.00	15.00	30.00
28.00	31.00	38.00	2.00	37.00	7.00	7.00	20.00
26.00	26.00	26.00	5.00	64.00	17.00	12.00	31.00
24.00	24.00	24.00	6.00	39.00	7.00	11.00	18.00
18.00	18.00	18.00	6.00	71.00	10.00	28.00	30.00
24.00	24.00	25.00	3.00	61.00	17.00	16.00	25.00
19.00	20.00	20.00	3.00	42.00	8.00	10.00	21.00
27.00	29.00	29.00	5.00	63.00	13.00	24.00	23.00
19.00	19.00	19.00	3.00	52.00	7.00	21.00	21.00
14.00	14.00	14.00	3.00	65.00	8.00	30.00	24.00
19.00	19.00	19.00	4.00	47.00	8.00	13.00	23.00
22.00	22.00	22.00	5.00	52.00	7.00	21.00	21.00
23.00	23.00	23.00	7.00	56.00	9.00	18.00	26.00
30.00	30.00	30.00	2.00	46.00	11.00	12.00	20.00
24.00	28.00	28.00	1.00	59.00	19.00	7.00	30.00
32.00	32.00	32.00	4.00	48.00	9.00	12.00	24.00
29.00	34.00	34.00	4.00	47.00	11.00	9.00	24.00
20.00	20.00	20.00	2.00	57.00	10.00	20.00	24.00
19.00	19.00	19.00	12.00	59.00	10.00	19.00	27.00
11.00	11.00	11.00	3.00	57.00	8.00	19.00	27.00
24.00	24.00	24.00	12.00	60.00	13.00	16.00	28.00
19.00	19.00	30.00	2.00	72.00	18.00	20.00	31.00
26.00	35.00	35.00	2.00	69.00	17.00	16.00	33.00
24.00	24.00	24.00	7.00	61.00	9.00	23.00	26.00
#NULL!	#NULL!	#NULL!	#NULL!	#NULL!	#NULL!	#NULL!	#NULL!
22.00	23.00	23.00	1.00	67.00	16.00	14.00	34.00
32.00	32.00	32.00	5.00	60.00	14.00	15.00	28.00
#NULL!	#NULL!	#NULL!	#NULL!	#NULL!	#NULL!	#NULL!	#NULL!
39.00	40.00	40.00	3.00	75.00	15.00	20.00	37.00
#NULL!	#NULL!	#NULL!	#NULL!	#NULL!	#NULL!	#NULL!	#NULL!
#NULL!	#NULL!	#NULL!	#NULL!	#NULL!	#NULL!	#NULL!	#NULL!
23.00	26.00	26.00	4.00	63.00	15.00	17.00	28.00
39.00	53.00	53.00	2.00	57.00	15.00	14.00	25.00
21.00	21.00	21.00	2.00	65.00	20.00	14.00	28.00
15.00	30.00	30.00	2.00	78.00	19.00	21.00	35.00
20.00	20.00	35.00	2.00	68.00	17.00	23.00	25.00
24.00	24.00	24.00	2.00	79.00	15.00	28.00	33.00
24.00	31.00	31.00	2.00	75.00	19.00	20.00	33.00
24.00	24.00	26.00	5.00	71.00	19.00	13.00	34.00
32.00	32.00	32.00	10.00	56.00	7.00	22.00	24.00
20.00	20.00	20.00	9.00	62.00	14.00	9.00	34.00
19.00	19.00	19.00	5.00	53.00	7.00	22.00	21.00
#NULL!	#NULL!	#NULL!	#NULL!	#NULL!	#NULL!	#NULL!	#NULL!
35.00	35.00	43.00	3.00	43.00	7.00	14.00	19.00
23.00	23.00	29.00	3.00	45.00	7.00	15.00	20.00
22.00	22.00	22.00	6.00	77.00	18.00	21.00	33.00
19.00	19.00	19.00	4.00	46.00	7.00	17.00	19.00
18.00	18.00	22.00	3.00	63.00	13.00	17.00	28.00
29.00	29.00	45.00	1.00	48.00	7.00	13.00	23.00
26.00	26.00	41.00	3.00	58.00	7.00	19.00	27.00

21.00	21.00	21.00	9.00	45.00	8.00	13.00	19.00
26.00	26.00	26.00	7.00	70.00	24.00	12.00	31.00
23.00	23.00	28.00	3.00	94.00	24.00	22.00	43.00
40.00	40.00	40.00	9.00	59.00	9.00	17.00	29.00
22.00	22.00	26.00	3.00	74.00	16.00	23.00	32.00
34.00	34.00	40.00	4.00	67.00	23.00	11.00	28.00
19.00	19.00	19.00	4.00	44.00	7.00	14.00	20.00
#NULL!	#NULL!	#NULL!	#NULL!	#NULL!	#NULL!	#NULL!	#NULL!
29.00	29.00	29.00	5.00	41.00	7.00	13.00	18.00
31.00	31.00	31.00	9.00	63.00	10.00	24.00	24.00
39.00	41.00	41.00	2.00	45.00	7.00	10.00	25.00
20.00	28.00	28.00	2.00	79.00	15.00	25.00	34.00
18.00	18.00	18.00	3.00	62.00	9.00	20.00	30.00
22.00	24.00	24.00	3.00	74.00	20.00	10.00	38.00
24.00	27.00	27.00	2.00	50.00	8.00	14.00	24.00
22.00	22.00	22.00	3.00	61.00	9.00	23.00	25.00
20.00	21.00	21.00	7.00	45.00	7.00	14.00	21.00
18.00	23.00	25.00	7.00	77.00	12.00	24.00	36.00
17.00	17.00	17.00	9.00	50.00	9.00	12.00	26.00
35.00	35.00	35.00	3.00	72.00	21.00	17.00	30.00
21.00	22.00	22.00	4.00	80.00	20.00	23.00	32.00
22.00	22.00	22.00	9.00	63.00	11.00	18.00	29.00
19.00	20.00	20.00	4.00	59.00	7.00	25.00	24.00
19.00	19.00	19.00	5.00	65.00	18.00	10.00	32.00
24.00	24.00	24.00	6.00	48.00	7.00	19.00	19.00
19.00	20.00	20.00	5.00	75.00	16.00	25.00	31.00
30.00	31.00	31.00	5.00	61.00	8.00	23.00	27.00
17.00	29.00	33.00	5.00	41.00	7.00	8.00	23.00
32.00	32.00	32.00	6.00	84.00	22.00	20.00	39.00
35.00	49.00	49.00	2.00	57.00	10.00	13.00	29.00
20.00	24.00	24.00	3.00	47.00	9.00	9.00	24.00
16.00	17.00	17.00	4.00	71.00	16.00	18.00	32.00
#NULL!	#NULL!	#NULL!	#NULL!	#NULL!	#NULL!	#NULL!	#NULL!
22.00	45.00	45.00	1.00	63.00	7.00	29.00	24.00
23.00	23.00	23.00	6.00	77.00	26.00	16.00	31.00
17.00	17.00	42.00	1.00	86.00	16.00	22.00	45.00
45.00	45.00	45.00	2.00	46.00	7.00	12.00	24.00
34.00	34.00	34.00	7.00	53.00	12.00	12.00	26.00
32.00	33.00	33.00	5.00	47.00	7.00	14.00	22.00
19.00	19.00	19.00	2.00	59.00	8.00	21.00	27.00
20.00	20.00	20.00	10.00	55.00	7.00	15.00	30.00
18.00	22.00	22.00	4.00	57.00	8.00	19.00	27.00
15.00	15.00	27.00	7.00	56.00	7.00	21.00	25.00
#NULL!	#NULL!	#NULL!	#NULL!	#NULL!	#NULL!	#NULL!	#NULL!
41.00	43.00	43.00	1.00	59.00	10.00	20.00	26.00
#NULL!	#NULL!	#NULL!	#NULL!	#NULL!	#NULL!	#NULL!	#NULL!
32.00	34.00	34.00	2.00	74.00	22.00	18.00	31.00
15.00	20.00	#NULL!	3.00	72.00	18.00	20.00	31.00
23.00	23.00	36.00	7.00	71.00	12.00	22.00	34.00
28.00	29.00	29.00	3.00	52.00	8.00	18.00	23.00
25.00	26.00	26.00	5.00	63.00	16.00	19.00	25.00
#NULL!	#NULL!	#NULL!	#NULL!	#NULL!	#NULL!	#NULL!	#NULL!
21.00	22.00	22.00	6.00	50.00	9.00	17.00	21.00
#NULL!	#NULL!	#NULL!	#NULL!	#NULL!	#NULL!	#NULL!	#NULL!
28.00	28.00	28.00	3.00	64.00	13.00	19.00	29.00
30.00	30.00	30.00	4.00	57.00	10.00	18.00	26.00
18.00	22.00	22.00	2.00	63.00	15.00	21.00	24.00
21.00	21.00	21.00	2.00	60.00	13.00	18.00	26.00
19.00	36.00	36.00	5.00	59.00	7.00	24.00	25.00
37.00	39.00	39.00	3.00	60.00	8.00	23.00	26.00
24.00	25.00	25.00	5.00	71.00	20.00	13.00	35.00
26.00	26.00	26.00	8.00	70.00	12.00	23.00	31.00

17.00	18.00	18.00	7.00	56.00	11.00	12.00	30.00
17.00	19.00	19.00	7.00	67.00	11.00	22.00	31.00
28.00	28.00	28.00	5.00	58.00	9.00	21.00	25.00
20.00	21.00	21.00	2.00	68.00	14.00	16.00	32.00
22.00	22.00	33.00	3.00	81.00	12.00	28.00	36.00
23.00	23.00	23.00	3.00	56.00	10.00	14.00	29.00
24.00	24.00	27.00	3.00	67.00	12.00	25.00	27.00
22.00	22.00	25.00	2.00	51.00	9.00	17.00	22.00
31.00	31.00	31.00	2.00	57.00	12.00	17.00	25.00
23.00	23.00	23.00	4.00	73.00	18.00	22.00	28.00
22.00	25.00	25.00	2.00	53.00	8.00	18.00	24.00
24.00	24.00	24.00	4.00	61.00	15.00	16.00	26.00
20.00	20.00	20.00	7.00	58.00	8.00	23.00	24.00
26.00	26.00	26.00	3.00	62.00	13.00	18.00	27.00
22.00	22.00	22.00	5.00	60.00	11.00	19.00	27.00
31.00	33.00	34.00	2.00	51.00	12.00	12.00	24.00
36.00	36.00	36.00	3.00	75.00	20.00	19.00	31.00
#NULL!	#NULL!	#NULL!	#NULL!	#NULL!	#NULL!	#NULL!	#NULL!
#NULL!	#NULL!	#NULL!	#NULL!	#NULL!	#NULL!	#NULL!	#NULL!
17.00	17.00	29.00	2.00	72.00	12.00	24.00	33.00
23.00	24.00	24.00	4.00	50.00	9.00	16.00	22.00
15.00	15.00	15.00	5.00	67.00	14.00	21.00	27.00
16.00	18.00	18.00	5.00	77.00	13.00	30.00	31.00
20.00	22.00	22.00	7.00	68.00	13.00	20.00	31.00
20.00	20.00	20.00	4.00	61.00	8.00	23.00	26.00
44.00	44.00	44.00	1.00	88.00	12.00	27.00	43.00
#NULL!	#NULL!	#NULL!	#NULL!	#NULL!	#NULL!	#NULL!	#NULL!
#NULL!	#NULL!	#NULL!	#NULL!	#NULL!	#NULL!	#NULL!	#NULL!
21.00	22.00	22.00	3.00	77.00	14.00	23.00	35.00
#NULL!	#NULL!	#NULL!	#NULL!	#NULL!	#NULL!	#NULL!	#NULL!
27.00	27.00	27.00	2.00	64.00	11.00	22.00	27.00
26.00	36.00	36.00	3.00	51.00	11.00	15.00	22.00
20.00	20.00	20.00	5.00	48.00	8.00	11.00	24.00
20.00	20.00	41.00	2.00	57.00	9.00	16.00	29.00
29.00	30.00	30.00	6.00	63.00	10.00	14.00	35.00
24.00	25.00	25.00	3.00	46.00	8.00	8.00	27.00
26.00	#NULL!	#NULL!	#NULL!	55.00	8.00	13.00	31.00
19.00	19.00	19.00	6.00	49.00	12.00	10.00	24.00
34.00	34.00	34.00	4.00	70.00	15.00	15.00	36.00
23.00	23.00	32.00	1.00	51.00	8.00	16.00	24.00
13.00	12.00	12.00	8.00	59.00	13.00	12.00	28.00
20.00	20.00	24.00	2.00	80.00	18.00	20.00	37.00
23.00	23.00	37.00	4.00	49.00	7.00	13.00	26.00
18.00	19.00	19.00	9.00	60.00	10.00	10.00	35.00
21.00	21.00	21.00	3.00	64.00	13.00	15.00	33.00
22.00	25.00	25.00	1.00	49.00	7.00	15.00	24.00
23.00	23.00	23.00	4.00	57.00	11.00	18.00	25.00
#NULL!	#NULL!	#NULL!	#NULL!	#NULL!	#NULL!	#NULL!	#NULL!
16.00	19.00	19.00	3.00	47.00	8.00	17.00	19.00
26.00	26.00	28.00	8.00	54.00	8.00	20.00	23.00
21.00	25.00	25.00	9.00	82.00	24.00	21.00	34.00
#NULL!	#NULL!	#NULL!	#NULL!	#NULL!	#NULL!	#NULL!	#NULL!
17.00	17.00	17.00	8.00	71.00	20.00	19.00	28.00
16.00	16.00	16.00	7.00	63.00	13.00	19.00	28.00
19.00	19.00	24.00	1.00	48.00	13.00	9.00	23.00
#NULL!	#NULL!	#NULL!	#NULL!	#NULL!	#NULL!	#NULL!	#NULL!
35.00	35.00	35.00	2.00	37.00	7.00	11.00	16.00
22.00	22.00	22.00	5.00	79.00	23.00	17.00	34.00
33.00	33.00	33.00	3.00	69.00	17.00	18.00	31.00
21.00	22.00	22.00	14.00	74.00	19.00	21.00	31.00
30.00	30.00	30.00	12.00	67.00	13.00	16.00	33.00
18.00	18.00	18.00	9.00	83.00	20.00	24.00	34.00

23.00	25.00	25.00	6.00	69.00	18.00	17.00	31.00
28.00	34.00	34.00	3.00	81.00	20.00	25.00	33.00
28.00	28.00	28.00	4.00	68.00	13.00	23.00	29.00
19.00	20.00	20.00	2.00	86.00	15.00	20.00	45.00
33.00	33.00	33.00	1.00	67.00	16.00	15.00	32.00
28.00	28.00	28.00	5.00	54.00	10.00	14.00	27.00
20.00	21.00	21.00	6.00	66.00	9.00	25.00	29.00
18.00	20.00	20.00	4.00	58.00	12.00	18.00	25.00
20.00	20.00	20.00	7.00	52.00	12.00	15.00	22.00
12.00	17.00	17.00	2.00	73.00	10.00	30.00	30.00
27.00	27.00	27.00	2.00	65.00	16.00	19.00	27.00
24.00	24.00	25.00	3.00	66.00	18.00	12.00	28.00
20.00	20.00	20.00	5.00	51.00	11.00	10.00	26.00
26.00	26.00	26.00	5.00	43.00	12.00	9.00	19.00
14.00	19.00	19.00	2.00	55.00	15.00	14.00	23.00
21.00	29.00	29.00	4.00	66.00	15.00	16.00	28.00
26.00	28.00	28.00	4.00	42.00	9.00	12.00	18.00
20.00	20.00	20.00	3.00	61.00	19.00	7.00	26.00
18.00	22.00	22.00	5.00	57.00	12.00	19.00	23.00
29.00	29.00	35.00	3.00	49.00	14.00	11.00	21.00
23.00	23.00	23.00	3.00	69.00	19.00	20.00	27.00
24.00	24.00	24.00	4.00	69.00	19.00	19.00	28.00
21.00	21.00	22.00	2.00	59.00	12.00	21.00	23.00

ID	age	sex	marria	birthday	educat	occupa	UPSA	UPSA	UPSA	STRO(	tmt	SC	hvlt
1	22	2	single	21-Feb-1986	6.00	blueco	8	11	19		0.20	34	19
8	22	1	single	22-Jul-1985	12.00	nurse	16	18	34	56	0.30	79	25
9	40	2	marrie	08-May-1967	6.00	bluecollar				48	0.23	42	19
11	36	1	single	09-Apr-1971	12.00	blueco	18	18	36	52	0.29	65	34
12	36	2	single	01-Jan-1972	12.00	whitec	20	20	40	61	0.26	75	31
14	49	2	single	01-May-1958	6.00	blueco	18	16	34	42	0.23	35	15
17	50	1	marrie	20-Feb-1957	15.00	whitec	20	18	38	45	0.23	47	22
20	49	2	marrie	09-Sep-1958	6.00	blueco	4	0	4	39	0.23	20	16
21	37	1	marrie	01-Oct-1970	15.00	whitec	20	18	38	51	0.26	55	28
33	23	1	single	14-Aug-1984	6.00	blueco	18	20	38	56	0.27	51	26
37	60	2	marrie	15-Feb-1948	6.00	blueco	18	18	36	26	0.26	50	20
46	60	1	marrie	18-Jun-1947	6.00	solider	20	18	38	34	0.29	45	23
48	49	2	marrie	01-Feb-1959	12.00	blueco	14	13	27	24	0.25	34	15
50	52	1	marrie	10-May-1955	6.00	blueco	16	16	32	50	0.27	43	24
51	49	1	single	01-Jun-1958	12.00	blueco	18	16	34	29	0.27	39	19
54	54	2	single	30-May-1953	6.00	blueco	18	16	34	20	0.26	35	22
58	29	1	marrie	05-Mar-1978	9.00	blueco	18	13	31	56	0.23	47	23
64	41	1	single	01-Mar-1967	9.00	blueco	20	16	36	39	0.29	47	26
65	23	2	single	01-Dec-1984	9.00	blueco	16	18	34	52	0.30	54	27
66	52	2	marrie	30-Oct-1955	9.00	blueco	10	11	21	44	0.28	40	25
68	59	2	marrie	15-Aug-1948	6.00	farmer	8	7	15	33	0.22	27	20
69	33	1	marrie	07-Jan-1975	12.00	blueco	18	20	38	66	0.30	61	32
74	56	1	marrie	13-Jun-1951	9.00	blueco	20	16	36	40	0.23	31	25
89	49	2	marrie	17-May-1958	6.00	blueco	8	7	15	42	0.24	41	15
90	45	1	marrie	12-Jun-1962	6.00	Engine	20	18	38	46	0.28	46	25
91	24	2	single	29-Jan-1983	6.00	whitec	18	16	34	53	0.29	66	35
93	44	1	marrie	11-Dec-1963	12.00	blueco	18	16	34	42	0.25	47	21
94	68	2	marrie	01-Jan-1940	6.00	blueco	16	16	32	40	0.22	16	21
100	55	2	marrie	08-Apr-1952	9.00	blueco	16	9	25	36	0.25	30	16
102	27	1	single	11-Apr-1980	6.00	blueco	6	4	10	17	0.25	37	23
103	57	2	single	02-Dec-1950	9.00	blueco	18	18	36	31	0.21	32	18
107	56	1	marrie	01-Sep-1951	12.00	whitec	20	16	36	51	0.30	56	23
111	21	1	single	24-Jun-1986	12.00	nurse	16	13	29	50	0.24	56	24
113	60	2	marrie	15-May-1947	6.00	other	14	18	32	34	0.22	31	24
121	45	1	marrie	27-Dec-1962	9.00	blueco	18	18	36	49	0.27	44	21
125	18	2	single	24-Feb-1989	6.00	blueco	16	7	23	38	0.28	41	20
139	55	1	marrie	03-Sep-1952	9.00	blueco	20	11	31	42	0.28	30	22
142	43	2	marrie	01-Dec-1964	12.00	blueco	20	16	36	52	0.29	45	22
143	55	1	marrie	10-Jul-1952	9.00	blueco	16	13	29	41	0.25	31	21
150	58	2	marrie	09-Oct-1949	6.00	blueco	20	13	33	27	0.24	41	25
151	21	2	single	25-Aug-1986	12.00	nurse	18	20	38	40	0.26	69	27
159	43	1	marrie	31-Dec-1964	9.00	blueco	18	16	34	15	0.26	26	22
168	48	1	marrie	08-Nov-1959	12.00	blueco	18	16	34	35	0.25	37	23
170	59	2	single	14-Jan-1949	6.00	blueco	12	7	19	30	0.23	22	17
173	45	2	marrie	26-Feb-1962	15.00	other	20	16	36	47	0.26	56	31
174	51	1	marrie	05-Nov-1956	6.00	blueco	14	7	21	42	0.23	29	17
177	32	1	marrie	18-Nov-1975	15.00	whitec	14	16	30	39	0.26	35	29
180	55	1	marrie	11-Feb-1952	9.00	blueco	16	9	25	10	0.24	44	23
186	49	1	single	14-Jul-1958	9.00	blueco	18	20	38	39	0.24	34	22
187	24	2	single	23-Jul-1983	9.00	blueco	20	16	36	45	0.26	54	26
190	19	2	single	15-Nov-1988	6.00	blueco	8	9	17	37	0.23	36	21
198	49	2	single	21-Aug-1958	12.00	blueco	12	13	25	42	0.24	22	20
199	32	1	marrie	15-Sep-1975	12.00	whitec	18	20	38	24	0.32	58	22
203	43	2	marrie	10-Nov-1964	9.00	blueco	14	18	32	63	0.25	51	25
207	35	1	marrie	07-Mar-1972	9.00	blueco	18	11	29	42	0.26	54	26
217	59	2	single	05-Apr-1948	6.00	blueco	12	18	30	39	0.22	32	19
218	58	1	single	02-Jan-1950	9.00	blueco	10	4	14	37	0.24	28	17
219	49	2	marrie	10-May-1958	6.00	blueco	20	13	33	36	0.23	34	22
227	36	1	marrie	14-Feb-1971	9.00	blueco	14	9	23	49	0.23	52	24
228	60	2	marrie	10-Sep-1947	6.00	blueco	8	7	15	18	0.19	17	16

229	47	2	marrie	15-Aug-1960	12.00	whitec	14	18	32	58	0.24	62	25
233	58	1	marrie	02-Aug-1949	6.00	blueco	18	16	34	40	0.25	19	19
235	57	2	single	01-Jun-1950	6.00	blueco	18	13	31	39	0.22	22	20
236	23	1	single	05-Jan-1985	6.00	Engine	18	13	31	69	0.24	58	30
237	60	2	marrie	06-Mar-1948	6.00	blueco	16	20	36	46	0.23	32	15
240	48	2	marrie	01-Mar-1959	12.00	whitec	18	16	34	42	0.24	50	25
241	46	2	marrie	01-Dec-1961	12.00	blueco	16	7	23	39	0.26	39	17
242	49	2	marrie	04-Jan-1959	9.00	blueco	10	16	26	28	0.24	37	15
243	51	2	marrie	22-Feb-1957	6.00	blueco	18	9	27	42	0.27	30	12
244	32	2	marrie	17-Jul-1975	6.00	self-en	16	7	23	41	0.24	50	18
246	60	1	marrie	12-Apr-1948	9.00	blueco	10	16	26	20	0.22	34	18
247	27	2	marrie	19-Sep-1980	9.00	self-en	20	13	33	49	0.27	54	30
248	53	2	single	01-Dec-1954	6.00	blueco	2	0	2	20	0.20	15	15
250	49	2	marrie	07-Jan-1959	12.00	blueco	16	13	29	61	0.28	40	17
257	28	1	marrie	01-Aug-1979	6.00	self-en	16	16	32	34	0.24	35	21
258	56	1	single	16-Aug-1951	6.00	blueco	18	13	31		0.23	26	10
259	56	1	single	05-Oct-1951	9.00	blueco	20	16	36	47	0.25	48	17
260	65	1	marrie	13-Apr-1943	6.00	blueco	4	4	8	16	0.21		7
262	58	2	marrie	21-Sep-1949	9.00	farmer	18	18	36	46	0.24	44	22
263	60	2	marrie	16-Dec-1947	6.00	other	12	11	23	49	0.21	37	23
266	60	2	marrie	07-Feb-1948	6.00	blueco	16	13	29	27	0.30	45	23
267	54	2	marrie	07-Mar-1954	15.00	blueco	16	13	29	58	0.24	48	16
269	21	1	single	02-Aug-1986	6.00	blueco	18	18	36	46	0.27	75	25
270	52	1	single	01-Feb-1956	6.00	blueco	18	18	36	35	0.24	37	25
271	44	1	single	29-Jun-1964	9.00	self-en	18	13	31		0.22	36	10
272	23	2	single	08-May-1985	6.00	blueco	18	20	38	49	0.25	65	28
273	44	2	single	13-Sep-1963	6.00	blueco	18	13	31	48	0.27	50	22
274	50	1	marrie	09-Oct-1957	6.00	blueco	16	2	18	29	0.23	31	8
275	55	2	marrie	25-Apr-1953	12.00	whitec	20	18	38	40	0.28	50	19
276	29	2	marrie	08-Dec-1978	6.00	blueco	14	11	25	42	0.25	48	24
277	45	2	marrie	15-Dec-1962	6.00	farmer	8	4	12	8	0.19	12	11
278	42	2	single	28-Aug-1965	6.00	blueco	8	4	12	14	0.20	23	17
279	28	1	marrie	04-Jan-1980	9.00	blueco	16	9	25	49	0.23	26	25
281	40	2	marrie	15-Oct-1967	6.00	other	14	7	21	42	0.23	45	19
283	58	1	marrie	18-Jan-1950	9.00	blueco	20	18	38	61	0.29	49	22
284	51	2	marrie	15-Jun-1957	9.00	blueco	16	11	27	44	0.31	50	25
287	48	2	marrie	19-Mar-1960	6.00	blueco	18	16	34	35	0.23	41	19
288	44	2	marrie	17-Mar-1964	6.00	self-en	16	4	20	50	0.23	33	20
289	58	2	marrie	12-Oct-1949	9.00	whitec	20	18	38	42	0.25	50	25
290	17	1	single	18-Mar-1991	9.00	blueco	18	16	34	34	0.25	57	27
291	44	2	marrie	15-Oct-1963	6.00	blueco	8	7	15	35	0.21	27	18
292	54	2	single	07-Nov-1953	6.00	blueco	14	13	27	45	0.24	34	15
293	55	2	marrie	01-Dec-1952	9.00	whitec	18	20	38	34	0.25	47	20
294	52	2	marrie	16-Mar-1956	9.00	blueco	10	18	28	28	0.24	34	22
295	45	2	marrie	10-Oct-1962	6.00	farmer	6	7	13	26	0.22	26	17
296	44	1	marrie	23-Oct-1963	12.00	blueco	18	18	36	67	0.27	51	29
297	18	1	single	30-May-1990	9.00	blueco	18	11	29	50	0.26	49	18
298	56	2	divorce	18-Jul-1951	9.00	farmer	12	4	16	34	0.27	35	20
301	34	1	single	23-Feb-1974	12.00	blueco	20	18	38	31	0.27	39	23
302	61	2	marrie	10-Feb-1947	6.00	farmer	14	13	27	32	0.22	22	20
303	47	1	marrie	27-Oct-1960	6.00	blueco	8	13	21	36	0.25	29	13
304	48	1	marrie	14-Nov-1959	9.00	blueco	16	9	25	33	0.24	28	23
305	30	2	single	04-Aug-1977	9.00	self-en	16	11	27	32	0.26	43	20
308	60	2	single	15-Mar-1948	6.00	blueco	12	16	28	60	0.25	20	17
310	59	2	marrie	01-Jan-1949	6.00	blueco	12	13	25	16	0.22	28	10
311	59	2	marrie	07-Mar-1949	6.00	blueco	14	7	21	44	0.22	21	11
312	20	1	single	25-Dec-1987	6.00	other	10	0	10	53	0.20	31	15
313	51	2	single	09-Feb-1957	9.00	whitec	20	18	38	33	0.28	30	16
314	45	2	marrie	16-Mar-1963	19.00	blueco	20	20	40	57	0.26	55	27
315	58	2	marrie	01-Dec-1949	6.00	blueco	12	9	21	38	0.19	21	17
316	58	1	single	20-Jan-1950	9.00	blueco	18	18	36	39	0.24	31	18
317	40	2	single	29-Nov-1967	6.00	blueco	16	7	23	46	0.24	32	20

318	36	2	marrie	26-Feb-1972	6.00	blueco	8	9	17	39	0.27	44	19
319	48	2	marrie	28-Dec-1959	9.00	blueco	20	16	36	45	0.24	35	20
320	59	1	marrie	17-Oct-1948	6.00	blueco	16	18	34	23	0.20	35	21
322	55	2	single	06-May-1953	6.00	blueco	16	13	29	46	0.25	36	19
323	45	1	marrie	29-Mar-1963	9.00	blueco	14	9	23	11	0.26	38	18
324	39	1	marrie	01-Jan-1969	6.00	self-en	14	2	16	22	0.23	37	20
325	61	1	marrie	21-Dec-1946	6.00	farmer	20	16	36	41	0.23	26	15
327	31	2	marrie	06-Dec-1976	12.00	whitec	20	18	38	56	0.25	62	27
328	52	2	single	31-Dec-1955	6.00	blueco	20	20	40	47	0.27	50	26
329	36	2	single	29-Jun-1972	12.00	other	20	13	33	58	0.26	70	28
330	45	1	marrie	20-Nov-1962	9.00	blueco	16	9	25	58	0.24	43	25
331	48	2	marrie	16-Jun-1960	6.00	blueco	12	13	25	39	0.22	19	24
332	40	2	marrie	23-Mar-1968	15.00	blueco	18	18	36	40	0.28	50	20
333	50	2	marrie	11-Oct-1957	6.00	blueco	18	18	36	36	0.27	38	21
334	34	2	marrie	24-Oct-1973	12.00	other	20	16	36	58	0.25	55	23
335	25	2	single	17-Aug-1982	6.00	blueco	10	4	14	35	0.20	33	18
336	41	1	marrie	08-Jul-1967	15.00	whitec	20	16	36	37	0.25	53	25
337	40	2	marrie	28-Apr-1968	6.00	nurse	20	18	38	48	0.26	66	25
338	26	1	single	01-Feb-1983	15.00	nurse	18	18	36	52	0.25	74	29
339	49	1	marrie	10-Feb-1959	12.00	whitecollar					0.21	35	14
341	55	1	marrie	09-Jan-1954	15.00	physician					0.27	44	16
342	49	2	marrie	06-Jul-1959	12.00	whitecollar					0.26	41	25
343	48	2	marrie	15-Jul-1960	12.00	whitec	18	11	29	50	0.29	54	18
344	41	1	marrie	27-Nov-1967	12.00	blueco	18	18	36	37	0.25	38	26
345	48	2	marrie	24-Jul-1960	12.00	whitec	18	18	36	43	0.29	62	27
346	27	2	single	26-Nov-1981	19.00	whitec	18	18	36	54	0.28	66	28
347	46	2	marrie	30-Nov-1962	6.00	whitec	20	16	36	63	0.28	68	26
348	48	1	marrie	25-Jul-1960	12.00	other					0.23	29	21
349	24	2	single	18-Jun-1984	15.00	blueco	18	20	38	53	0.31	83	29
350	50	1	marrie	17-Jun-1958	6.00	whitec	20	18	38	47	0.26	52	23
351	39	2	marrie	12-Dec-1969	6.00	whitec	20	20	40	52	0.24	62	33
352	27	1	single	11-Mar-1981	12.00	nurse	18	18	36	38	0.30	63	24
353	47	1	marrie	08-Jan-1962	12.00	bluecollar					0.26	41	23
355	51	1	marrie	16-Oct-1957	15.00	whitec	20	18	38	32	0.27	53	30
356	46	2	marrie	13-Sep-1962	15.00	blueco	20	18	38	37	0.29	57	26
357	47	1	marrie	12-Sep-1961	12.00	other	20	13	33	57	0.25	46	25
358	47	2	marrie	19-Jul-1961	6.00	whitec	20	20	40	60	0.34	71	32
359	50	2	marrie	02-Jun-1958	15.00	nurse	20	18	38	54	0.29	65	30
360	48	1	marrie	14-Mar-1960	12.00	blueco	20	13	33	58	0.29	48	20
361	51	2	marrie	14-Nov-1957	15.00	whitec	16	18	34	47	0.26	64	20
362	34	1	marrie	14-Sep-1974	6.00	nurse	18	18	36	47	0.28	68	31
364	53	1	marrie	16-Jul-1955	6.00	whitec	20	18	38	30	0.28	45	22
365	40	1	marrie	30-Sep-1968	12.00	whitec	18	18	36	20	0.25	44	22
366	51	2	marrie	15-Dec-1957	12.00	nurse	20	16	36	49	0.27	57	26
367	40	1	marrie	27-Mar-1968	6.00	physic	18	20	38	42	0.28	56	26
368	45	1	marrie	19-Jan-1964	12.00	blueco	18	16	34	28	0.22	52	24
369	33	1	marrie	10-Aug-1975	12.00	nurse	18	20	38	46	0.27	59	32
370	55	1	marrie	10-Nov-1953	12.00	whitec	20	20	40	32	0.25	50	27
371	46	1	marrie	16-Jan-1963	6.00	whitec	18	18	36	36	0.28	50	29
372	51	2	marrie	22-Mar-1957	15.00	whitec	20	18	38	45	0.26	67	26
373	50	1	marrie	05-Dec-1958	6.00	other	20	18	38	49	0.27	39	25
374	23	2	single	17-Dec-1985	15.00	other	18	18	36	32	0.28	64	29
375	45	2	marrie	15-Dec-1963	6.00	whitec	20	20	40	58	0.29	64	28
376	46	2	marrie	18-Dec-1962	6.00	whitec	20	20	40	46	0.31	36	28
377	46	1	marrie	20-Sep-1962	6.00	whitec	20	20	40	47	0.27	52	27
378	23	1	single	09-Mar-1985	6.00	whitec	18	13	31	66	0.29	69	29
380	32	1	marrie	29-Aug-1976	12.00	nurse	20	18	38	30	0.28	68	21
381	34	2	marrie	16-Jun-1974	19.00	physic	20	18	38	55	0.26	62	35
382	41	1	single	28-Feb-1967	9.00	blueco	16	13	29	45	0.25	32	20
383	24	1	single	15-May-1984	15.00	whitec	18	20	38	58	0.28	63	27
384	46	2	marrie	20-Nov-1962	15.00	whitec	20	9	29	46	0.24	71	21
385	41	1	marrie	12-Dec-1967	12.00	whitec	18	16	34	56	0.26	64	31

386	47	1	marrie	25-Nov-1961	15.00	nurse	20	16	36	43	0.25	44	20
387	45	1	marrie	07-Feb-1963	6.00	physic	20	18	38	40	0.29	62	26
388	29	1	single	15-May-1979	19.00	physic	20	18	38	73	0.26	75	30
389	43	2	marrie	30-Aug-1965	12.00	blueco	18	18	36	31	0.25	58	19
390	55	1	marrie	13-Jul-1953	6.00	physic	20	20	40	50	0.26	43	33

ss	ds	maze	bvmt	cf	eit	cpt	dspan	upsa	tmtt	sct	hvltt	sst	dst	mazet
9	14		15	15	10.17	1.13	10	19	37	49	51	42	48	
25	23	21	35	23	8.18	3.13	25	34	71	80	62	87	65	71
15	12	10	14	14	7.95	1.06			47	54	51	59	44	60
19	23	18	32	28	11.18	3.20	21	36	68	70	79	70	65	69
18	26	21	32	44	10.29	3.33	24	40	56	77	73	67	71	71
18	19	1	28	19	6.84	1.45	14	34	47	49	44	67	57	32
19	17	8	28	27	8.37	1.01	19	38	46	58	56	70	53	57
13	8	11	27	17	8.10	0.59	10	4	45	39	45	53	36	62
17	27	15	28	18	10.67	3.05	24	38	56	63	68	64	73	66
21	25	20	35	22	10.88	2.79	25	38	59	60	64	76	69	71
12	15		13	15	8.73	2.39	11	36	56	60	53	50	49	
16	13	16	26	25	7.87	2.51	15	38	66	56	58	62	46	67
13	14	12	18	17	5.74	1.65	14	27	52	49	44	53	48	63
7	21	5	31	21	9.23	1.63	18	32	60	55	60	36	61	50
9	11	9	13	22	7.18	2.20	15	34	61	52	51	42	42	59
10	21	7	8	19	9.83	2.29	21	34	56	49	56	44	61	55
17	17	5	19	17	8.00	2.13	15	31	47	58	58	64	53	50
19	24	11	28	18	10.58	2.17	19	36	68	58	64	70	67	62
12	17	8	29	23	7.54	1.77	18	34	69	63	66	50	53	57
11	19	16	17	26	8.18	2.42	12	21	63	53	62	47	57	67
12	16	5	12	20	8.11	1.08	14	15	44	44	53	50	51	50
19	18	24	33	20	8.47	2.28	18	38	71	67	75	70	55	74
9	22		25	30	8.53	1.67	14	36	47	46	62	42	63	
10	13	4	21	16	6.07	1.77	11	15	50	53	44	44	46	47
17	25	18	14	26	10.54	2.17	23	38	64	57	62	64	69	69
15	25	17	31	29	9.36	3.29	25	34	65	71	80	59	69	68
16	20	16	19	20	7.15	2.97	21	34	54	58	55	62	59	67
2	15	8	16	17	8.91	1.56	14	32	43	36	55	22	49	57
12	15	6	22	22	7.34	1.99	12	25	52	46	45	50	49	53
17	14	4	15	12	7.79	1.70	12	10	52	51	58	64	48	47
2	14		11	14	7.76	1.90	12	36	39	47	49	22	48	
19	22	4	27	20	9.61	3.05	16	36	70	64	58	70	63	47
14	24	12	16	21	7.62	2.86	18	29	51	64	60	56	67	63
11	15	14	29	13	7.52	2.49	10	32	43	46	60	47	49	65
9	25	7	23	23	9.00	2.20	18	36	60	56	55	42	69	55
14	16	12	16	18	6.75	2.13	15	23	65	53	53	56	51	63
12	14	10	18	16	7.20	1.33	17	31	64	46	56	50	48	60
16	26		28	19	7.11	2.48	17	36	66	56	56	62	71	
12	19	16	8	21	7.61	1.46	16	29	54	46	55	50	57	67
15	27	3	23	16	10.59	1.95	18	33	48	53	62	59	73	44
13	21	12	26	25	11.18	2.41	17	38	57	73	66	53	61	63
14	12	9	14	18	4.22	1.75	13	34	56	43	56	56	44	59
14	12	5	12	25	8.10	1.14	12	34	54	51	58	56	44	50
13	13	9	20	19	6.67	0.61	11	19	48	40	47	53	46	59
14	19	9	30	25	7.45	2.20	20	36	57	64	73	56	57	59
5	5	7	34	16	6.30	1.10	15	21	48	45	47	30	30	55
10	18	10	26	10	10.56	1.90	15	30	58	49	69	44	55	60
10	13	8	18	17	8.18	1.66	7	25	50	56	58	44	46	57
12	19	6	22	22	9.24	1.58	16	38	50	49	56	50	57	53
19	21	21	30	23	8.49	2.49	17	36	57	63	64	70	61	71
11	14	5	24	24	9.30	1.34	17	17	46	50	55	47	48	50
13	17		19	16	8.22	2.67	11	25	50	40	53	53	53	
20	27	21	25	28	9.05	3.50	19	38	78	65	56	73	73	71
10	21	9	27	17	8.04	1.87	15	32	52	60	62	44	61	59
15	22	12	21	18	10.11	2.99	22	29	56	63	64	59	63	63
14	18		21	22	10.10	1.21	18	30	44	47	51	56	55	
13	12		10	12	5.55	1.66	10	14	51	44	47	53	44	
16	17	14	18	18	8.09	1.84	12	33	48	49	56	62	53	65
13	15	8	17	27	9.36	0.96	17	23	46	61	60	53	49	57
13	14	8	22	13	8.27	0.70	12	15	34	37	45	53	48	57

16	20	5	22	19	8.45	2.81	12	32	50	68	62	62	59	50
16	15	10	25	25	6.62	2.31	16	34	54	38	51	62	49	60
10	15		13	15	7.42	1.44	11	31	43	40	53	44	49	
21	28	23	30	33	8.84	2.94	23	31	50	65	71	76	75	73
13	22	5	26	14	6.90	1.52	16	36	47	47	44	53	63	50
20	18	7	28	17	11.44	2.87	16	34	51	60	62	73	55	55
11	16	8	16	18	7.07	2.24	15	23	56	52	47	47	51	57
11	14	6	12	9	8.57	2.01	11	26	50	51	44	47	48	53
11	25	5	27	20	8.59	1.64	18	27	59	46	38	47	69	50
13	21	8	14	18	7.50	2.68	17	23	51	60	49	53	61	57
14	17	5	28	18	10.81	1.43	15	26	44	49	49	56	53	50
12	22	8	18	20	8.89	2.30	18	33	62	63	71	50	63	57
13	10	1	3	9	7.95	0.75	9	2	37	35	44	53	40	32
13	20	9	25	15	12.28	1.75	19	29	62	53	47	53	59	59
15	24	7	17	17	6.55	2.87	23	32	49	49	55	59	67	55
14	22	4	7	16	8.34	1.42	12	31	46	43	34	56	63	47
13	23	7	28	20	10.01	3.01	22	36	52	58	47	53	65	55
2	12	2	6	21	8.12	0.74	12	8	41		29	22	44	39
9	17	5	21	20	8.42	1.62	15	36	51	56	56	42	53	50
16	18	6	13	18	9.46	2.37	17	23	40	51	58	62	55	53
17	23	11	18	18	9.36	2.40	22	29	69	56	58	64	65	62
12	15	6	20	24	10.83	2.82	20	29	51	58	45	50	49	53
23	27	24	28	29	7.95	1.78	25	36	59	77	62	82	73	74
17	25	10	16	19	9.25	1.97	19	36	49	51	62	64	69	60
6	18	3	3	17	8.78	1.35	13	31	44	50	34	33	55	44
19	21	9	30	27	10.56	2.29	19	38	53	70	68	70	61	59
15	18	9	26	29	7.37	1.18	17	31	59	60	56	59	55	59
10	9	8	15	11	7.66	1.13	9	18	45	46	31	44	38	57
18	21	5	27	31	8.96	3.58	24	38	64	60	51	67	61	50
17	15	17	18	24	7.87	2.03	15	25	54	58	60	64	49	68
10	8	3	4	4	7.09	0.44	9	12	34	33	36	44	36	44
7	11	3	3	15	8.24	1.28	13	12	37	41	47	36	42	44
11	22	11	35	10	9.33	1.69	16	25	46	43	62	47	63	62
2	5	4	8	11	6.67	1.77	15	21	46	56	51	22	30	47
14	25	13	22	27	12.68	2.57	20	38	65	59	56	56	69	64
13	15	5	25	19	6.66	2.80	17	27	72	60	62	53	49	50
11	17	4	18	7	9.81	2.23	16	34	48	53	51	47	53	47
16	17	5	9	9	8.14	1.55	13	20	48	48	53	62	53	50
13	21	3	27	23	10.84	2.67	15	38	52	60	62	53	61	44
16	19	19	22	15	9.48	2.61	21	34	52	65	66	62	57	70
	16	1	19	7	7.23	0.80	8	15	39	44	49		51	32
10	14	5	7	16	8.57	1.47	15	27	50	49	44	44	48	50
14	21	9	20	28	10.47	3.09	16	38	53	58	53	56	61	59
15	4	2	32	16	7.60	1.67	12	28	51	49	56	59	28	39
17	18	16	11	13	8.63	1.04	15	13	45	43	47	64	55	67
16	24	14	29	27	9.82	3.35	19	36	60	60	69	62	67	65
17	18	7	21	14	6.51	1.22	17	29	57	59	49	64	55	55
5	15	5	21	25	10.62	1.16	12	16	60	49	53	30	49	50
16	18	7	13	16	6.36	2.88	18	38	60	52	58	62	55	55
13	12	3	5	25	7.37	2.47	11	27	43	40	53	53	44	44
15	20	9	4	15	6.30	2.25	18	21	52	45	40	59	59	59
16	18	11	16	37	7.88	2.05	14	25	51	44	58	62	55	62
7	13	5	15	26	7.83	1.58	12	27	56	55	53	36	46	50
15	11	2	8	19	7.19	1.35	14	28	55	39	47	59	42	39
9	11	2	6	14	7.91	1.83	10	25	42	44	34	42	42	39
12	4	4	6	18	10.15	1.53	12	21	42	39	36	50	28	47
8	8	6	15	12	5.88	0.70	6	10	36	46	44	39	36	53
6	13	11	15	19	9.35	1.80	19	38	63	46	45	33	46	62
17	28	4	31	22	7.57	2.96	15	40	58	63	66	64	75	47
7	9	2	8	11	6.43	0.57	7	21	32	39	47	36	38	39
12	18	3	14	20	10.60	1.08	13	36	50	46	49	50	55	44
11	10	4	21	12	8.78	1.38	12	23	50	47	53	47	40	47

9	15	1	12	10	9.15	1.86	13	17	60	56	51	42	49	32
15	13	5	7	23	8.39	1.62	16	36	49	49	53	59	46	50
18	23	7	21	19	9.18	2.04	15	34	38	49	55	67	65	55
9	23	2	18	21	11.14	2.64	16	29	54	50	51	42	65	39
9	24	7	20	13	9.06	1.80	13	23	57	51	49	42	67	55
17	24	10	21	21	7.30	1.32	13	16	45	51	53	64	67	60
6	13	3	17	17	4.94	1.19	15	36	46	43	44	33	46	44
15	19	13	24	21	7.32	3.96	18	38	53	68	66	59	57	64
18	23	7	27	27	8.66	2.65	27	40	59	60	64	67	65	55
17	25	9	22	21	7.17	2.82	27	33	56	74	68	64	69	59
19	17	9	17	18	9.87	1.65	16	25	50	55	62	70	53	59
19	13	9	17	18	7.18	1.51	15	25	44	38	60	70	46	59
12	17	11	28	18	8.98	2.66	16	36	63	60	53	50	53	62
16	25	9	14	11	5.92	2.12	12	36	61	51	55	62	69	59
15	19	7	21	12	9.72	2.61	20	36	53	63	58	59	57	55
13	16	1	8	5	7.61	1.55	15	14	38	48	49	53	51	32
19	24	22	29	29	8.17	2.16	17	36	54	62	62	70	67	72
11	18	25	23	33	10.39	2.46	17	38	56	71	62	47	55	74
20	27	14	25	18	9.88	3.01	24	36	55	77	69	73	73	65
14	14	4	13	17	5.91	1.30			41	49	42	56	48	47
14	20	16	17	23	6.96	2.97			59	56	45	56	59	67
16	18	21	33	24	10.28	1.87			58	53	62	62	55	71
15	23	19	24	25	6.89	2.67	20	29	65	63	49	59	65	70
13	17	8	21	17	9.94	1.93	21	36	55	51	64	53	53	57
20	12	21	25	19	7.80	2.54	17	36	66	68	66	73	44	71
20	24	23	30	19	9.46	3.39	22	36	64	71	68	73	67	73
15	26	19	27	18	10.65	3.73	21	36	64	72	64	59	71	70
16	21	5	19	17	8.21	1.51			45	45	55	62	61	50
22	26	16	34	33	9.50	3.95	21	38	72	83	69	79	71	67
19	28	21	27	27	7.14	2.70	18	38	56	61	58	70	75	71
22	25	13	33	32	11.17	2.77	27	40	51	68	77	79	69	64
18	25	25	24	30	8.11	2.95	21	36	70	69	60	67	69	74
18	19	7	20	23	10.51	2.49			55	53	58	67	57	55
15	21	19	27	26	11.08	2.38	19	38	62	62	71	59	61	70
18	23	16	28	21	9.19	3.43	20	38	67	65	64	67	65	67
20	20	18	20	15	8.11	2.19	19	33	55	57	62	73	59	69
19	19	25	14	18	10.54	2.83	20	40	83	74	75	70	57	74
16	23	19	33	26	8.84	3.29	23	38	68	70	71	62	65	70
17	11	25	13	25	6.16	2.28	15	33	65	58	53	64	42	74
12	15	19	24	18	5.16	2.37	17	34	58	70	53	50	49	70
20	26	21	29	29	8.74	3.31	26	36	63	72	73	73	71	71
16	26	10	17	33	9.33	3.10	21	38	65	56	56	62	71	60
15	21	17	27	18	9.07	2.21	20	36	53	56	56	59	61	68
15	18	10	25	18	8.55	2.29	13	36	59	65	64	59	55	60
15	23	15	25	24	10.98	2.97	18	38	64	64	64	59	65	66
17	21	14	22	20	8.44	1.97	18	34	45	61	60	64	61	65
16	28	22	31	38	8.94	3.58	18	38	61	66	75	62	75	72
17	18	10	16	15	8.25	2.46	16	40	54	60	66	64	55	60
15	25	21	23	44	7.69	2.52	14	36	63	60	69	59	69	71
17	20	10	29	22	9.16	3.33	23	38	58	72	64	64	59	60
15	25	12	20	35	10.99	2.65	17	38	60	52	62	59	69	63
20	21	18	28	23	11.86	2.23	15	36	63	70	69	73	61	69
19	23	18	23	21	8.78	2.40	21	40	68	70	68	70	65	69
15	24	10	23	19	10.63	3.51	19	40	74	50	68	59	67	60
16	19	13	25	28	8.26	2.53	22	40	59	61	66	62	57	64
20	26	23	30	24	8.82	3.61	29	31	65	73	69	73	71	73
19	19	25	24	23	10.95	2.12	19	38	65	72	55	70	57	74
14	25	26	35	29	11.34	3.36	22	38	57	68	80	56	69	75
16	16	8	26	15	7.62	1.65	14	29	53	47	53	62	51	57
13	22	19	32	25	8.77	2.62	22	38	65	69	66	53	63	70
16	18	20	28	20	6.73	3.27	15	29	50	74	55	62	55	71
21	25	18	32	16	9.89	3.29	23	34	56	70	73	76	69	69

14	23	13	20	25	10.95	2.47	27	36	55	56	53	56	65	64
17	24	9	25	21	12.09	3.17	27	38	66	68	64	64	67	59
21	28	20	28	35	8.14	3.53	27	38	58	77	71	76	75	71
23	18	16	20	26	10.17	2.04	20	36	52	65	51	82	55	67
17	21	14	26	26	9.42	2.77	17	40	56	55	77	64	61	65

bvmtt	cft	eitt	cptt	mccbt	stroopt	dspant	upsat	S1L	S2L	S1V	S2V	S2divi	SMOKE	Smokin
48	43	59.50	43			37	34.78	34.00	32.00	2.37	1.38	0.58	1	
73	58	48.80	64	78	65	78	55.21	54.00	62.00	2.99	0.55	0.18	2	8
47	41	47.57	42	49	58			64.00	40.00	2.72	0.00	0.00		
69	68	64.91	65	82	62	67	58.81	66.00	60.00	4.42	-0.83	-0.19	1	
69	98	60.13	67	83	69	75	66.63	56.00	80.00	3.33	0.00	0.00	2	13
64	51	41.59	46	48	53	48	55.21	54.00	56.00	5.17	3.30	0.64	1	
64	66	49.84	41	59	56	61	62.62	58.00	48.00	2.54	1.28	0.50	1	
63	47	48.37	37	48	51	37	25.91	56.00	54.00	2.26	1.04	0.46	1	
64	49	62.20	63	73	61	75	62.62	64.00	40.00	2.51	0.00	0.00	1	
73	57	63.30	61	77	65	78	62.62	64.00	64.00	5.04	1.42	0.28	1	
46	43	51.78	56		40	39	58.81	46.00	50.00	4.28	2.48	0.58	1	
62	62	47.16	58	63	47	50	62.62	56.00	50.00	2.65	0.40	0.15	2	35
52	47	35.73	48	48	38	48	44.24	54.00	46.00	3.94	1.60	0.41	2	10
68	55	54.46	48	58	60	59	51.82	46.00	50.00	3.36	0.53	0.16	2	30
46	57	43.46	54	50	42	50	55.21	52.00	50.00	7.74	2.59	0.33	2	20
40	51	57.68	55	54	35	67	55.21	52.00	50.00	3.87	2.24	0.58	2	
53	47	47.83	53	55	65	50	50.20	52.00	40.00	2.28	0.00	0.00	1	
64	49	61.70	54	70	51	61	58.81	60.00	52.00	7.25	1.77	0.24	2	10
65	58	45.36	50	61	62	59	55.21	54.00	58.00	4.00	-0.95	-0.24	1	
51	64	48.83	57	61	55	42	36.83	62.00	62.00	3.51	2.55	0.73	1	
44	53	48.42	42	47	46	48	31.28	54.00	54.00	4.98	3.41	0.68	1	
70	53	50.37	55	74	73	59	62.62	62.00	60.00	3.38	1.36	0.40	1	
61	72	50.70	48		52	48	58.81	52.00	48.00	5.64	2.66	0.47		
56	45	37.47	50	45	53	39	31.28	62.00	64.00	1.96	1.86	0.95	1	
47	64	61.49	54	67	57	72	62.62	56.00	60.00	4.92	0.67	0.14	2	27
68	70	55.16	66	78	62	78	55.21	66.00	68.00	2.07	0.08	0.04	1	
53	53	43.28	63	61	53	67	55.21	52.00	52.00	6.41	-1.27	-0.20	2	
49	47	52.73	47	46	52	48	51.82	50.00	74.00	3.53	0.00	0.00	1	
57	57	44.33	52	51	48	42	41.56	54.00	56.00	2.64	4.89	1.86	1	
48	38	46.73	49	51	32	42	28.07	54.00	58.00	3.93	1.89	0.48	2	
43	41	46.55	51		44	42	58.81	56.00	54.00	6.69	4.08	0.61	1	
63	53	56.52	63	66	61	53	58.81	62.00	60.00	4.46	2.21	0.50	2	37
49	55	45.82	61	62	60	59	47.12	48.00	48.00	2.55	1.75	0.69	2	4
65	40	45.28	57	57	47	37	51.82	58.00	64.00	1.48	0.12	0.08	1	
58	58	53.24	54	59	59	59	58.81	56.00	52.00	2.92	1.16	0.40	2	15
49	49	41.15	53	55	50	50	39.10	50.00	40.00	7.72	2.32	0.30	1	
52	45	43.54	45	52	53	56	50.20	56.00	64.00	3.67	1.14	0.31	1	
64	51	43.04	57		62	56	58.81	68.00	72.00	5.00	1.38	0.28	1	
40	55	45.77	46	52	52	53	47.12	52.00	48.00	7.85	4.84	0.62	2	30
58	45	61.74	51	60	41	59	53.49	48.00	48.00	7.16	2.06	0.29	1	
62	62	64.94	56	69	52	56	62.62	42.00	46.00	1.63	1.31	0.80	1	
47	49	27.56	49	47	31	45	55.21	54.00	80.00	2.26	0.00	0.00	2	30
44	62	48.40	43	50	47	42	55.21	56.00	70.00	0.79	0.00	0.00	2	
54	51	40.70	37	46	43	39	34.78	52.00	50.00	5.46	1.89	0.35	1	
67	62	44.90	54	65	57	64	58.81	52.00	52.00	6.52	2.26	0.35	1	
72	45	38.73	42	44	53	50	36.83	54.00	54.00	3.50	1.88	0.54	1	
62	34	61.59	51	61	51	50	48.63	64.00	66.00	1.42	-0.09	-0.06	1	
52	47	48.82	48	52	26	28	41.56	50.00	48.00	2.57	1.64	0.64	2	30
57	57	54.53	48	55	51	53	62.62	48.00	48.00	1.46	1.04	0.71	1	
67	58	50.49	57	70	56	56	58.81	62.00	58.00	2.93	0.70	0.24	1	
59	60	54.85	45	53	49	56	32.93	44.00	48.00	3.64	2.22	0.61	1	
53	45	49.04	59		53	39	41.56	60.00	56.00	2.85	-0.10	-0.03	1	
61	68	53.48	68	75	38	61	62.62	52.00	52.00	2.37	1.51	0.64	2	5
63	47	48.08	51	59	71	50	51.82	58.00	56.00	5.35	1.25	0.23	1	
56	49	59.18	63	67	53	70	47.12	52.00	40.00	2.99	0.00	0.00	2	16
56	57	59.12	43		51	59	48.63	52.00	52.00	10.50	5.04	0.48	1	
42	38	34.67	48		49	37	30.53	48.00	54.00	4.19	3.26	0.78	1	
52	49	48.34	50	57	48	42	53.49	62.00	68.00	0.75	0.85	1.14	1	
51	66	55.13	41	55	59	56	39.10	60.00	46.00	2.34	0.00	0.00	2	17
57	40	49.28	38	46	33	42	31.28	48.00	48.00	6.58	1.54	0.23	1	

57	51	50.27	61	61	67	42	51.82	52.00	58.00	3.50	0.42	0.12	1	
61	62	40.43	55	56	52	53	55.21	60.00	56.00	3.35	0.25	0.08	2	40
46	43	44.75	46		51	39	50.20	58.00	78.00	0.62	0.00	0.00	1	
67	77	52.35	62	78	76	72	50.20	54.00	52.00	7.32	6.49	0.89	1	
62	41	41.92	47	50	57	53	58.81	58.00	60.00	4.22	1.34	0.32	2	42
64	47	66.35	62	68	53	53	55.21	54.00	76.00	6.62	0.00	0.00	1	
49	49	42.86	55	51	51	50	39.10	50.00	46.00	3.17	1.65	0.52		
44	32	50.89	52	46	41	39	42.87	40.00	56.00	1.87	0.00	0.00	1	
63	53	51.02	48	53	53	59	44.24	62.00	60.00	2.69	1.50	0.56	1	
47	49	45.16	59	54	52	56	39.10	50.00	52.00	5.40	4.04	0.75	1	
64	49	62.92	46	55	35	50	42.87	54.00	74.00	1.62	0.00	0.00	2	40
52	53	52.65	55	63	59	59	53.49	54.00	54.00	4.20	1.56	0.37	1	
33	32	47.59	38	33	35	34	25.60	58.00	58.00	6.65	3.46	0.52	1	
61	43	70.82	49	61	69	61	47.12	54.00	56.00	3.41	1.36	0.40	1	
51	47	40.06	62	56	47	72	51.82	56.00	52.00	3.11	1.19	0.38	1	
38	45	49.67	46	43		42	50.20	50.00	60.00	2.26	0.68	0.30	2	35
64	53	58.63	63	62	57	70	58.81	52.00	66.00	2.58	0.00	0.00	1	
37	55	48.51	38		31	42	27.14	56.00	50.00	1.18	0.92	0.78	2	13
56	53	50.13	48	52	57	50	58.81	48.00	66.00	2.82	0.00	0.00	1	
46	49	55.67	56	55	59	56	39.10	52.00	52.00	6.43	4.25	0.66	1	
52	49	55.17	56	64	41	70	47.12	50.00	52.00	2.86	1.91	0.67	1	
54	60	63.07	61	58	67	64	47.12	56.00	52.00	4.69	1.72	0.37	1	
64	70	47.57	50	73	57	78	58.81	62.00	60.00	3.99	1.25	0.31	2	3
49	51	54.56	52	61	47	61	58.81	56.00	64.00	1.94	0.24	0.12	2	21
33	47	52.04	45	38		45	50.20	60.00	52.00	2.15	2.71	1.26	2	10
67	66	61.62	55	71	59	61	62.62	60.00	68.00	1.83	0.19	0.10	1	
62	70	44.45	43	59	58	56	50.20	58.00	60.00	2.86	0.83	0.29	1	
48	36	46.04	43	40	42	34	33.83	56.00	40.00	1.44	0.00	0.00	2	40
63	74	53.00	69	66	52	75	62.62	48.00	50.00	8.14	5.24	0.64		
52	60	47.17	52	61	53	50	41.56	56.00	54.00	2.84	0.93	0.33	1	
35	23	42.98	35	29	25	34	29.20	54.00	54.00	6.00	1.54	0.26	1	
33	43	49.15	44	37	30	45	29.20	40.00	40.00	2.23	2.04	0.91	1	
73	34	54.97	49	60	59	53	41.56	66.00	68.00	2.87	1.53	0.53	1	
40	36	40.71	50	37	53	50	36.83	58.00	54.00	4.22	0.92	0.22	1	
57	66	72.98	58	70	69	64	62.62	58.00	48.00	3.59	2.05	0.57	1	
61	51	40.64	61	59	55	56	44.24	56.00	48.00	5.02	2.22	0.44	1	
52	28	57.57	55	51	47	53	55.21	50.00	78.00	2.37	0.00	0.00		
41	32	48.61	47	48	60	45	35.78	60.00	44.00	6.15	0.00	0.00	1	
63	58	63.12	59	63	53	50	62.62	52.00	50.00	4.05	3.04	0.75	1	
57	43	55.81	59	66	47	67	55.21	60.00	62.00	4.18	1.00	0.24	2	2
53	28	43.72	39		47	31	31.28	60.00	52.00	4.01	1.06	0.27	1	
38	45	50.93	46	44	56	50	44.24	46.00	44.00	2.38	1.56	0.66	1	
54	68	61.12	64	64	47	53	62.62	50.00	48.00	7.17	5.27	0.74	1	
69	45	45.72	48	50	41	42	45.65	48.00	48.00	2.22	0.84	0.38	1	
43	40	51.25	42	51	40	50	29.84	60.00	40.00	2.74	0.00	0.00	1	
65	66	57.60	67	74	74	61	58.81	58.00	64.00	0.99	-0.64	-0.65	2	30
56	41	39.83	44	52	60	56	47.12	64.00	62.00	0.99	0.89	0.90	2	2
56	62	61.94	43	52	47	42	32.08	62.00	64.00	2.64	2.00	0.76	1	
46	45	39.02	62	55	44	59	62.62	56.00	52.00	3.37	1.32	0.39		
36	62	44.44	57	45	45	39	44.24	46.00	80.00	6.87	0.00	0.00	1	
35	43	38.70	55	46	48	59	36.83	48.00	66.00	2.70	0.00	0.00	1	
49	85	47.23	53	59	46	48	41.56	50.00	72.00	1.44	0.00	0.00	2	30
48	64	46.94	47	49	45	42	44.24	46.00	46.00	2.74	1.46	0.53	1	
40	51	43.52	45	41	68	48	45.65	48.00	48.00	8.31	7.10	0.85	1	
37	41	47.34	50	36	31	37	41.56	50.00	44.00	6.51	3.34	0.51	1	
37	49	59.42	47	40	55	42	36.83	58.00	58.00	2.70	2.05	0.76	1	
48	38	36.47	38	37	62	25	28.07	84.00	84.00	8.72	-0.14	-0.02	1	
48	51	55.11	50	50	46	61	62.62	56.00	58.00	11.03	3.18	0.29	1	
68	57	45.56	63	67	66	50	66.63	56.00	46.00	4.18	3.49	0.83	1	
40	36	39.43	36	32	50	28	36.83	50.00	48.00	2.82	1.54	0.55	2	30
47	53	61.84	42	49	51	45	58.81	54.00	50.00	3.86	1.43	0.37		
56	38	52.04	45	47	57	42	39.10	52.00	42.00	4.74	0.75	0.16	1	

44	34	54.03	51	45	51	45	32.93	54.00	52.00	4.36	-0.22	-0.05	1	
38	58	49.94	48	49	56	53	58.81	56.00	56.00	10.34	4.26	0.41	1	
56	51	54.20	53	58	37	50	55.21	62.00	46.00	2.65	0.00	0.00	1	
52	55	64.71	59	55	57	53	47.12	38.00	34.00	1.08	2.74	2.00	1	
54	40	53.55	50	54	27	45	39.10	58.00	54.00	3.69	0.47	0.13	1	
56	55	44.06	45	56	36	45	32.08	50.00	54.00	3.89	1.50	0.39	2	20
51	47	31.40	43	37	52	50	58.81	52.00	60.00	3.32	2.53	0.76	1	
59	55	44.21	73	67	65	59	62.62	50.00	50.00	2.82	1.42	0.50	1	
63	66	51.39	59	67	57	84	66.63	48.00	48.00	2.40	3.15	1.31		
57	55	43.39	61	66	67	84	53.49	52.00	80.00	2.05	0.00	0.00	2	10
51	49	57.92	48	60	67	53	41.56	54.00	50.00	4.96	0.95	0.19	2	
51	49	43.43	47	53	51	50	41.56	48.00	44.00	4.79	2.10	0.44	2	5
64	49	53.11	59	62	52	53	58.81	56.00	58.00	2.72	1.25	0.46	1	
47	36	36.68	53	54	48	42	58.81	44.00	40.00	6.31	2.86	0.45	1	
56	38	57.08	59	60	67	64	58.81	50.00	52.00	4.93	1.11	0.23	1	
40	24	45.73	47	39	47	50	30.53	62.00	60.00	5.14	0.09	0.02	1	
65	70	48.78	54	70	49	56	58.81	56.00	56.00	2.39	0.49	0.21	1	
58	77	60.70	57	69	58	56	62.62	46.00	42.00	2.40	1.12	0.47	1	
61	49	57.96	63	74	62	75	58.81	62.00	64.00	2.16	0.12	0.06	2	5
46	47	36.64	44	42				72.00	48.00	5.86	1.37	0.23	1	
51	58	42.29	63	58				66.00	44.00	6.59	5.97	0.90	1	
70	60	60.10	51	69				50.00	50.00	2.33	3.15	1.35	1	
59	62	41.89	59	63	60	64	47.12	68.00	84.00	3.08	1.52	0.49	1	
56	47	58.25	51	59	49	67	58.81	52.00	48.00	2.91	2.97	1.02	2	
61	51	46.76	58	67	54	56	58.81	50.00	50.00	4.56	3.11	0.68	1	
67	51	55.69	67	77	63	70	58.81	56.00	54.00	2.06	-0.36	-0.17	1	
63	49	62.08	71	75	71	67	58.81	54.00	46.00	2.86	1.96	0.68	1	
53	47	48.95	47	53				52.00	78.00	0.86	0.01	0.01	2	30
72	77	55.92	73	84	62	67	62.62	54.00	52.00	2.29	2.12	0.93	1	
63	66	43.20	60	69	57	59	62.62	52.00	58.00	1.51	-0.22	-0.14	1	
70	75	64.88	60	80	62	84	66.63	52.00	50.00	3.51	0.69	0.20	1	
59	72	48.42	62	73	50	67	58.81	70.00	84.00	1.01	0.92	0.91	1	
54	58	61.34	57	63				62.00	68.00	2.77	0.58	0.21	2	22
63	64	64.38	56	73	45	61	62.62	48.00	50.00	1.77	2.66	1.50	2	33
64	55	54.23	68	73	49	64	62.62	66.00	64.00	2.09	1.74	0.83	1	
54	43	48.44	54	63	66	61	53.49	46.00	64.00	3.51	0.35	0.10	1	
47	49	61.49	61	74	68	64	66.63	50.00	68.00	3.65	-1.20	-0.33	1	
70	64	52.35	66	76	63	72	62.62	58.00	58.00	5.84	2.14	0.37	1	
46	62	37.96	55	58	67	50	53.49	62.00	64.00	4.21	4.52	1.07	2	33
59	49	32.60	56	57	57	56	55.21	50.00	52.00	5.07	3.04	0.60	1	
65	70	51.83	66	78	57	81	58.81	60.00	56.00	1.43	1.29	0.90	2	14
51	77	55.00	64	67	43	67	62.62	52.00	44.00	2.17	2.15	0.99	2	35
63	49	53.57	54	64	35	64	58.81	66.00	62.00	2.86	1.97	0.69		
61	49	50.80	55	63	59	45	58.81	64.00	56.00	2.91	3.45	1.19	1	
61	60	63.84	63	72	53	59	62.62	60.00	62.00	2.53	0.52	0.21	2	20
57	53	50.19	52	62	41	59	55.21	58.00	64.00	4.20	0.77	0.18	2	31
68	87	52.92	69	80	57	59	62.62	52.00	76.00	2.60	0.60	0.23	2	20
49	43	49.19	57	60	45	53	66.63	60.00	56.00	3.76	0.27	0.07	2	38
58	98	46.20	58	72	48	48	58.81	54.00	52.00	3.08	0.98	0.32	2	
65	57	54.08	66	70	56	72	62.62	56.00	56.00	3.49	0.96	0.28		
54	81	63.91	59	69	59	56	62.62	50.00	52.00	2.62	0.59	0.23	1	
64	58	68.59	55	75	45	50	58.81	66.00	58.00	3.27	2.20	0.67	1	
58	55	52.02	56	71	67	67	66.63	52.00	54.00	3.38	1.42	0.42	1	
58	51	61.99	68	71	57	61	66.63	54.00	52.00	1.65	-0.29	-0.17		
61	68	49.23	58	67	57	70	66.63	50.00	48.00	1.63	0.98	0.60	1	
67	60	52.25	70	78	73	89	50.20	62.00	60.00	0.83	0.06	0.07	1	
59	58	63.68	53	70	43	61	62.62	50.00	78.00	4.17	0.08	0.02	1	
73	70	65.80	67	82	64	70	62.62	52.00	44.00	1.80	0.61	0.34	1	
62	43	45.83	48	55	56	48	47.12	48.00	44.00	2.37	2.37	1.00	1	
69	62	51.97	59	71	67	70	62.62	54.00	54.00	3.71	1.09	0.29		
64	53	41.03	66	65	57	50	47.12	56.00	54.00	2.17	0.34	0.16	1	
69	45	58.01	66	77	65	72	55.21	58.00	52.00	2.53	1.90	0.75	2	

54	62	63.69	57	64	54	84	58.81	68.00	60.00	2.38	0.99	0.42	2		
61	55	69.81	65	73	52	84	62.62	64.00	56.00	3.06	-0.08	-0.02	1		
64	81	48.60	69	79	79	84	62.62	52.00	54.00	6.37	1.86	0.29	1		
54	64	59.51	52	66	44	64	58.81	54.00	62.00	4.96	2.00	0.40	1		
62	64	55.47	60	71	60	56	66.63	64.00	46.00	4.97	0.87	0.17	2	25	

smoking amount
20
2
7
10
15
5
5
10
20
3
35
20
20
20
5
20
9

15

20

20

6

7

10
10
40

40

10

15
10

10

15

20

4

2

10

20

16
8

20

10
10

1
15
20
10
